# Still Acting Green: Continued Expression of Photosynthetic Genes in the Heterotrophic Dinoflagellate *Pfiesteria piscicida* **(**Peridiniales, Alveolata**)**

**DOI:** 10.1371/journal.pone.0068232

**Published:** 2013-07-16

**Authors:** Gwang Hoon Kim, Hae Jin Jeong, Yeong Du Yoo, Sunju Kim, Ji Hee Han, Jong Won Han, Giuseppe C. Zuccarello

**Affiliations:** 1 Department of Biology, Kongju National University, Kongju, Korea; 2 School of Earth and Environmental Science, Seoul National University, Seoul, Korea; 3 School of Biological Sciences, Victoria University of Wellington, Wellington, New Zealand; Institut de Génétique et Développement de Rennes, France

## Abstract

The loss of photosynthetic function should lead to the cessation of expression and finally loss of photosynthetic genes in the new heterotroph. Dinoflagellates are known to have lost their photosynthetic ability several times. Dinoflagellates have also acquired photosynthesis from other organisms, either on a long-term basis or as “kleptoplastids” multiple times. The fate of photosynthetic gene expression in heterotrophs can be informative into evolution of gene expression patterns after functional loss, and the dinoflagellates ability to acquire new photosynthetic function through additional endosymbiosis. To explore this we analyzed a large-scale EST database consisting of 151,091 unique sequences (29,170 contigs, 120,921 singletons) obtained from 454 pyrosequencing of the heterotrophic dinoflagellate *Pfiesteria piscicida*. About 597 contigs from *P. piscicida* showed significant homology (E-value <e^−30^) with proteins associated with plastid and photosynthetic function. Most of the genes involved in the Calvin-Benson cycle were found, genes of the light-dependent reaction were also identified. Also genes of associated pathways including the chorismate pathway and genes involved in starch metabolism were discovered. BLAST searches and phylogenetic analysis suggest that these plastid-associated genes originated from several different photosynthetic ancestors. The Calvin-Benson cycle genes are mostly associated with genes derived from the secondary plastids of peridinin-containing dinoflagellates, while the light-harvesting genes are derived from diatoms, or diatoms that are tertiary plastids in other dinoflagellates. The continued expression of many genes involved in photosynthetic pathways indicates that the loss of transcriptional regulation may occur well after plastid loss and could explain the organism's ability to “capture” new plastids (i.e. different secondary endosymbiosis or tertiary symbioses) to renew photosynthetic function.

## Introduction

The genetic outcomes of plastid gain and loss have been actively investigated. Dinoflagellates together with apicomplexans [1]–[3] and ciliates [4], [5] have drawn special attention in terms of plastid evolution. Evidence is accumulating that the ancestor of these lineages was photosynthetic [6]. Although a photosynthetic ancestor postulated for a larger group, the Chromoalveolates [7], [8] have been challenged recently [9], [10]. These results are originally based on the discovery of genes associated with photosynthetic organelles found in some non-photosynthetic lineages (e.g. apicomplexans) [1].

While the ciliates and most apicomplexans are non-photosynthetic, about 50% of dinoflagellates are photosynthetic. The majority of photosynthetic dinoflagellates contain peridinin as an accessory pigment, but the origin of the peridinin plastid is still enigmatic. Yoon et al. [11] suggested haptophyte origin of peridinin plastid but others suggested its red algal origin by secondary endosymbiotic event [12]. Some dinoflagellates replaced the peridinin-containing plastid with others either from a green alga, cryptophytes, haptophytes or diatoms via tertiary endosymbiosis events or a second secondary endosymbiotic event [13]. This unique evolutionary gain and loss and regain of plastids among dinoflagellates [14] and the transfer of genes to the nucleus have lead to plastid-derived genes, mostly genes involved in photosynthesis or other critical plastid functions, from several lineages. For example, in the fucoxanthin-containing dinoflagellate *Karlodinium veneficum* some photosynthetic genes have a peridinin-dinoflagellate origin and some are more closely related to haptophyte genes [15]. These metagenomic conclusions have recently been challenged due to undue care in phylogenetic interpretation [10], [16].

In apicomplexans, photosynthetic ability has been lost, presumably over long evolutionary time scales, except for the early diverging apicomplexan *Chromera* which is sister to the remaining parasitic apicomplexans [17], and many genes involved in photosynthetic function have been lost, or are no longer expressed, from both the plastid (apicoplast) genome and the host nucleus.

While photosynthesis is relatively easy to document the presence of cryptic plastids (retention of plastids that lack photosynthetic pigments and light-energy derived carbon fixation) is more difficult. Plastids in many lineages are degenerate and difficult to recognize and are considered as “cryptic” plastids [18]. The confirmation of a cryptic plastid (the apicoplast) in an extremely well studied group of parasite, the apicomplexans, is only about 20 years old [19], revealing that plastids are difficult to confirm in non-photosynthetic lineages.

The heterotrophic dinoflagellates obtain primary carbon by ingesting other organisms [20] and loss of photosynthesis has occurred multiple times in dinoflagellate evolution [14]. It appears that some of the lineages diverging early in dinoflagellate evolution are non-photosynthetic [14], [21] again suggesting that they been without photosynthetic function for a long time. For example, recent studies on a heterotrophic early-diverging dinoflagellate *Oxyrrhis marina* indicate that it has a few genes for several biosynthetic pathways that are associated with plastids [22] but no genes associated with photosynthesis (e.g. the light harvesting or Calvin-Benson cycle), which may indicate an early ancestor with photosyhthesis, but the loss of many unused plastid functions over a long period. The *Crypthecodinium cohnii* (Gonyaulacales), a non-photosynthetic but later diverging dinoflagellate, has some genes associated with photosynthesis (e.g. ribulose-1,5-bisphosphate carboxylase/oxygenase) [23]. This species and *Oxyrrhis marina* [22] clearly show that they originally derived from a photosynthetic ancestors, with the transfer of the photobiont genes to the host nucleus, and that these transferred genes may still serve a function. Clearly plastids have functions beyond light-energy capture.

Still very little is known about how and why photosynthesis is lost outright or what occurs when photosynthetic function is no longer needed [24], [25]. The expression of genes may be a process that continues even after their functional utility has been lost. The selective advantage of reducing expression of these genes may be negligible and loss of function could be a stochastic process taking a long time. The continued expression of genes that have a plastid function, and plastid targeting, may aid in acquisition of alternate plastid, as has occurred often in dinoflagellates [5], [26]. Heterotrophic dinoflagellates therefore may make good candidates to study these stages of gene regulation once functional constraints (e.g. photosynthesis) have been removed. With this in mind we studied the heterotrophic dinoflagellate *Pfiesteria piscicida* using a large-scale EST data set to estimate expression of plastid genes and try to account for genes in important plastid biosynthetic pathways.

*P. piscicida* is a member of the family Pfiesteriaceae in the order Peridinales [27], a group containing mainly non-photosynthtic dinoflagellates. *P. piscicida* has been studied over the last 20 years as it is involved in fish deaths. While some aspects of its biology (life cycle and toxicity) are still controversial [28], [29], it is clear that *P. piscicida* is not photosynthetic and a TEM study could not find any membranous structures assignable as plastids [29]. *P. piscicida* has been implicated as being kleptoplastidic when it feeds upon cryptophyte algae as they have been shown to persist in vacuoles of starved *P. piscicida* for a week, apparently fixing carbon and accumulating starch [30].

In the present study, we identified many plastid-derived genes from both of the major photosynthetic pathways (light-dependent reaction and Calvin-Benson cycle) and other plastid-associated pathways (e.g. chorismate pathway).

## Materials and Methods

### Culture conditions

The strain of *P. piscicida* was originally isolated from Masan Bay (southern part of Korea) in July 2005 [31]. *P. piscicida* cells were added to 1-L polycarbonate (PC) bottles containing fresh medium. Bottles were capped, placed on a rotating wheel, incubated under an illumination of 20 µE/m^2^/s provided by cool-white fluorescent light on a 14∶10 h light-dark cycle. Perch blood cells were collected from the live fish purchased at seafood market. The serum was removed by washing the fish blood three times using PBS buffer after mild centrifugation. The washed blood cells were provided to *P. piscicida*. As the concentration of *P. piscicida* increased, cells were transferred to new 1-L PC bottles every 2 days and 1 mL of washed Perch blood cells were provided together. The fish blood cells were checked with fluorescence microscope every time. Although bacterial mRNA data could be easily eliminated from the EST dataset of *P. piscicida* because they do not have a poly-A tail of eukaryotic organism, the cultures were maintained with no visible bacterial contamination. We obtained forty 1-L bottles of dense culture of *P. piscicida* for pyrosequencing in 6 months. Samples were taken from the culture whenever they were transferred to other bottles and observed with a light microscope as well as a fluorescent microscope to check for contamination of any phototropic algae. Whenever any auto-fluorescence of photosynthetic phytoplankton was detected, whole culture batches were discarded and a new culture started. Eventually, we conducted these experiments using cultures without any algal contamination. In addition, to exclude possible contamination by other *Pfiesteria*-like heterotrophic dinoflagellates (so called PLDs) such as *Stoeckeria* spp., *Luciella* spp. and etc., PCR was performed using the DNA specific primers for detecting dinoflagellates before harvest [32].

### RNA isolation and pyrosequencing

Total RNA from *P. piscicida* was isolated using Trizol (MRC Inc.) according to manufacturer's protocol. Twenty liters of *P*. *piscicida* cells were taken after 3 days of starvation and harvested by centrifugation at 1200*g*. After confirming no contamination using fluorescence microscope and PCR, the pellets were immediately frozen with liquid nitrogen and stored at –80°C. Isolated RNA was quantified spectrophotometrically or using RNA gel electrophoresis. mRNA was purified using Oligotex (Qiagen) following the manufacturer's instructions. Double-strand cDNA was synthesized using Just cDNA Double-stranded cDNA Synthesis Kit (Agilent Technologies, CA, USA) following the manufacturer's instructions. The cDNA was then sent to GNCBio company (Daejeon, Korea) for 454 pyrosequencing. The library preparation, GS-FLX titanium sequencing, assembly and annotation of sequencing data were carried out by GNCBio. To analyze the sequence data a web-based pipeline program for EST data analysis was established (http://genebank.kongju.ac.kr).

### Identification of plastid-derived genes and bioinformatics

Possible contamination of bacterial mRNA was removed easily from the ESTs database. Putative plastid-derived genes were identified by closest sequence similarity with an E-value <1e^−30^. Nucleotide and amino acid sequence homology searches and comparison were carried out using BLAST on the NCBI GenBank database (http://blast.ncbi.nlm.nih.gov). Additional homology searches were carried out by comparing our translated EST database directly with the comprehensive chloroplast protein database of *Arabidopsis thaliana* [33] (Plastid protein database: http://www.plprot.ethz.ch, AT_Chloro database: http://www.grenoble.prabi.fr/at_chloro]. The sequences generated in this study were deposited in GenBank under accession number SRR837773.

### Phylogenetic analysis

Putative plastid-derived translated sequences of *P*. *piscicida* were aligned with the highest BLAST hit sequences plus other genes homologs from a selection of other lineages, especially stramenopiles, alveolates and plants were available. Prokaryote homologs were used as outgroup sequences. Sequences were aligned with MAFFT [34] in the Geneious software package [35]. Amino acids datasets were analyzed under the WAG+Γ +I model. The phylogeny of putative plastid-derived genes was inferred by maximum likelihood (ML) using RAxML 7.2.8 [36] and Bayesian analysis using MrBayes [37]. Likelihoods were estimated using the WAG protein substitution model [38]. For ML, bootstrap support was performed with 100 replicates. For Bayesian analysis, a total of 1,000,000 generations were run and sampled every 1,000 generations with burn-in of 100,000 generations. Stationarity was assessed using Tracer v1.5 [39] and a burn-in of 1000 generations was applied.

### 5′-RACE PCR

Total RNA was isolated as described above. First and second strand cDNA was synthesized using SMART cDNA Library construction kit (Clontech, CA, USA) according to manufacturer's protocol. Synthesized cDNA was used as a template. Specific primers were designed using the contigs sequence (Table S1). PCR was carried out in a 50 μL reaction mixture containing DNA template, 20 *p*mole Spliced Leader primer and 20 *p*mole specific primers (Table S1), 1X Taq buffer, 2.5 mM MgCl_2_, and 1 unit of Taq DNA polymerase (Takara, Tokyo, Japan). PCR was performed for 35 cycles at 95°C for 20 sec, 50–55°C for 30 sec, and 72°C for 60 sec, followed by 72°C for 10 min. The PCR products were cloned into T-easy cloning vector (Promega, USA), and their sequences were determined using 3730xl DNA analyzer (Applied Biosystem).

Sub-cellular localization was predicted using CBS prediction program (http://www.cbs.dtu.dk/services).

### Ethics statement

This research has been approved by Institutional Animal Care and Use Committees of Seoul National University.

## Results

**Figure 1:**
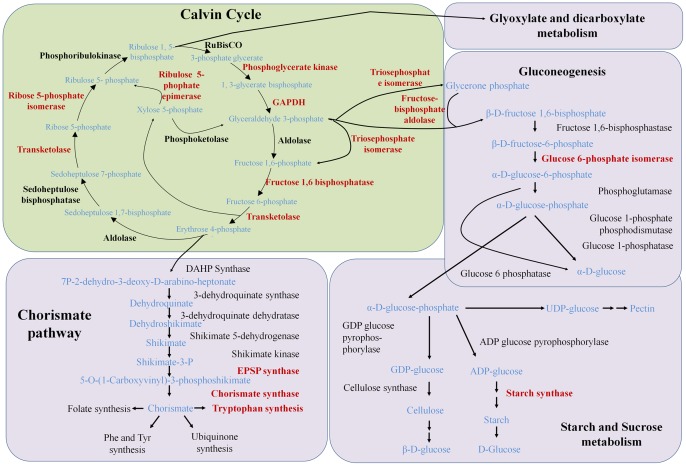
Calvin-Benson cycle and associated pathways. The genes found in *Pfiesteria piscicida* EST database were marked in red color.

**Figure 2:**
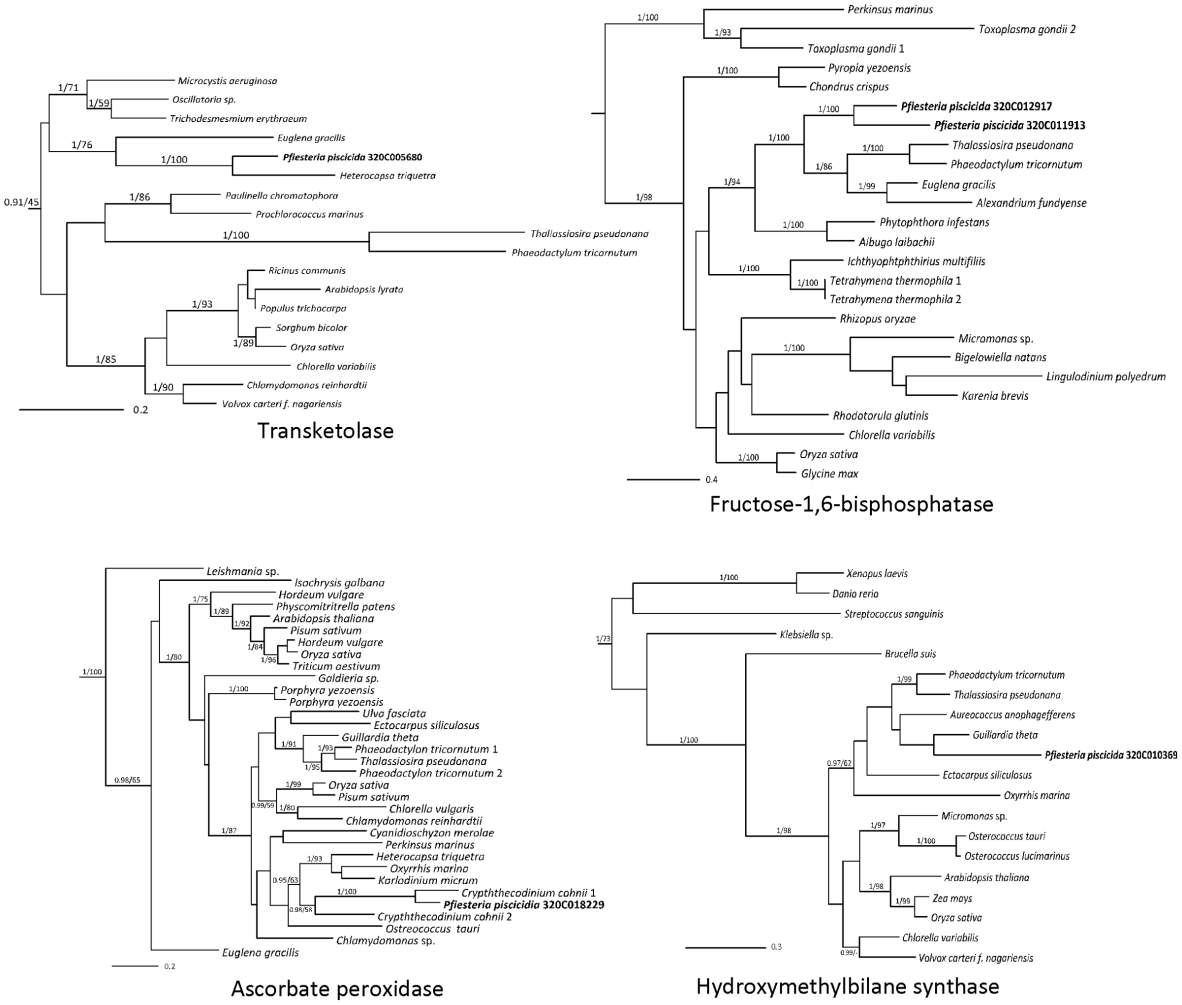
Maximum likelihood trees (WAG + I + Γ model) inferred from *Pfiesteria piscicida* protein sequences and assorted “protistan” lineages. Numbers above nodes indicate posterior probabilities and RAXML bootstrap percentages. * = 1.00 PP and 100% RAxML BP. Values <50% are not shown.

**Figure 3:**
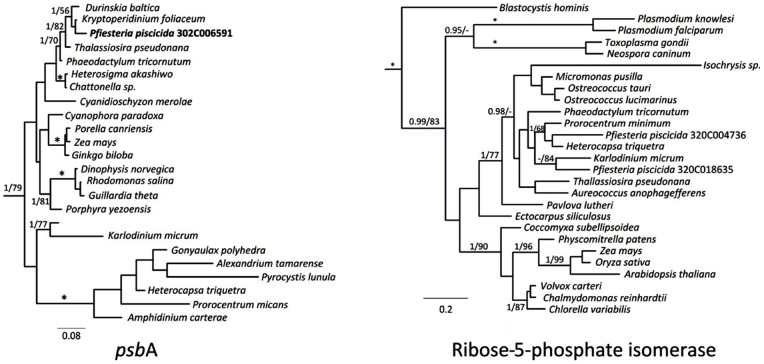
Maximum likelihood trees(RaXML, WAG + I + Γ model) inferred from *Pfiesteria piscicida* protein sequences and assorted plastid endosymbiont lineages. *psb*A protein – alignment length 282 amino acids. Ribose-5-phosphate isomerase protein – alignment length 231 amino acids. Numbers above nodes indicate posterior probabilities and RAXML bootstrap percentages. * = 1.00 PP and 100% RAxML BP. Values <50% are not shown.

A total of 264 Mbp of ESTs were sequenced from *P. piscicida* and assembled to 151,091 unique clusters (29,170 contigs and 120,921 singletons) with an average length of 636 bp. When photosynthesis genes were searched in the databases, 8 major genes (57 contigs) involved in the Calvin-Benson cycle, out of 13 main genes usually placed in the cycle, were detected (Fig. 1). Four genes involved in chorismate pathway and starch metabolism were also detected. These Calvin-Benson cycle genes had several isoforms with BlastX hits not always to the same species (Table 1). For example, triose phosphate isomerase isoforms had hits to Dinophyta (Table 1). Fructose-1,6-bisphosphotase had most similarity to Heterokontophyta genes. This is partially supported in the phylogeny in which the isoforms form a clade with diatoms, to the exclusion of some, but not all Dinophyta (Fig. 2). Most Calvin-Benson cycle genes had either hits exclusively to Dinophyta genes, or contained a majority of hits to Dinophyta genes. The phylogeny of the two isoforms of ribose 5-phosphate isomerase was mostly poorly resolved, one isoform grouped (1.0 PP, 68% BP) with the peridinin-containing dinoflagellates (*Heterocapsa* and *Prorocentrum*) to the exclusion of other organisms (Fig. 3). While the alternate isoform showed no strong relationship but grouped weakly with a dinoflagellate containing a haptophyte plastid (*Karlodinium*). Transketolase grouped strongly with Dinophyta. An alternate isoform had unsupported relationships to any other member and appeared to be highly divergent.

**Table 1:** Photosynthesis genes involved in Calvin cycle and its associated pathways in *Pfiesteria piscicida.*

Gene match	Contig (KNU ID)	No. of reads	Length (bp)	GenBank match (Acc. No.)	E-value (BlastX)	Origin	Phylum
Calvin cycle							
Ribose-5-phosphate isomerase	320C004736	6	809	AAW79354	1.44E-96	*Heterocapsa triquetra*	Dinophyta
	320C018635	7	712	AAW79354	5.95E-108	*Heterocapsa triquetra*	Dinophyta
Ribulose-phosphate 3-epimerase	320C010448	10	829	XP_002907019	1.05E-91	*Phytophthora infestans*	Oomycota
Phosphoglycerate kinase	320C003700	12	719	AAW79324	1.88E-76	*Heterocapsa triquetra*	Dinophyta
	320C003327	11	897	BAE07167	1.55E-157	*Karenia brevis*	Dinophyta
	320C004509	9	301	AAW79324	1.56E-45	*Heterocapsa triquetra*	Dinophyta
	320C004555	40	2039	BAE07174	0	*Heterocapsa triquetra*	Dinophyta
	320C005438	44	1699	AAW79324	0	*Heterocapsa triquetra*	Dinophyta
	320C015232	15	538	EGZ09335	9.22E-58	*Phytophthora sojae*	Oomycota
	320C019628	30	528	BAE07174	2.23E-77	*Heterocapsa triquetra*	Dinophyta
	320C019872	45	1233	XP_002771110	0	*Perkinsus marinus*	Dinophyta
	320C019994	48	1372	AAU20794	0	*Heterocapsa triquetra*	Dinophyta
	320C029471	23	741	AAW79324	2.30E-127	*Heterocapsa triquetra*	Dinophyta
	320C029472	8	625	AAW79324	1.50E-116	*Heterocapsa triquetra*	Dinophyta
	320C029883	19	671	AAU20794	8.29E-127	*Heterocapsa triquetra*	Dinophyta
	320C030136	21	592	AAW79324	2.81E-105	*Heterocapsa triquetra*	Dinophyta
Triose phosphate isomerase	320C001323	44	1207	XP_002785920	2.52E-90	*Perkinsus marinus*	Dinophyta
	320C010455	26	553	XP_002785920	2.90E-55	*Perkinsus marinus*	Dinophyta
	320C015974	6	610	EGB07870	1.73E-70	*Aureococcus anophagefferens*	Heterokontophyta
	320C019342	11	369	XP_002776078	1.06E-40	*Perkinsus marinus*	Dinophyta
Transketolase	320C005680	7	553	AAW79357	2.04E-88	*Heterocapsa triquetra*	Dinophyta
	320C008908	29	2188	ABP35605	0	*Karlodinium micrum*	Dinophyta
	320C023136	6	670	AAW79357	6.80E-68	*Heterocapsa triquetra*	Dinophyta
Fructose-1,6-bisphosphatase	320C011913	13	1030	EGB05300	7.51E-114	*Aureococcus anophagefferens*	Heterokontophyta
	320C012917	10	970	EGB05300	7.00E-107	*Aureococcus anophagefferens*	Heterokontophyta
Fructose-bisphosphate aldolase	320C003750	48	1224	ACU44982	0	*Pfiesteria piscicida*	Dinophyta
	320C013991	14	1270	ZP_09081006	1.98E-73	*Mycobacterium thermoresistibile*	Actinobacteria
	320C018990	41	247	ACU44985	1.78E-44	*Pfiesteria piscicida*	Dinophyta
	320C022296	37	603	ZP_09685324	9.32E-80	*Mycobacterium tusciae*	Actinobacteria
	320C025487	4	654	NP_001242086	1.57E-51	*Glycine max*	Steptophyta
	320C026884	9	451	ACU44982	5.96E-86	*Pfiesteria piscicida*	Dinophyta
	320C029593	173	956	ACU44982	0	*Pfiesteria piscicida*	Dinophyta
	320C029818	9	428	ACU44985	8.91E-47	*Pfiesteria piscicida*	Dinophyta
Glyceraldehyde-3-phosphate dehydrogenase	320C000795	23	442	ABI14256	1.42E-71	*Pfiesteria piscicida*	Dinophyta
	320C001994	31	1028	ABI14256	0	*Pfiesteria piscicida*	Dinophyta
	320C002033	34	839	ABI14256	4.72E-156	*Pfiesteria piscicida*	Dinophyta
	320C005475	54	1163	ABI14256	0	*Pfiesteria piscicida*	Dinophyta
	320C006386	9	379	ABI14256	4.22E-72	*Pfiesteria piscicida*	Dinophyta
	320C006750	17	1029	ABI14256	0	*Pfiesteria piscicida*	Dinophyta
	320C013130	5	558	AAM68968	7.23E-105	*Pyrocystis lunula*	Dinophyta
	320C018125	31	332	ABI14256	2.91E-70	*Pfiesteria piscicida*	Dinophyta
	320C019394	16	323	ABI14256	5.71E-66	*Pfiesteria piscicida*	Dinophyta
	320C019399	17	373	AAD01872	4.67E-61	*Gonyaulax polyedra*	Dinophyta
	320C019466	11	526	ABI14256	1.07E-99	*Pfiesteria piscicida*	Dinophyta
	320C019568	33	268	ABI14256	1.74E-33	*Pfiesteria piscicida*	Dinophyta
	320C019569	36	568	ABI14256	3.70E-107	*Pfiesteria piscicida*	Dinophyta
	320C019675	70	1155	ABI14256	0	*Pfiesteria piscicida*	Dinophyta
	320C021271	26	460	ABI14256	5.80E-98	*Pfiesteria piscicida*	Dinophyta
	320C021272	18	305	ABI14256	1.41E-64	*Pfiesteria piscicida*	Dinophyta
	320C024424	29	1152	ABI14256	0	*Pfiesteria piscicida*	Dinophyta
	320C028250	17	235	ABI14256	4.08E-30	*Pfiesteria piscicida*	Dinophyta
	320C028251	27	598	ABI14256	6.27E-124	*Pfiesteria piscicida*	Dinophyta
	320C028711	43	449	ABI14256	3.86E-72	*Pfiesteria piscicida*	Dinophyta
	320C029251	39	507	ABI14256	2.84E-103	*Pfiesteria piscicida*	Dinophyta
	320C029375	52	306	ABI14256	2.80E-64	*Pfiesteria piscicida*	Dinophyta
	320C029606	17	323	ABI14256	2.27E-65	*Pfiesteria piscicida*	Dinophyta
	320C029641	30	307	ACU45110	2.26E-42	*Pfiesteria piscicida*	Dinophyta
Chorismate pathway							
EPSP synthase	320C004635	3	468	CBN78624	1.89E-26	*Ectocarpus siliculosus*	Heterokontophyta
Chorismate synthase	320C003600	19	1113	XP_002773541	9.95E-138	*Perkinsus marinus*	Dinophyta
Tryptophane synthase (alpha/beta chain)	353S011114	1	512	CBQ69006	2.55E-57	*Sporisorium reilianum*	Basidiomycota
	320C002990	28	1622	EGD82890	1.40E-151	*Salpingoeca* sp.	Choanozoa
	320C004481	10	698	CBN77109	1.14E-75	*Ectocarpus siliculosus*	Heterokontophyta
	320C019217	6	657	EKX40150	5.30E-89	*Guillardia theta*	Cryptophyta
Gluconeogenesis							
Glucose-6-phosphate isomerase	320C002437	28	836	ABH11438	8.00E-167	*Pyrocystis lunula*	Myzozoa
	320C027875	12	516		3.54E-81		
	320C011436	5	472	ABH11437	8.05E-53	*Lingulodinium polyedrum*	Dinophyta
Starch and sucrose metabolism							
Soluble starch synthase	320C001989	14	1781	XP_004307998	3.14E-75	*Fragaria vesca*	Steptophyta
	320C000390	24	1506	EKX37680	1.47E-110	*Guillardia theta*	Cryptophyta
	320C018048	66	2042	EKX45880	4.44E-118		
Total:	69	1736					

**Figure 4:**
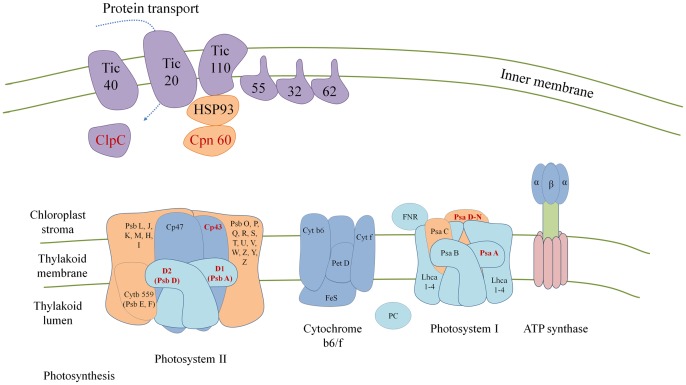
A diagram showing chloroplast membrane genes and light reaction center genes. The genes found in *Pfiesteria piscicida* EST database are marked in red color.

Although *P. piscicida* does not have any visible chloroplast several genes annotated for light reaction center were also detected (Fig. 4). Most light-dependent reaction genes showed high similarity with those of photosynthetic dinoflagellates containing diatoms as endosymbionts (the so called dinotoms, e.g. *Durinskia*, Table 2) [40]. Our phylogenetic relationships clearly place the *psb*A isoform with this Heterokontophyta to the exclusion of Dinophyta (Fig. 3).

**Table 2:** Light-dependent reaction genes expressed in *Pfiesteria piscicida*.

Gene match	Contig (KNU ID)	No. of reads	Length (bp)	GenBank match (Acc. No.)	E-value (BlastX)	Origin	Phylum
Photosystem II reaction center protein D1 (*psb*A)	320C006591	8	894	YP_874444	2.8E-168	*Phaeodactylum tricornutum*	Heterokontophyta
Photosystem II D2 (*psb*D)	353S009155	1	505	ACA49204	5.0E-87	*Kryptoperidinium foliaceum*	Dinophyta/Heterokontophyta
Photosystem I P700 chlorophyll a apoprotein A (*psa*A)	353S013648	1	294	YP_004072597	3.65E-45	*Thalassiosira oceanica*	Heterokontophyta
	353S005742	1	429	YP_003734951	1.07E-81	*Durinskia baltica*	Dinophyta/Heterokontophyta
	353S008229	1	411	YP_003734525	6.72E-72	*Kryptoperidinium foliaceum*	Dinophyta/Heterokontophyta
Photosystem I protein F	353S004412	1	460	YP_003734953	1.58E-61	*Durinskia baltica*	Dinophyta/Heterokontophyta
Photosystem II chlorophyll A core antenna apoprotein CP43	353S020420	1	417	YP_003734530	1.59E-53	*Kryptoperidinium foliaceum*	Dinophyta/Heterokontophyta
Total:	7	14					

Phylogenetic analysis of other genes associated with photosynthetic organisms (hydroxymethylbilane synthase (HMBS) and ascorbate peroxidase) are either poorly resolved as far as placement of the *P. piscicida* (Fig. 2). An isoform of HMBS did not clearly group with any particular lineage (it weakly affiliated with the cryptophyte *Guillardia theta* but without any support) but was distinct from Archaeplastida sequences. In the ascorbate peroxidase phylogeny, *P*. *piscicida* formed a strongly supported clade (100%) with homologues from the non-photosynthetic dinoflagellate *Crypthecodinium cohnii*, which has been revealed from molecular evidence to harbor a relict plastid [23] (Fig. 2).

When the ESTs data were compared with the comprehensive chloroplast protein database of *Arabidopsis thaliana* [33] about 544 contigs (1.86% of total contigs) from *P. piscicida* showed significant homology (E-value <e^−50^) with the chloroplast proteins of *A. thaliana* (Table S2). About 23.5% (162 out of 690 proteins) of plastid-targeted proteins of *A. thaliana* were found in the *P. piscicida* EST dataset, with E-value <e^−50^ suggesting that there are far more photosynthetic genes still remained and expressed in *P. piscicida.*

**Figure 5:**
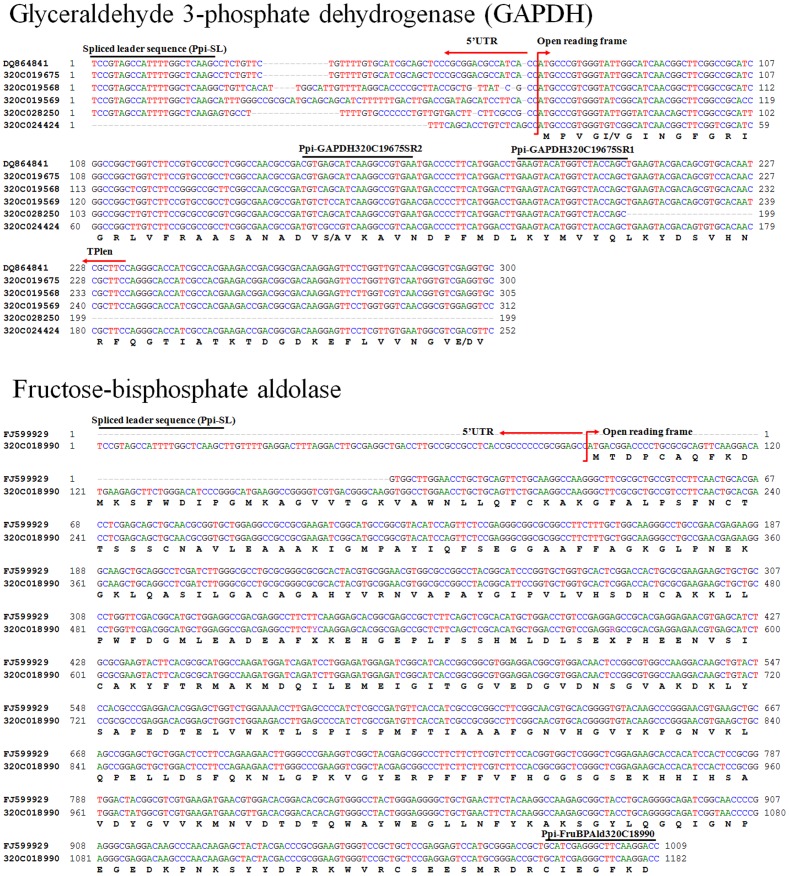
Multiple alignments of 5′ end sequences of GAPDH and fructose-bisphosphate aldolase showing the location of Spliced Leader sequence and specific primer (black lines on the top). 5′ UTR and open reading frame (ORF) were shown with red arrows.

The presence of photosynthetic genes in *P. piscicida* genome was confirmed using 5′-RACE PCR for 15 selected genes. Sub-cellular localization of these genes were shown using CBS prediction program (Table 3). Among them five genes contained Spliced Leader, a signature sequence of *P. piscicida*, at their 5′ ends (Fig. 5).

**Table 3:** List of contigs used for 5′-RACE and prediction of sub-cellular location.

Gene match	Contig (KNU ID)	cTP	mTP	SP	Other	Loc	RC	TPlen	Note
Chloroplast FtsH protease	320C008843	0.044	0.251	0.168	0.74	-	3	-	ESTs
Alpha-amylase	320C010144	0.071	0.146	0.109	0.825	-	2	-	ESTs
Chaperonin	320C019442	0.083	0.298	0.089	0.613	-	4	-	ESTs
Fructose-bisphosphate aldolase	320C018990	0.035	0.033	0.062	0.955	-	1	-	SL
Glyceraldehyde-3-phosphate dehydrogenase	320C019568	0.031	0.616	0.102	0.293	M	4	55	SL
	320C019569	0.031	0.672	0.05	0.38	M	4	55	SL
	320C019675	0.022	0.62	0.093	0.325	M	4	55	SL
	320C028250	0.02	0.761	0.08	0.22	M	3	55	SL
	320C024424	0.014	0.637	0.133	0.297	M	4	55	ESTs
Peptidyl-prolyl isomerase	320C016005	0.03	0.263	0.813	0.015	S	3	18	ESTs
	320C018648	0.113	0.205	0.04	0.813	-	2	-	ESTs
Phosphoglycerate kinase	320C005438	0.056	0.128	0.05	0.775	-	2	-	ESTs
Splicing factor Prp8	320C008393	0.047	0.125	0.142	0.904	-	2	-	ESTs
Pyruvate kinase	320C021827	0.098	0.101	0.149	0.795	-	2	-	ESTs
Triosephosphate isomerase	320C001323	0.334	0.207	0.018	0.4	-	5	-	ESTs
Total:	15								

SL: PCR with Spliced Leader sequence. CBS prediction program was used for analyzing sub-cellular location of each gene.

## Discussion

Our results show that *Pfiesteria piscicida* expresses numerous genes involved in metabolic pathways of plastids despite it not having any sub-cellular membranous structure assignable to plastids [29]. The heterogeneous origins of the plastid genes (especially the genes directly related to photosynthesis) suggest that *P. piscicida* had experienced multiple endosymbioses, both from a secondary plastid (grouping with peridinin-containing dinoflagellate lineages) and at least one tertiary endosymbiosis (grouping with diatoms that have formed endosymbioses with dinoflagellates). This mixed origin of photosynthetic genes has been reported previously for the photosynthetic dinoflagellate *Karlodinium veneficum* (as *K. micrum*) which contains genes both of secondary-endosymbiotic origin and tertiary-endosymbiotic origin, from a haptophyte [15]. Genes for photosynthesis have also been reported in heterotrophic dinoflagellates. The early branching dinoflagellate *Oxyrrhis marina* has several genes associated with plastids but no genes directly involved in the light-reaction or the Calvin-Benson cycle [22]. This is the first report in which many (or a majority with reference to the Calvin-Benson cycle) of the genes involved in photosynthesis have been found in a heterotrophic dinoflagellate. Interestingly genes that are normally located in the plastid (e.g. *psa*A and *psb*A) are found in the transcriptome of *P. piscicida*. Mass transfer of genes from the plastid genome to the nucleus is well documented in dinoflagellates that have a peridinin-containing plastid [41], [42]. Our data would indicate that plastid gene transfer may even occur from a tertiary plastid. While all phylogenetic reconstructions of these ancient endosymbiotic lateral gene transfers need to be interpreted cautiously [10], [16], it is clear that many homologs of both the light reaction and the Calvin-Benson cycle are expressed in this non-photosynthetic organisms.

While the phylogeny of dinoflagellates is not fully resolved [43] due to low support for many clades, *P. piscicida* belongs to the Peridinales (or the Gymnodiniales-Peridinales-Prorocentrales [44]. The Peridinales also contains the “dinotoms”, a group that contains the genera *Kryptoperidinium* and *Durinskia* that harbor a tertiary diatom endosymbionts. These raphe containing diatoms (Bacillariophyceae) are unique to dinoflagellates [45] and our results suggest that *P. piscicida* has also had a previous symbiosis with these diatoms or some common ancestor. The light-reaction genes are often more closely related to the “diatom-plastid” than to other free-living diatoms. This tertiary symbiosis could be an ancestral characteristic of the Peridinales, which has only been retained in a few genera but left its mark in the genome. Future genomics of Peridinales heterotrophic dinoflagellates may elucidate the ancestral nature of this symbiosis.

Genes related to photosynthesis are usually lost in heterotrophic or parasitic organisms, even if the organelle is maintained [46]–[48]. Our results showed that the genes involved in photosynthesis and associated pathways occupy about 0.26% of total ESTs in *P. piscicida.* The presence of a Spliced Leader, a signature sequence of *P. piscicida*, in chosen photosynthetic genes is strong evidence that these genes are indeed encoded and expressed in this species. When we simply compare the EST database with the chloroplast protein database of *Arabidopsis thaliana*, the number becomes 1.86% of total ESTs. This would be a resource costly activity in *P. piscicida* if it had no utility. We suggest several possibilities: One that some photosynthetic genes may be used in some other cellular process. Secondly, the continued expression of plastid genes in *P. piscicida* is non-functional and selection has not removed expression of these genes and this is compensated for by aggressive feeding by *P. piscicida*. Thirdly, *P. piscicida* may get some benefit in being able to maintain these genes to prolong functioning of captured photosynthetic organism or plastid (i.e. kleptoplasty).

Many of the genes matching the *Arabidopsis* proteome database may function in other cellular compartments. Aromatic amino acids produced in the chorismate pathway are produced in the plastids of higher plants but their cellular location in other lineages may be elsewhere [49]. It is also suggested that photosynthetic genes may perform limited carbon fixation [50] while other genes clearly have homology to other non-plastid genes that may be acquired from HGT from bacteria, e.g. EF-Tu [51].

It is also possible that *P. piscicida* may have cryptic plastid still not found. Non-photosynthetic plastids are very difficult to identify. A good example is provided by stramenopiles (or heterokonts) belonging to the clade Dictyochophyceae. Based solely on ultrastructural data, it was postulated that they lost their secondary plastids [7]. However, later studies demonstrated the existence of non-photosynthetic plastids with four envelope membranes and an ER-like outermost membrane connected with the nuclear envelope in these stramenopiles [52]. Recently, Fernández-Robledo et al. [53] reported problems with the identification of a non-photosynthetic plastid in the well-investigated parasite *Perkinsus marinus*. Question still remains in either case; why are these photosynthetic genes still expressed if not for its own functional plastids?

*P. piscicida* is an aggressive predator that could even control other algal blooms [31] and predators on fish [54]. *P. piscicida* feeds with a peduncle (i.e. feeding tube) extracting cell contents from prey into food vacuoles and thus plastids of algal prey could be transferred into the predator's protoplasm without damage [31]. There is a possibility that these acquired plastids work as “kleptoplasts” inside the predator cell. Lewitus et al. [30] reported that plastid of ingested cryptophytes persisted in vacuoles of *P. piscicida* for a week and were apparently fixing small amount of carbon and accumulating starch grains. Additionally, Feinstein et al. [55] showed that the growth rate of *P. piscicida* fed on the cryptophyte *Rhodomonas* sp. at saturating light levels was almost twice as in the darkness. Jeong et al. [31] reported that the gross growth efficiency of *P. piscicida* fed on *Rhodomonas salina* exceeded 100%, which suggests the possibility of kleptoplastidy. However, kleptoplastidic photosynthesis alone is not enough for the survival of *P. piscicida* as the number of *P. piscicida* cells decreased as soon as prey cells were removed and was even faster in the light than in the dark [5], [31], [55]. Recently, Johnson [5] suggested that the enhanced growth of some heterotrophic dinoflagellates including *P. piscicida* may be due to enhanced predation rather than kleptoplastidy. Considering its wide spectrum of prey, it is hard to believe that the numerous plastid genes of *P. piscicida* are used only by its kleptoplastids. Actually, it was initially suggested that unusual dinoflagellate plastids (e.g. the fucoxanthin plastid of *Karenia* and *Karlodinium*) adapted the targeting machinery and hundreds of nucleus-residing plastid genes of the ancestral peridinin plastid. However, the genomic studies by Yoon et al. [11] and Patron et al. [15] questioned the hypothesis. They found that the fucoxanthin plastid uses mainly genes derived from its haptophyte ancestor.

So why does *P. piscicida* cell express so many photosynthetic genes? It is possible that there is reduced selection on the removal and reduction on their expression in these genes. A consequence of this is that heterotrophic dinoflagellates may more easily acquire and maintain symbiotic plastids. The serial replacement of one plastid and another has been seen in the dinotomes (diatom-containing dinoflagellates) [45] and is more prevalent in dinoflagellates than any other group of eukaryotic organisms [56].

Our comprehensive EST data set of the heterotrophic dinoflagellate *P. piscicida* indicated that this organism still expresses a large complement of plastid derived-genes and genes involved with photosynthesis. These genes have mixed phylogenetic histories and indicate the complex nature of predation, symbiosis and plastid loss that is a common feature of dinoflagellates, and may give us insight into how dinoflagellates so readily change plastid throughout their history.

## Supporting Information

Table S1 **Used primer sets for 5′RACE PCR.**
(PDF)

Table S2 **ESTs database of** ***Pfiesteria piscicida*** **contigs associated with the plastid through the chloroplast protein database of** ***Arabidopsis thaliana*** **and their BLAST analysis.**
(PDF)
