# Supplementary material for: Still Acting Green: Continued Expression of Photosynthetic Genes in the Heterotrophic Dinoflagellate Pfiesteria piscicida (Peridiniales, Alveolata)
Source: PLoS One. 2013 Jul 16;8(7):e68232. doi: 10.1371/journal.pone.0068232 (PMC3712967; doi:10.1371/journal.pone.0068232)
Supplement: Table S1 — Used primer sets for 5′RACE PCR. (PDF) [file pone.0068232.s001.pdf]

Supporting table 1

Still Acting Green: Continued Expression of Photosynthetic Genes in the Heterotrophic Dinoflagellate *Pfiesteria piscicida* (Peridiniales, Alveolata)

Gwang Hoon Kim, Hae Jin Jeong, Yeong Du Yoo, Sunju Kim, Ji Hee Han, Jong Won Han, Giuseppe C. Zuccarello

Table S1. Used primer sets for 5’RACE PCR.

Primer name	Sequence
Ppi-SL	TCCGTAGCCATTTTGGCTCAA
Ppi-FruBPAld320C18990SR1	GGTCCTTGAAGCCCTCGAT
Ppi-GAPDH320C19675SR1	GCTGGTAGACCATGTACTTC
Ppi-GAPDH320C19675SR2	TCACGGCCTTGATGCTCACG
