# Supplementary material for: Still Acting Green: Continued Expression of Photosynthetic Genes in the Heterotrophic Dinoflagellate Pfiesteria piscicida (Peridiniales, Alveolata)
Source: PLoS One. 2013 Jul 16;8(7):e68232. doi: 10.1371/journal.pone.0068232 (PMC3712967; doi:10.1371/journal.pone.0068232)
Supplement: Table S2 — ESTs database of Pfiesteria piscicida contigs associated with the plastid through the chloroplast protein database of Arabidopsis thaliana and their BLAST analysis. (PDF) [file pone.0068232.s002.pdf]

Supporting table 2

Still Acting Green: Continued Expression of Photosynthetic Genes in the Heterotrophic Dinoflagellate *Pfiesteria piscicida* (Peridiniales, Alveolata)

Gwang Hoon Kim, Hae Jin Jeong, Yeong Du Yoo, Sunju Kim, Ji Hee Han, Jong Won Han, Giuseppe C. Zuccarello

Table S2. ESTs database of *Pfiesteria piscicida* contigs associated with the plastid through the chloroplast protein database of *Arabidopsis thaliana* and their BLAST analysis.

	Arabidopsis chloroplast gene			*Pfiesteria piscicida* gene						
Category	Description	Acc. No.	E-value	KNU ID	Reads	Sequence description	GenBank Acc. No.	E-value	Origin	Phylum
Calvin cycle	GAPDH	At1g12900	2E-74	320C001994	31	GAPDH	ABI14256	0	*Pfiesteria piscicida*	Dinophyta
		At1g12900	4E-64	320C002033	34	GAPDH	ABI14256	1E-158	*Pfiesteria piscicida*	Dinophyta
		At1g12900	1E-78	320C005475	54	GAPDH	ABI14256	0	*Pfiesteria piscicida*	Dinophyta
		At1g12900	4E-74	320C006750	17	GAPDH	ABI14256	0	*Pfiesteria piscicida*	Dinophyta
		At1g12900	8E-65	320C013130	5	GAPDH	AAM68968	6E-108	*Pyrocystis lunula*	Dinophyta
		At1g12900	5E-79	320C019675	70	GAPDH	ABI14256	0	*Pfiesteria piscicida*	Dinophyta
		At1g12900	1E-79	320C024424	29	GAPDH	ABI14256	0	*Pfiesteria piscicida*	Dinophyta
		At1g12900	9E-54	320C028251	27	GAPDH	ABI14256	4E-126	*Pfiesteria piscicida*	Dinophyta
		At1g42970	5E-70	320C001994	31	GAPDH	ABI14256	0	*Pfiesteria piscicida*	Dinophyta
		At1g42970	7E-59	320C002033	34	GAPDH	ABI14256	1E-158	*Pfiesteria piscicida*	Dinophyta
		At1g42970	1E-74	320C005475	54	GAPDH	ABI14256	0	*Pfiesteria piscicida*	Dinophyta
		At1g42970	1E-69	320C006750	17	GAPDH	ABI14256	0	*Pfiesteria piscicida*	Dinophyta
		At1g42970	2E-66	320C013130	5	GAPDH	AAM68968	6E-108	*Pyrocystis lunula*	Dinophyta
		At1g42970	2E-74	320C019675	70	GAPDH	ABI14256	0	*Pfiesteria piscicida*	Dinophyta
		At1g42970	1E-74	320C024424	29	GAPDH	ABI14256	0	*Pfiesteria piscicida*	Dinophyta
		At1g13440	2E-106	320C001994	31	GAPDH	ABI14256	0	*Pfiesteria piscicida*	Dinophyta
		At1g13440	2E-93	320C002033	34	GAPDH	ABI14256	1E-158	*Pfiesteria piscicida*	Dinophyta

	At1g13440	1E-119	320C005475	54	GAPDH	ABI14256	0	*Pfiesteria piscicida*	Dinophyta
	At1g13440	1E-106	320C006750	17	GAPDH	ABI14256	0	*Pfiesteria piscicida*	Dinophyta
	At1g13440	3E-120	320C019675	70	GAPDH	ABI14256	0	*Pfiesteria piscicida*	Dinophyta
	At1g13440	1E-55	320C021271	26	GAPDH	ABI14256	2E-98	*Pfiesteria piscicida*	Dinophyta
	At1g13440	1E-119	320C024424	29	GAPDH	ABI14256	0	*Pfiesteria piscicida*	Dinophyta
	At1g13440	5E-76	320C028251	27	GAPDH	ABI14256	4E-126	*Pfiesteria piscicida*	Dinophyta
	At1g13440	6E-68	320C029251	39	GAPDH	ABI14256	1E-105	*Pfiesteria piscicida*	Dinophyta
Malate dehydrogenase	At3g47520	2E-60	320C000767	22	Malate dehydrogenase	AAW79318	2E-137	*Heterocapsa triquetra*	Dinophyta
	At3g47520	7E-61	320C001252	35	Malate dehydrogenase	ADV91163	6E-105	*Karlodinium veneficum*	Dinophyta
	At3g47520	2E-67	320C022473	8	Malate glyoxysomal	ADV91165	1E-90	*Karlodinium veneficum*	Dinophyta
	At3g47520	2E-67	320C022473	8	Malate glyoxysomal	ADV91165	1E-90	*Karlodinium veneficum*	Dinophyta
	At5g09660	1E-64	320C000767	22	Malate dehydrogenase	AAW79318	2E-137	*Heterocapsa triquetra*	Dinophyta
	At5g09660	1E-64	320C001252	35	Malate dehydrogenase	ADV91163	6E-105	*Karlodinium veneficum*	Dinophyta
Phosphoglycerate kinase	At1g56190	2E-58	320C003700	12	Phosphoglycerate kinase	AAW79324	4E-76	*Heterocapsa triquetra*	Dinophyta
	At1g56190	2E-58	320C003700	12	Phosphoglycerate kinase	AAW79324	4E-76	*Heterocapsa triquetra*	Dinophyta
	At1g56190	4E-91	320C005438	44	Phosphoglycerate kinase	AAW79324	0	*Heterocapsa triquetra*	Dinophyta
	At3g12780	1E-59	320C003700	12	Phosphoglycerate kinase	AAW79324	4E-76	*Heterocapsa triquetra*	Dinophyta
	At3g12780	1E-59	320C003700	12	Phosphoglycerate kinase	AAW79324	4E-76	*Heterocapsa triquetra*	Dinophyta

		At3g12780	7E-93	320C005438	44	Phosphoglycerate kinase	AAW79324	0	*Heterocapsa triquetra*	Dinophyta
	Pyruvate kinase	At3g22960	4E-64	320C002847	26	Pyruvate	XP_002788069	0	*Perkinsus marinus*	Dinophyta
		At3g22960	1E-62	320C015175	35	Pyruvate	XP_002788069	0	*Perkinsus marinus*	Dinophyta
		At3g22960	1E-62	320C015175	35	Pyruvate	XP_002788069	0	*Perkinsus marinus*	Dinophyta
		At3g22960	3E-51	320C021827	39	Pyruvate	XP_002788069	0	*Perkinsus marinus*	Dinophyta
		At3g22960	8E-57	320C026335	17	Pyruvate	XP_002788065	1E-137	*Perkinsus marinus*	Dinophyta
	Pyruvate phosphate dikinase	At4g15530	1E-70	320C019436	29	Pyruvate phosphate dikinase	YP_006374385	1E-132	*Tistrella mobilis*	Proteobacteria
		At4g15530	3E-55	320C029511	8	Pyruvate phosphate dikinase	YP_002297879	1E-88	*Rhodospirillum centenum*	Proteobacteria
	Ribose 5-phosphate isomerase	At3g04790	1E-51	320C004736	6	Ribose 5-phosphate isomerase	AAW79354	4E-99	*Heterocapsa triquetra*	Dinophyta
		At3g04790	3E-58	320C018635	7	Ribose 5-phosphate isomerase	AAW79354	2E-110	*Heterocapsa triquetra*	Dinophyta
	Transketolase	At2g45290	6E-162	320C008908	29	Transketolase	ABP35605	0	*Karlodinium veneficum*	Dinophyta
		At3g60750	2E-163	320C008908	29	Transketolase	ABP35605	0	*Karlodinium veneficum*	Dinophyta
	Triose-phosphate isomerase	At2g21170	2E-60	320C001323	44	Triose-phosphate isomerase	XP_002768175	3E-90	*Perkinsus marinus*	Dinophyta
Photosynthesis	Psba PSII 32 kDa protein	68258.m00001	1E-155	320C006591	8	Photosystem ii protein D1	YP_003734635	0	*Kryptoperidinium foliaceum*	Dinophyta
Amino acid metabolism	Acetylornithine amino transferase	At1g80600	7E-53	320C001071	10	Acetylornithine aminotransferase	ADV91221	1E-81	*Karlodinium veneficum*	Dinophyta
	Alanine amino-	At1g17290	3E-79	320C009757	15	Alanine aminotransfera	XP_002906651	5E-149	*Phytophthora infestans*	Oomycota

transferase					se				
	At1g23310	8E-106	320C009757	15	Alanine aminotransferase	XP_002906651	5E-149	*Phytophthora infestans*	Oomycota
Aspartate amino transferase	At4g31990	1E-52	320C014744	10	Aspartate partial	XP_002765977	4E-91	*Perkinsus marinus*	Dinophyta
Carbamoyl phosphate synthetase	At1g29900	6E-52	320C002020	18	Carbamoyl phosphate synthetase	XP_003884824	3E-115	*Neospora caninum*	Apicomplexa
	At1g29900	5E-57	320C009900	25	Carbamoyl phosphate synthetase	XP_002784057	4E-170	*Perkinsus marinus*	Dinophyta
	At1g29900	6E-111	320C010203	14	Carbamoyl phosphate synthetase	XP_002784057	0	*Perkinsus marinus*	Dinophyta
	At1g29900	8E-54	320C010203	14	Carbamoyl phosphate synthetase	XP_002784057	0	*Perkinsus marinus*	Dinophyta
	At1g32900	6E-64	320C000390	24	Soluble starch synthase	EKX37680	1E-110	*Guillardia theta*	Cryptophyta
	At1g32900	3E-53	320C001989	14	Soluble starch synthase	XP_004307998	3E-75	*Fragaria vesca*	Streptophyta
	At1g32900	6E-75	320C018048	66	Soluble starch synthase	EKX45880	4E-118	*Guillardia theta*	Cryptophyta
Delta-1-pyrroline-5-carboxylate dehydrogenase	At5g62530	0	320C001088	43	Aldehyde dehydrogenase	EIE18461	0	*Coccomyxa subellipsoidea*	Chlorophyta
	At5g62530	4E-56	320C013513	3	Aldehyde dehydrogenase	XP_001696928	3E-61	*Chlamydomonas reinhardtii*	Chlorophyta
	At5g62530	1E-52	320C026919	4	Aldehyde dehydrogenase	XP_001031541	4E-68	*Tetrahymena thermophila*	Ciliophora
Dihydroxy-acid dehydratase	At3g23940	2E-173	320C015287	42	Dihydroxy-acid dehydratase	XP_002908438	0	*Phytophthora infestans*	Oomycota
Glutamate synthase	At5g04140	4E-51	320C010569	6	Glutamate synthase	XP_001547433	5E-60	*Botryotinia fuckeliana*	Ascomycota

	At5g04140	4E-51	320C010569	6	Glutamate synthase	XP_001547433	5E-60	*Botryotinia fuckeliana*	Ascomycota
	At5g04140	4E-51	320C010569	6	Glutamate synthase	XP_001547433	5E-60	*Botryotinia fuckeliana*	Ascomycota
	At5g53460	2E-113	320C005012	32	Glutamate synthase	BAD95320	9E-129	*Arabidopsis thaliana*	Streptophyta
	At5g53460	2E-52	320C009352	4	Glutamate synthase	XP_002776048	6E-56	*Perkinsus marinus*	Dinophyta
	At5g53460	2E-52	320C009352	4	Glutamate synthase	XP_002776048	6E-56	*Perkinsus marinus*	Dinophyta
	At5g53460	3E-68	320C010569	6	Glutamate synthase	XP_001547433	5E-60	*Botryotinia fuckeliana*	Ascomycota
	At5g53460	3E-68	320C010569	6	Glutamate synthase	XP_001547433	5E-60	*Botryotinia fuckeliana*	Ascomycota
	At5g53460	3E-68	320C010569	6	Glutamate synthase	XP_001547433	5E-60	*Botryotinia fuckeliana*	Ascomycota
	At5g53460	2E-58	320C020231	9	NADH-glutamate synthase small chain	ZP_01093161	1E-58	*Blastopirellula marina*	Planctomycetes
	At5g53460	1E-113	320C020935	25	Glutamate synthase	XP_002986605	4E-119	*Selaginella moellendorffii*	Streptophyta
	At5g53460	1E-113	320C020935	25	Glutamate synthase	XP_002986605	4E-119	*Selaginella moellendorffii*	Streptophyta
	At3g54660	6E-89	320C005971	8	Glutathione reductase	ZP_11130491	1E-109	*Oceanibaculum indicum*	Proteobacteria
	At3g54660	1E-60	320C013620	17	Thioredoxin reductase	XP_002773322	0	*Perkinsus marinus*	Dinophyta
	At3g54660	3E-64	320C029486	151	Thioredoxin reductase	EGD81586	1E-149	*Salpingoeca* sp.	Choanozoa
Glycine dehydrogenase	At2g26080	0	320C003889	65	Glycine dehydrogenase	XP_002785345	0	*Perkinsus marinus*	Dinophyta
Myrosinase	At5g25980	6E-68	320C024129	30	Glycosyl hydrolase	CBJ30694	5E-129	*Ectocarpus siliculosus*	Heterokontophyta
	At5g26000	7E-69	320C024129	30	Glycosyl hydrolase	CBJ30694	5E-129	*Ectocarpus siliculosus*	Heterokontophyta
O-acetylserine (thiol)-lyase	At2g43750	3E-58	320C001087	12	Cysteine synthase	ABV22215	4E-172	*Karlodinium veneficum*	Dinophyta
	At3g59760	7E-59	320C001087	12	Cysteine	ABV22215	4E-172	*Karlodinium*	Dinophyta

		At3g59760	3E-51	320C012870	30	synthase Cystathionine beta-synthase	XP_002678669	8E-133	*veneficum* *Naegleria gruberi*	Percolozoa
Biosynthesis of secondary metabolites	4-hydroxy-3-methylbut-2-enyl diphosphate synthase	At5g60600	2E-56	320C009232	35	4-hydroxy-3-methylbut-2-en-1-yl diphosphate synthase	BAG14389	5E-67	*Perkinsus marinus*	Dinophyta
		At5g60600	7E-90	320C003596	28	4-hydroxy-3-methylbut-2-en-1-yl diphosphate synthase	XP_002506236	4E-110	*Micromonas* sp.	Chlorophyta
	Phytoene dehydrogenase	At4g14210	2E-62	320C026046	4	Phytoene desaturase	XP_002291632	1E-108	*Thalassiosira pseudonana*	Heterokontophyta
	Porphobilinogen synthase	At1g69740	5E-79	320C000026	5	Delta-aminolevulinic acid dehydratase	CAC36151	7E-125	*Lingulodinium polyedrum*	Dinophyta
	Protoporphyrinogen oxidase	At4g01690	6E-56	320C008273	13	Protein	XP_001418278	2E-68	*Ostreococcus lucimarinus*	Chlorophyta
Carbohydrate metabolism	1,4-alpha-glucan branching enzyme	At2g36390	4E-65	320C000244	11	Starch branching enzyme	BAH72207	3E-74	*Acyrthosiphon pisum*	Arthropoda
	Acetyl-coA synthetase	At5g36880	2E-139	320C000320	28	Acetyl-synthetase	EKU22239	0	*Nannochloropsis gaditana*	Heterokontophyta
	Aldehyde dehydrogenase	At3g48000	1E-82	320C002083	40	Aldehyde dehydrogenase	EKU22904	4E-139	*Nannochloropsis gaditana*	Heterokontophyta
		At3g48000	4E-57	320C015448	7	Nad-dependent aldehyde dehydrogenase	YP_007058635	2E-70	*Rivularia* sp.	Cyanobacteria
		At3g48000	2E-68	320C017969	19	Aldehyde dehydrogenase	YP_001239827	1E-125	*Bradyrhizobium* sp.	Proteobacteria
		At4g34240	2E-56	320C020815	52	Aldehyde	CBJ32109	3E-60	*Ectocarpus*	Heterokontophyta

					dehydrogenase			*siliculosus*	
	At4g34240	3E-77	320C021165	21	Nad-dependent aldehyde dehydrogenase	ZP_08427613	2E-112	*Moorea producens*	Cyanobacteria
Beta-glucosidase	At1g26560	1E-59	320C018786	13	Glycoside hydrolase family	YP_004449345	2E-80	*Haliscomeno bacter hydrossis*	Bacteroidetes
	At1g26560	8E-79	320C024129	30	Glycosyl hydrolase family	CBJ30694	5E-129	*Ectocarpus siliculosus*	Heterokontophyta
Dihydrolipoamide dehydrogenase	At3g17240	3E-105	320C001171	28	Dihydrolipoyl dehydrogenase	XP_003201968	1E-134	*Meleagris gallopavo*	Chordata
	At3g17240	2E-102	320C007331	27	Dihydrolipoyl dehydrogenase	NP_001080894	8E-136	*Xenopus laevis*	Chordata
Fructose-1,6-bisphosphatase	At3g54050	4E-68	320C011913	13	Fructose1,6-bisphosphatase	EGB05300	3E-116	*Aureococcus anophagefferens*	Heterokontophyta
	At3g54050	4E-59	320C012917	10	Fructose1,6 -bisphosphatase	EGB05300	1E-108	*Aureococcus anophagefferens*	Heterokontophyta
Fructose-bisphosphate aldolase	At3g52930	6E-56	320C025487	4	Fructose-bisphosphate cytoplasmic isozyme-like	NP_001242086	6E-54	*Glycine max*	Streptophyta
	At3g52930	6E-56	320C025487	4	Fructose-bisphosphate cytoplasmic isozyme-like	NP_001242086	6E-54	*Glycine max*	Streptophyta
	At4g26530	2E-53	320C025487	4	Fructose-bisphosphate cytoplasmic isozyme-like	NP_001242086	6E-54	*Glycine max*	Streptophyta
	At4g26530	2E-53	320C025487	4	Fructose-bisphosphate cytoplasmic isozyme-like	NP_001242086	6E-54	*Glycine max*	Streptophyta
Glucan phosphorylase	At3g29320	5E-74	320C022875	8	Glycogen phosphorylase	XP_002364313	3E-108	*Toxoplasma gondii*	Apicomplexa

	At3g29320	2E-83	320C023472	24	Glycogen phosphorylase	XP_002364313	1E-132	*Toxoplasma gondii*	Apicomplexa
	At3g29320	1E-65	320C023473	11	Glycogen phosphorylase	XP_003885075	6E-91	*Neospora caninum*	Apicomplexa
	At3g29320	1E-65	320C023473	11	Glycogen phosphorylase	XP_003885075	6E-91	*Neospora caninum*	Apicomplexa
Glycine hydroxymethy ltransferase	At4g37930	7E-101	320C002543	24	Serine hydroxymethy l transferase	XP_002505413	1E-138	*Micromonas* sp.	Chlorophyta
	At4g37930	3E-135	320C008259	27	Serine hydroxymethy l transferase	XP_002946621	3E-167	*Volvox carteri*	Chlorophyta
	At4g37930	3E-135	320C008259	27	Serine hydroxymethy l transferase	XP_002946621	3E-167	*Volvox carteri*	Chlorophyta
	At4g37930	1E-96	320C008961	11	Serine hydroxymethy l transferase	XP_003059685	7E-135	*Micromonas pusilla*	Chlorophyta
	At4g37930	2E-60	320C018556	21	Serine hydroxymethy l transferase	CBK21642	2E-94	*Blastocystis hominis*	-
	At4g37930	6E-90	320C024774	11	Serine hydroxymethy l transferase	XP_002946621	1E-106	*Volvox carteri*	Chlorophyta
	At4g37930	6E-90	320C024774	11	Serine hydroxymethy l transferase	XP_002946621	1E-106	*Volvox carteri*	Chlorophyta
	At4g37930	8E-100	320C028527	21	Serine hydroxymethy l transferase	XP_003884541	2E-145	*Neospora caninum*	Apicomplexa
Glycolate oxidase	At3g14420	1E-77	320C014179	18	Cytochrome	GAA94888	1E-160	*Mixia osmundae IAM*	Basidiomycota
Phospho glucomutase	At1g23190	4E-167	320C016157	27	Phospho glucomutase	EKX45869	0	*Guillardia theta*	Cryptophyta
	At1g23190	7E-109	320C017610	38	Phospho glucomutase	EGZ27518	7E-145	*Phytophthora sojae*	Oomycota

		At1g23190	2E-70	320C020057	33	Phospho glucomutase	CCA18915	2E-90	*Albugo laibachii*	Oomycota
		At1g23190	2E-104	320C020731	23	phospho glucomutase	EKX47841	2E-127	*Guillardia theta*	Cryptophyta
		At5g51820	7E-158	320C016157	27	Phospho glucomutase	EKX45869	0	*Guillardia theta*	Cryptophyta
		At5g51820	1E-99	320C017610	38	Phospho glucomutase	EGZ27518	7E-145	*Phytophthora sojae*	Oomycota
		At5g51820	1E-58	320C020057	33	Phospho glucomutase	CCA18915	2E-90	*Albugo laibachii*	Oomycota
		At5g51820	2E-94	320C020731	23	Phospho glucomutase	EKX47841	2E-127	*Guillardia theta*	Cryptophyta
Energy metaboli sm	ATP synthase beta	At5g08670	1E-58	320C003837	2	ATP synthase subunit beta	ZP_01907032	6E-87	*Plesiocystis pacifica*	Proteobacteria
		At5g08670	9E-87	320C013463	14	H+ transporting ATP synthase beta subunit	ACU45001	5E-111	*Pfiesteria piscicida*	Dinophyta
		At5g08670	2E-85	320C013828	7	ATP H+ transporting f1 complex beta subunit	ABD77232	1E-114	*Tamandua tetradactyla*	Chordata
		At5g08670	5E-72	320C013922	6	ATP synthase beta subunit	CCA23253	1E-90	*Albugo laibachii*	Oomycota
		At5g08670	1E-134	320C019667	7	ATP synthase beta subunit	ACU45001	0	*Pfiesteria piscicida*	Dinophyta
		At5g08670	8E-60	320C028395	24	ATP synthase subunit partial	ACU45001	8E-75	*Pfiesteria piscicida*	Dinophyta
		At5g08670	1E-56	320C029274	13	ATP H+ transporting f1 complex beta subunit	ADV91189	3E-78	*Karlodinium veneficum*	Dinophyta
		At5g08670	2E-82	320C029923	27	ATP synthase beta	ACU45001	2E-115	*Pfiesteria piscicida*	Dinophyta
		At5g08670	3E-54	320C030094	8	ATP synthase subunit partial	ACU45001	1E-67	*Pfiesteria piscicida*	Dinophyta
	Inorganic pyro phosphatase	At1g15690	2E-63	320C000140	53	H+-translocating pyrophosphata	XP_002287300	2E-83	*Thalassiosira pseudonana*	Heterokontophyta

					se family				
	At1g15690	2E-66	320C009610	9	Pyrophosphate-energized membrane proton pump 2	BAJ33614	4E-107	*Thellungiella halophila*	Streptophyta
	At1g15690	6E-64	320C010609	36	H+-translocating pyrophosphatase family	XP_002287300	3E-87	*Thalassiosira pseudonana*	Heterokontophyta
	At1g15690	1E-173	320C012232	41	H+-translocating pyrophosphatase family	EGB06142	0	*Aureococcus anophagefferens*	Heterokontophyta
	At1g15690	2E-82	320C019183	47	H+-translocating pyrophosphatase family	EJK49356	1E-112	*Thalassiosira oceanica*	Heterokontophyta
	At1g15690	3E-113	320C019709	184	Pyrophosphate-energized vacuolar membrane proton pump 1-like	EGB06142	2E-153	*Aureococcus anophagefferens*	Heterokontophyta
	At1g15690	6E-64	320C024569	28	Inorganic pyrophosphatase	XP_002287300	2E-88	*Thalassiosira pseudonana*	Heterokontophyta
	At1g15690	1E-103	320C026714	32	H+-translocating pyrophosphatase family	CBN78820	3E-143	*Ectocarpus siliculosus*	Heterokontophyta
V-type ATP synthase subunit B	At1g20260	2E-75	320C000442	32	Vacuolar ATP synthase subunit	XP_002782779	0	*Perkinsus marinus*	Dinophyta
	At1g20260	4E-97	320C020506	23	Vacuolar ATP synthase subunit	XP_002785337	0	*Perkinsus marinus*	Dinophyta

		At1g20260	1E-81	320C020974	13	Vacuolar ATP synthase subunit	XP_002782779	1E-131	*Perkinsus marinus*	Dinophyta
		At1g20260	2E-55	320C023711	18	Vacuolar ATP synthase subunit	ACF28659	1E-83	*Amphidinium carterae*	Dinophyta
		At1g20260	1E-87	320C029813	16	Vacuolar ATP synthase subunit	XP_002772672	0	*Perkinsus marinus*	Dinophyta
		At1g76030	4E-161	320C000442	32	Vacuolar ATP synthase subunit	XP_002782779	0	*Perkinsus marinus*	Dinophyta
		At1g76030	0	320C020506	23	Vacuolar ATP synthase subunit	XP_002785337	0	*Perkinsus marinus*	Dinophyta
		At1g76030	1E-89	320C020974	13	Vacuolar ATP synthase subunit	XP_002782779	1E-131	*Perkinsus marinus*	Dinophyta
		At1g76030	1E-55	320C023711	18	Vacuolar ATP synthase subunit	ACF28659	1E-83	*Amphidinium carterae*	Dinophyta
		At1g76030	2E-173	320C029813	16	Vacuolar ATP synthase subunit	XP_002772672	0	*Perkinsus marinus*	Dinophyta
Lipid metabolism	Abnormal inflorescence meristem	At4g29010	1E-100	320C000181	46	3-hydroxyacyl-dehydrogenase	XP_002776796	0	*Perkinsus marinus*	Dinophyta
		At4g29010	2E-110	320C018935	241	3-hydroxyacyl-dehydrogenase	XP_002776796	0	*Perkinsus marinus*	Dinophyta
		At4g29010	3E-108	320C019832	30	3-hydroxyacyl-dehydrogenase	YP_004552309	0	*Sphingobium chlorophenolicum*	Proteobacteria
		At4g29010	2E-56	320C021278	43	3-hydroxyacyl-dehydrogenase	XP_002776796	5E-144	*Perkinsus marinus*	Dinophyta
	Acetyl-coA C-acyltransferase	At2g33150	1E-92	320C000108	31	3-ketoacyl-thiolase peroxisomal	XP_002780408	5E-123	*Perkinsus marinus*	Dinophyta

		At2g33150	6E-65	320C000959	34	Acetyl-acetyltransferase	ZP_05061846	1E-153	*gamma proteo bacterium*	Proteobacteria
		At2g33150	8E-93	320C017346	22	3-ketoacyl-thiolase peroxisomal	XP_002780408	9E-133	*Perkinsus marinus*	Dinophyta
		At2g33150	2E-59	320C019203	29	3-ketoacyl-thiolase peroxisomal	XP_002780408	1E-76	*Perkinsus marinus*	Dinophyta
		At2g33150	4E-73	320C020504	22	3-ketoacyl-thiolase peroxisomal	XP_002780408	5E-113	*Perkinsus marinus*	Dinophyta
		At2g33150	7E-69	320C022255	21	3-ketoacyl-thiolase peroxisomal	EGW00823	2E-97	*Cricetulus griseus*	Chordata
		At2g33150	8E-51	320C028443	24	3-ketoacyl-peroxisomal	XP_004315495	2E-55	*Tursiops truncatus*	Chordata
		At2g33150	8E-51	320C028443	24	3-ketoacyl-peroxisomal	XP_004315495	2E-55	*Tursiops truncatus*	Chordata
		At2g33150	5E-80	320C028714	38	3-ketoacyl-thiolase peroxisomal	XP_002780408	4E-113	*Perkinsus marinus*	Dinophyta
	Enoyl-[acyl-carrier protein] reductase	At2g05990	2E-66	320C019240	4	Enoyl-acp reductase	CBN77155	4E-81	*Ectocarpus siliculosus*	Heterokontophyta
Nucleotide metabolism	Phosphoribosyl glycinamide synthetase	At1g09830	1E-94	320C000238	16	Phosphoribosylamine--glycine ligase	XP_002900903	3E-145	*Phytophthora infestans*	Oomycota
		At1g74260	6E-176	320C001761	18	Phosphoribosylformylglycinamidine synthase	XP_002524208	0	*Ricinus communis*	Streptophyta
		At1g74260	6E-119	320C001995	18	Phosphoribosylformylglycinamidine	NP_001045039	8E-125	*Oryza sativa*	Streptophyta

						synthase				
Protein processing	Alpha 1,3-glucosidase	At5g63840	1E-159	320C003834	32	Neutral alpha-glucosidase ab	NP_001169712	8E-179	*Zea mays*	Streptophyta
	Calnexin	At5g07340	5E-109	320C010905	37	Calnexin	EOA89024	7E-125	*Setosphaeria turcica*	Ascomycota
	Calreticulin	At1g56340	4E-68	320C001514	27	Calreticulin precursor	XP_002291609	5E-62	*Thalassiosira pseudonana*	Heterokontophyta
		At1g56340	1E-74	320C005199	8	Calreticulin	EGB11415	7E-78	*Aureococcus anophagefferens*	Heterokontophyta
		At1g56340	2E-62	320C010905	37	Calnexin	EOA89024	7E-125	*Setosphaeria turcica*	Ascomycota
		At1g56340	8E-93	320C020012	14	Calreticulin	ABI74618	3E-106	*Eisenia andrei*	Annelida
		At1g56340	4E-54	320C024559	8	At1g09210 t12m4_8	AFK45812	4E-53	*Lotus japonicus*	Streptophyta
		At1g56340	4E-54	320C024559	8	At1g09210 t12m4_8	AFK45812	4E-53	*Lotus japonicus*	Streptophyta
	GTP-binding protein SAR1	At1g09180	5E-67	320C000884	21	Small gtp-binding protein	XP_667389	7E-104	*Cryptosporidium hominis*	Apicomplexa
	Luminal binding protein	At5g28540	3E-158	320C000044	85	Chaperone	ZP_01906286	0	*Plesiocystis pacifica*	Proteobacteria
		At5g28540	7E-60	320C000266	3	Chaperone	ZP_05785977	1E-110	*Silicibacter lacuscaerulensis*	Proteobacteria
		At5g28540	0	320C000309	382	Heat shock protein	XP_002780415	0	*Perkinsus marinus*	Dinophyta
		At5g28540	9E-157	320C000520	42	Heat shock protein 70	P41753	0	*Achlya klebsiana*	Oomycota
		At5g28540	0	320C000629	38	Heat shock protein	AAM02971	0	*Crypthecodinium cohnii*	Dinophyta
		At5g28540	7E-121	320C000813	48	Heat shock protein 70kd	XP_001713581	1E-149	*Guillardia theta*	Cryptophyta
		At5g28540	0	320C000905	43	Luminal-binding protein 5-like	CBJ48460	0	*Ectocarpus siliculosus*	Heterokontophyta
		At5g28540	2E-167	320C001247	81	Chaperone protein	XP_002765356	0	*Perkinsus marinus*	Dinophyta
		At5g28540	8E-180	320C001316	36	Heat shock protein chaperone	CBJ48460	0	*Ectocarpus siliculosus*	Heterokontophyta

At5g28540	8E-65	320C001896	17	Heat shock protein	AAM02973	3E-106	*Crypthecodinium cohnii*	Dinophyta
At5g28540	8E-65	320C001896	17	Heat shock protein	AAM02973	3E-106	*Crypthecodinium cohnii*	Dinophyta
At5g28540	4E-69	320C001992	14	Heat shock protein	AAM02973	9E-124	*Crypthecodinium cohnii*	Dinophyta
At5g28540	0	320C002021	81	Heat shock protein 70	AAR21576	0	*Phytophthora nicotianae*	Oomycota
At5g28540	0	320C002105	41	Heat shock protein	AAM02973	0	*Crypthecodinium cohnii*	Dinophyta
At5g28540	2E-62	320C002126	4	Heat shock protein 70	EJK51576	1E-80	*Thalassiosira oceanica*	Heterokontophyta
At5g28540	2E-62	320C002126	4	Heat shock protein 70	EJK51576	1E-80	*Thalassiosira oceanica*	Heterokontophyta
At5g28540	4E-54	320C003591	3	Heat shock protein partial	AAW58103	2E-71	*Heterocapsa triquetra*	Dinophyta
At5g28540	2E-162	320C007085	36	Heat shock protein	AAM02973	0	*Crypthecodinium cohnii*	Dinophyta
At5g28540	2E-112	320C007130	24	Heat shock protein	ACU45196	0	*Prorocentrum minimum*	Dinophyta
At5g28540	9E-56	320C009212	13	Heat shock protein	AET50612	7E-60	*Eimeria tenella*	Apicomplexa
At5g28540	2E-57	320C011902	23	Hsp70-like protein	CBJ30106	9E-79	*Ectocarpus siliculosus*	Heterokontophyta
At5g28540	0	320C013994	38	Heat shock protein	AAM02973	0	*Crypthecodinium cohnii*	Dinophyta
At5g28540	1E-68	320C016014	23	Heat shock protein	AAM02971	3E-101	*Crypthecodinium cohnii*	Dinophyta
At5g28540	0	320C016915	45	Heat shock protein	AAM02973	0	*Crypthecodinium cohnii*	Dinophyta
At5g28540	1E-50	320C018558	16	Heat shock protein	ABA28988	2E-79	*Symbiodinium* sp.	Dinophyta
At5g28540	1E-58	320C018640	36	Heat shock protein	AAM02973	1E-87	*Crypthecodinium cohnii*	Dinophyta
At5g28540	1E-58	320C018640	36	Heat shock protein	AAM02973	1E-87	*Crypthecodinium cohnii*	Dinophyta

At5g28540	2E-63	320C018823	23	Heat shock protein	ABA28988	3E-92	*Symbiodinium* sp.	Dinophyta
At5g28540	8E-53	320C018859	18	Heat shock protein 70	AAR21576	1E-82	*Phytophthora nicotianae*	Oomycota
At5g28540	7E-86	320C019201	23	Heat shock protein	AAM02971	2E-102	*Crypthecodinium cohnii*	Dinophyta
At5g28540	7E-86	320C019201	23	Heat shock protein	AAM02971	2E-102	*Crypthecodinium cohnii*	Dinophyta
At5g28540	2E-162	320C019758	59	Chaperone	XP_002765356	0	*Perkinsus marinus*	Dinophyta
At5g28540	2E-162	320C019758	59	Chaperone	XP_002765356	0	*Perkinsus marinus*	Dinophyta
At5g28540	5E-82	320C019869	20	Heat shock protein 70	AAM02971	9E-122	*Crypthecodinium cohnii*	Dinophyta
At5g28540	9E-52	320C020021	3	Chaperone	YP_007706640	7E-94	*Octadecabacter antarcticus*	Proteobacteria
At5g28540	9E-52	320C020021	3	Chaperone	YP_007706640	7E-94	*Octadecabacter antarcticus*	Proteobacteria
At5g28540	0	320C020757	82	Heat shock protein 70	AAM02971	0	*Crypthecodinium cohnii*	Dinophyta
At5g28540	0	320C022171	36	Heat shock protein	AAM02973	0	*Crypthecodinium cohnii*	Dinophyta
At5g28540	9E-106	320C022502	17	Heat shock protein	XP_002780414	2E-149	*Perkinsus marinus*	Dinophyta
At5g28540	8E-68	320C022503	5	Heat shock protein	XP_002780414	8E-101	*Perkinsus marinus*	Dinophyta
At5g28540	1E-82	320C022707	17	Heat shock protein hsp70 family protein	AER57864	5E-102	*Acytostelium subglobosum*	Amoebozoa
At5g28540	0	320C022780	21	Heat shock protein	AAM02971	0	*Crypthecodinium cohnii*	Dinophyta
At5g28540	0	320C023326	134	Heat shock protein	AAM02973	0	*Crypthecodinium cohnii*	Dinophyta
At5g28540	1E-56	320C024364	8	Heat shock 70 kDa precursor	XP_002898043	3E-121	*Phytophthora infestans*	Oomycota
At5g28540	1E-56	320C024364	8	Heat shock 70 kDa precursor	XP_002898043	3E-121	*Phytophthora infestans*	Oomycota
At5g28540	9E-68	320C024888	10	Heat shock 70 kDa protein 6	AAM02971	4E-68	*Crypthecodinium cohnii*	Dinophyta

		At5g28540	9E-68	320C024888	10	Heat shock 70 kDa protein 6	AAM02971	4E-68	*Crypthecodinium cohnii*	Dinophyta
		At5g28540	2E-59	320C025398	6	Heat shock protein	CCD13252	2E-87	*Trypanosoma congolense*	Euglenozoa
		At5g28540	2E-70	320C025399	18	Heat shock protein 70 kDa	ACU17965	1E-75	*Glycine max*	Streptophyta
		At5g28540	7E-53	320C026486	8	Stress-70	BAE73040	1E-80	*Macaca fascicularis*	Chordata
		At5g28540	7E-53	320C026486	8	Stress-70	BAE73040	1E-80	*Macaca fascicularis*	Chordata
		At5g28540	3E-74	320C027313	22	Heat shock protein	AAM02971	7E-94	*Crypthecodinium cohnii*	Dinophyta
		At5g28540	3E-59	320C029055	17	Heat shock protein 70 kDa	ACU17965	2E-67	*Glycine max*	Streptophyta
		At5g28540	2E-100	320C030359	23	Heat shock protein	AAM02971	2E-150	*Crypthecodinium cohnii*	Dinophyta
Other	14-3-3 protein GF14 chi	At4g09000	5E-80	320C001128	17	14-3-3 protein	ABI14275	3E-140	*Pfiesteria piscicida*	Dinophyta
		At4g09000	2E-70	320C001131	25	14-3-3 protein	ABI14275	3E-122	*Pfiesteria piscicida*	Dinophyta
		At4g09000	3E-81	320C001275	30	14-3-3 protein	ACU45066	3E-143	*Pfiesteria piscicida*	Dinophyta
		At4g09000	1E-80	320C004089	19	14-3-3 protein	ACU45066	7E-147	*Pfiesteria piscicida*	Dinophyta
		At4g09000	1E-72	320C005718	20	14-3-3 protein	EIE86998	1E-98	*Rhizopus delemar*	Zygomycota
		At4g09000	4E-81	320C009339	20	14-3-3 protein	ACU45066	3E-141	*Pfiesteria piscicida*	Dinophyta
		At4g09000	6E-80	320C015387	14	14-3-3 protein	CCF54339	9E-83	*Ustilago hordei*	Basidiomycota
		At4g09000	8E-56	320C020266	15	14-3-3 protein	ACU45066	8E-89	*Pfiesteria piscicida*	Dinophyta
		At4g09000	8E-56	320C020266	15	14-3-3 protein	ACU45066	8E-89	*Pfiesteria piscicida*	Dinophyta
		At4g09000	5E-71	320C020267	16	14-3-3 protein	ABI14275	4E-116	*Pfiesteria piscicida*	Dinophyta
		At4g09000	8E-55	320C030053	42	14-3-3 protein	ABI14275	2E-91	*Pfiesteria piscicida*	Dinophyta

14-3-3 protein GF14 kappa	At5g65430	5E-78	320C001128	17	14-3-3 protein	ABI14275	3E-140	*Pfiesteria piscicida*	Dinophyta
	At5g65430	4E-68	320C001131	25	14-3-3 protein	ABI14275	3E-122	*Pfiesteria piscicida*	Dinophyta
	At5g65430	6E-79	320C001275	30	14-3-3 protein	ACU45066	3E-143	*Pfiesteria piscicida*	Dinophyta
	At5g65430	1E-78	320C004089	19	14-3-3 protein	ACU45066	7E-147	*Pfiesteria piscicida*	Dinophyta
	At5g65430	2E-71	320C005718	20	14-3-3 protein	EIE86998	1E-98	*Rhizopus delemar*	Zygomycota
	At5g65430	3E-78	320C009339	20	14-3-3 protein	ACU45066	3E-141	*Pfiesteria piscicida*	Dinophyta
	At5g65430	6E-78	320C015387	14	14-3-3 protein	CCF54339	9E-83	*Ustilago hordei*	Basidiomycota
	At5g65430	2E-51	320C020266	15	14-3-3 protein	ACU45066	8E-89	*Pfiesteria piscicida*	Dinophyta
	At5g65430	1E-67	320C020267	16	14-3-3 protein	ABI14275	4E-116	*Pfiesteria piscicida*	Dinophyta
	At5g65430	6E-52	320C030053	42	14-3-3 protein	ABI14275	2E-91	*Pfiesteria piscicida*	Dinophyta
14-3-3 protein GF14 lambda	At5g10450	8E-78	320C001128	17	14-3-3 protein	ABI14275	3E-140	*Pfiesteria piscicida*	Dinophyta
	At5g10450	8E-69	320C001131	25	14-3-3 protein	ABI14275	3E-122	*Pfiesteria piscicida*	Dinophyta
	At5g10450	3E-78	320C001275	30	14-3-3 protein	ACU45066	3E-143	*Pfiesteria piscicida*	Dinophyta
	At5g10450	5E-79	320C004089	19	14-3-3 protein	ACU45066	7E-147	*Pfiesteria piscicida*	Dinophyta
	At5g10450	7E-69	320C005718	20	14-3-3 protein	EIE86998	1E-98	*Rhizopus delemar*	Zygomycota
	At5g10450	2E-77	320C009339	20	14-3-3 protein	ACU45066	3E-141	*Pfiesteria piscicida*	Dinophyta
	At5g10450	6E-77	320C015387	14	14-3-3 protein	CCF54339	9E-83	*Ustilago hordei*	Basidiomycota
	At5g10450	5E-52	320C020266	15	14-3-3 protein	ACU45066	8E-89	*Pfiesteria piscicida*	Dinophyta
	At5g10450	4E-68	320C020267	16	14-3-3 protein	ABI14275	4E-116	*Pfiesteria piscicida*	Dinophyta
	At5g10450	8E-52	320C030053	42	14-3-3 protein	ABI14275	2E-91	*Pfiesteria piscicida*	Dinophyta
14-3-3 protein GF14 nu	At3g02520	1E-78	320C001128	17	14-3-3 protein	ABI14275	3E-140	*Pfiesteria piscicida*	Dinophyta
	At3g02520	8E-69	320C001131	25	14-3-3 protein	ABI14275	3E-122	*Pfiesteria*	Dinophyta

								piscicida	
	At3g02520	5E-80	320C001275	30	14-3-3 protein	ACU45066	3E-143	*Pfiesteria piscicida*	Dinophyta
	At3g02520	5E-79	320C004089	19	14-3-3 protein	ACU45066	7E-147	*Pfiesteria piscicida*	Dinophyta
	At3g02520	4E-72	320C005718	20	14-3-3 protein	EIE86998	1E-98	*Rhizopus delemar*	Zygomycota
	At3g02520	1E-79	320C009339	20	14-3-3 protein	ACU45066	3E-141	*Pfiesteria piscicida*	Dinophyta
	At3g02520	2E-79	320C015387	14	14-3-3 protein	CCF54339	9E-83	*Ustilago hordei*	Basidiomycota
	At3g02520	4E-55	320C020266	15	14-3-3 protein	ACU45066	8E-89	*Pfiesteria piscicida*	Dinophyta
	At3g02520	4E-55	320C020266	15	14-3-3 protein	ACU45066	8E-89	*Pfiesteria piscicida*	Dinophyta
	At3g02520	9E-70	320C020267	16	14-3-3 protein	ABI14275	4E-116	*Pfiesteria piscicida*	Dinophyta
	At3g02520	3E-54	320C030053	42	14-3-3 protein	ABI14275	2E-91	*Pfiesteria piscicida*	Dinophyta
Actin	At1g49240	1E-96	320C000330	52	Actin	ABV00885	2E-143	*Pfiesteria piscicida*	Dinophyta
	At1g49240	2E-114	320C001352	24	Alpha-centractin	XP_003886078	2E-145	*Neospora caninum*	Apicomplexa
	At1g49240	2E-114	320C001352	24	Alpha-centractin	XP_003886078	2E-145	*Neospora caninum*	Apicomplexa
	At1g49240	8E-128	320C001644	17	Actin	ACM78541	0	*Thraustochytrium aureum*	Labyrinthulomycota
	At1g49240	5E-55	320C003134	6	Actin-related protein	XP_784353	1E-100	*Strongylocentrotus purpuratus*	Echinodermata
	At1g49240	1E-81	320C004361	17	Actin	BAF91213	5E-108	*Cyanidium caldarium*	Rhodophyta
	At1g49240	8E-66	320C004377	22	Actin	EJY83159	3E-79	*Oxytricha trifallax*	Ciliophora
	At1g49240	2E-115	320C007325	13	Actin	AFX83578	3E-159	*Pentapharsodinium dalei*	Dinophyta
	At1g49240	2E-114	320C013120	12	Actin	ABV00885	1E-163	*Pfiesteria piscicida*	Dinophyta
	At1g49240	1E-81	320C016762	12	Actin	AFX83576	3E-83	*Gymnodinium*	Dinophyta

								aureolum	
	At1g49240	1E-81	320C016762	12	Actin	AFX83576	3E-83	*Gymnodinium aureolum*	Dinophyta
	At1g49240	0	320C017718	88	Actin	AAQ55798	0	*Vannella ebro*	Amoebozoa
	At1g49240	0	320C017718	88	Actin	AAQ55798	0	*Vannella ebro*	Amoebozoa
	At1g49240	5E-149	320C019955	23	Actin	EGB05933	0	*Aureococcus anophagefferens*	Heterokontophyta
	At1g49240	5E-149	320C019955	23	Actin	EGB05933	0	*Aureococcus anophagefferens*	Heterokontophyta
	At1g49240	5E-113	320C020399	18	Actin	AFX83578	1E-128	*Pentapharsodiniu m dalei*	Dinophyta
	At1g49240	5E-113	320C020399	18	Actin	AFX83578	1E-128	*Pentapharsodiniu m dalei*	Dinophyta
	At1g49240	1E-74	320C020523	11	Actin	AEC48652	1E-101	*Cochliopodium larifeili*	Amoebozoa
	At1g49240	8E-60	320C022723	84	Actin	AFX83578	4E-35	*Pentapharsodiniu m dalei*	Dinophyta
	At1g49240	8E-60	320C022723	84	Actin	AFX83578	4E-35	*Pentapharsodiniu m dalei*	Dinophyta
	At1g49240	2E-72	320C025694	21	Actin	ADU24746	7E-106	*Dinophysis caudata*	Dinophyta
	At1g49240	7E-109	320C027696	12	Actin	ABV00885	9E-141	*Pfiesteria piscicida*	Dinophyta
	At1g49240	7E-109	320C027696	12	Actin	ABV00885	9E-141	*Pfiesteria piscicida*	Dinophyta
	At1g49240	2E-81	320C028380	24	Actin	AFX83578	2E-112	*Pentapharsodiniu m dalei*	Dinophyta
Alpha tubulin	At1g04820	3E-54	320C000206	20	Beta tubulin	AFX83590	1E-139	*Scrippsiella trochoidea*	Dinophyta
	At1g04820	0	320C000325	40	Alpha tubulin chain	AFD34242	0	*Prorocentrum minimum*	Dinophyta
	At1g04820	3E-154	320C000621	89	Alpha tubulin	ABV72532	0	*Heterocapsa triquetra*	Dinophyta
	At1g04820	0	320C001258	53	Alpha tubulin chain	ABV72532	0	*Heterocapsa triquetra*	Dinophyta
	At1g04820	0	320C001846	54	Alpha tubulin chain	ABV22199	0	*Karlodinium veneficum*	Dinophyta
	At1g04820	6E-68	320C002005	27	Alpha tubulin	XP_002783368	1E-85	*Perkinsus marinus*	Dinophyta

	At1g04820	1E-64	320C002623	56	Beta tubulin	AAO49334	0	*Amphidinium corpulentum*	Dinophyta
	At1g04820	9E-129	320C005291	62	Alpha tubulin chain	ABV22199	5E-133	*Karlodinium veneficum*	Dinophyta
	At1g04820	9E-129	320C005291	62	Alpha tubulin chain	ABV22199	5E-133	*Karlodinium veneficum*	Dinophyta
	At1g04820	3E-84	320C009187	21	Beta tubulin	AFX83586	0	*Gymnodinium aureolum*	Dinophyta
	At1g04820	8E-66	320C010828	18	Beta tubulin	AFX83584	5E-173	*Alexandrium minutum*	Dinophyta
	At1g04820	3E-53	320C015009	7	Beta tubulin	ABR22560	1E-125	*Karenia brevis*	Dinophyta
	At1g04820	0	320C018074	51	Alpha tubulin chain	ABV72532	0	*Heterocapsa triquetra*	Dinophyta
	At1g04820	2E-109	320C018193	30	Alpha tubulin	ABJ80978	7E-145	*Peridinium limbatum*	Dinophyta
	At1g04820	0	320C018199	70	Alpha tubulin	AFD34242	0	*Prorocentrum minimum*	Dinophyta
	At1g04820	3E-96	320C019514	36	Beta- partial	AFX83586	0	*Gymnodinium aureolum*	Dinophyta
	At1g04820	1E-126	320C019851	17	Alpha tubulin	ABJ80978	2E-168	*Peridinium limbatum*	Dinophyta
	At1g04820	6E-113	320C019853	22	Alpha tubulin	ABJ80978	9E-150	*Peridinium limbatum*	Dinophyta
	At1g04820	6E-113	320C019854	48	Alpha tubulin	ABJ80978	9E-150	*Peridinium limbatum*	Dinophyta
	At1g04820	4E-98	320C020372	19	Beta tubulin	AAM02970	0	*Crypthecodinium cohnii*	Dinophyta
	At1g04820	3E-62	320C021833	14	Beta tubulin	AFX83582	7E-162	*Adenoides eludens*	Dinophyta
	At1g04820	2E-116	320C022013	20	Alpha tubulin	AAT09064	5E-129	*Bigelowiella natans*	Cercozoa
	At1g04820	0	320C023001	90	Alpha tubulin	ABV72532	0	*Heterocapsa triquetra*	Dinophyta
	At1g04820	2E-60	320C023598	30	Beta tubulin	AAM02970	0	*Crypthecodinium cohnii*	Dinophyta
	At1g04820	5E-58	320C026773	25	Beta tubulin	AAO49334	2E-139	*Amphidinium*	Dinophyta

								corpulentum	
	At1g04820	5E-165	320C028569	30	Alpha tubulin	ABO61393	0	*Heterocapsa illdefina*	Dinophyta
	At1g04820	5E-58	320C028638	20	Beta tubulin	AAO49334	7E-139	*Amphidinium corpulentum*	Dinophyta
	At1g04820	4E-118	320C029246	59	Alpha-tubulin	ABV72532	6E-149	*Heterocapsa triquetra*	Dinophyta
	At1g04820	4E-68	320C029745	5	Alpha tubulin	ABO61393	2E-86	*Heterocapsa illdefina*	Dinophyta
	At1g04820	0	320C030351	51	Alpha tubulin	ABV72532	0	*Heterocapsa triquetra*	Dinophyta
Alpha-amylase	At1g69830	4E-54	320C008150	9	Probable alpha-amylase 2-like	XP_002980855	1E-65	*Selaginella moellendorffii*	Streptophyta
	At1g69830	4E-54	320C008150	9	Probable alpha-amylase 2-like	XP_002980855	1E-65	*Selaginella moellendorffii*	Streptophyta
	At1g69830	4E-63	320C010144	27	Glycoside hydrolase family 13 protein	XP_002369108	1E-87	*Toxoplasma gondii*	Apicomplexa
ATPase subunit	68259.m00094	1E-109	320C001833	23	ATP synthase alpha	ADV91187	0	*Karlodinium veneficum*	Dinophyta
	68259.m00094	4E-88	320C007795	15	F0f1 ATP synthase subunit alpha	ADV91186	3E-161	*Karlodinium veneficum*	Dinophyta
	68259.m00094	1E-71	320C012985	5	ATP synthase subunit alpha	ADV91186	8E-92	*Karlodinium veneficum*	Dinophyta
	68259.m00094	1E-71	320C012985	5	ATP synthase subunit alpha	ADV91186	8E-92	*Karlodinium veneficum*	Dinophyta
	68259.m00094	3E-140	320C013686	20	ATP synthase alpha subunit	ADV91186	0	*Karlodinium veneficum*	Dinophyta
	68259.m00094	4E-88	320C016592	11	ATP synthase alpha	ADV91186	2E-154	*Karlodinium veneficum*	Dinophyta
	68259.m00094	1E-176	320C019198	28	ATP synthase alpha	ADV91186	0	*Karlodinium veneficum*	Dinophyta
	68259.m00094	2E-70	320C022038	23	ATP synthase f1 subunit	ADV91187	3E-131	*Karlodinium veneficum*	Dinophyta
	68259.m00	2E-70	320C023621	15	ATP synthase	ADV91187	1E-141	*Karlodinium*	Dinophyta

	094				alpha			*veneficum*	
	68259.m00 094	2E-173	320C028631	35	ATP synthase alpha	ADV91186	0	*Karlodinium veneficum*	Dinophyta
ATPase alpha subunit	68258.m00 006	1E-101	320C001833	23	ATP synthase alpha	ADV91187	0	*Karlodinium veneficum*	Dinophyta
	68258.m00 006	7E-70	320C007795	15	F0f1 ATP synthase subunit alpha	ADV91186	3E-161	*Karlodinium veneficum*	Dinophyta
	68258.m00 006	1E-68	320C012985	5	ATP synthase subunit alpha	ADV91186	8E-92	*Karlodinium veneficum*	Dinophyta
	68258.m00 006	1E-68	320C012985	5	ATP synthase subunit alpha	ADV91186	8E-92	*Karlodinium veneficum*	Dinophyta
	68258.m00 006	4E-123	320C013686	20	ATP synthase alpha subunit	ADV91186	0	*Karlodinium veneficum*	Dinophyta
	68258.m00 006	8E-70	320C016592	11	ATP synthase alpha	ADV91186	2E-154	*Karlodinium veneficum*	Dinophyta
	68258.m00 006	8E-157	320C019198	28	ATP synthase alpha	ADV91186	0	*Karlodinium veneficum*	Dinophyta
	68258.m00 006	6E-71	320C022038	23	ATP synthase f1 subunit	ADV91187	3E-131	*Karlodinium veneficum*	Dinophyta
	68258.m00 006	7E-72	320C023621	15	ATP synthase alpha	ADV91187	1E-141	*Karlodinium veneficum*	Dinophyta
	68258.m00 006	9E-155	320C028631	35	ATP synthase alpha	ADV91186	0	*Karlodinium veneficum*	Dinophyta
	68258.m00 028	3E-51	320C003837	2	F0f1 ATP synthase subunit beta	ZP_01907032	6E-87	*Plesiocystis pacifica*	Proteobacteria
	68258.m00 028	3E-81	320C013463	14	H+ transporting ATP synthase beta subunit	ACU45001	5E-111	*Pfiesteria piscicida*	Dinophyta
	68258.m00 028	2E-77	320C013828	7	ATP H+ transporting f1 complex beta subunit	ABD77232	1E-114	*Tamandua tetradactyla*	Chordata
	68258.m00	7E-66	320C013922	6	ATP synthase	CCA23253	1E-90	*Albugo laibachii*	Oomycota

	028				beta subunit				
	68258.m00 028	3E-122	320C019667	7	ATP synthase beta	ACU45001	0	*Pfiesteria piscicida*	Dinophyta
	68258.m00 028	9E-54	320C028395	24	ATP synthase subunit partial	ACU45001	8E-75	*Pfiesteria piscicida*	Dinophyta
	68258.m00 028	3E-74	320C029923	27	ATP synthase beta	ACU45001	2E-115	*Pfiesteria piscicida*	Dinophyta
ATPase 70 kDa subunit	At1g78900	3E-130	320C002093	41	Vacuolar ATP synthase catalytic subunit	XP_002364944	1E-179	*Toxoplasma gondii*	Apicomplexa
	At1g78900	1E-80	320C003974	11	Vacuolar ATP synthase catalytic subunit a	XP_002780941	8E-108	*Perkinsus marinus*	Dinophyta
	At1g78900	3E-91	320C018058	21	Vacuolar ATP synthase catalytic subunit	XP_002765866	3E-142	*Perkinsus marinus*	Dinophyta
	At1g78900	2E-64	320C018157	25	Vacuolar ATP synthase catalytic subunit a	XP_002364944	5E-85	*Toxoplasma gondii*	Apicomplexa
	At1g78900	1E-81	320C019341	24	Vacuolar ATP synthase catalytic subunit	XP_002765866	2E-138	*Perkinsus marinus*	Dinophyta
	At1g78900	5E-51	320C019788	16	Vacuolar ATP synthase subunit	XP_002765866	7E-64	*Perkinsus marinus*	Dinophyta
	At1g78900	2E-65	320C020976	20	Vacuolar ATP synthase subunit	EEE25456	3E-85	*Toxoplasma gondii*	Apicomplexa
	At1g78900	6E-83	320C028819	12	Vacuolar ATP synthase subunit	XP_002780941	2E-110	*Perkinsus marinus*	Dinophyta
ATP-dependent Clp	At3g48870	4E-148	320C001286	248	Heat shock protein 101	XP_002328643	0	*Populus trichocarpa*	Streptophyta
protease ATP-	At3g48870	2E-150	320C005350	42	Clp protease	CCA24481	0	*Albugo laibachii*	Oomycota

binding subunit					ATP-binding subunit				
	At3g48870	1E-83	320C016224	8	Clp protease ATP binding subunit	XP_003063658	8E-91	*Micromonas pusilla*	Chlorophyta
	At3g48870	8E-129	320C018784	37	Heat shock protein 101	CCA14066	0	*Albugo laibachii*	Oomycota
	At3g48870	5E-63	320C024021	4	Heat shock protein 101	EGD82525	2E-104	*Salpingoeca* sp.	Choanozoa
	At3g48870	8E-59	320C024988	4	ATP-dependent clp protease ATP-binding subunit clpa homolog	YP_003002123	2E-61	*Aureococcus anophagefferens*	Heterokontophyta
	At3g48870	8E-59	320C024988	4	ATP-dependent clp protease ATP-binding subunit clpa homolog	YP_003002123	2E-61	*Aureococcus anophagefferens*	Heterokontophyta
	At5g50920	5E-162	320C001286	248	Heat shock protein 101	XP_002328643	0	*Populus trichocarpa*	Streptophyta
	At5g50920	1E-164	320C005350	42	Clp protease ATP-binding subunit	CCA24481	0	*Albugo laibachii*	Oomycota
	At5g50920	5E-85	320C016224	8	Clp protease ATP binding subunit	XP_003063658	8E-91	*Micromonas pusilla*	Chlorophyta
	At5g50920	3E-128	320C018784	37	Heat shock protein 101	CCA14066	0	*Albugo laibachii*	Oomycota
	At5g50920	2E-63	320C024021	4	Heat shock protein 101	EGD82525	2E-104	*Salpingoeca* sp.	Choanozoa
	At5g50920	2E-58	320C024988	4	ATP-dependent clp protease ATP-	YP_003002123	2E-61	*Aureococcus anophagefferens*	Heterokontophyta

					binding subunit clpa homolog				
	At5g50920	2E-58	320C024988	4	ATP-dependent clp protease ATP-binding subunit clpa homolog	YP_003002123	2E-61	*Aureococcus anophagefferens*	Heterokontophyta
Beta tubulin	At1g20010	3E-111	320C000206	20	Beta tubulin	AFX83590	1E-139	*Scrippsiella trochoidea*	Dinophyta
	At1g20010	2E-95	320C000325	40	Alpha tubulin	AFD34242	0	*Prorocentrum minimum*	Dinophyta
	At1g20010	8E-64	320C000621	89	Alpha tubulin	ABV72532	0	*Heterocapsa triquetra*	Dinophyta
	At1g20010	1E-105	320C001258	53	Alpha tubulin	ABV72532	0	*Heterocapsa triquetra*	Dinophyta
	At1g20010	3E-103	320C001846	54	Alpha tubulin	ABV22199	0	*Karlodinium veneficum*	Dinophyta
	At1g20010	2E-144	320C002623	56	Beta tubulin	AAO49334	0	*Amphidinium corpulentum*	Dinophyta
	At1g20010	6E-57	320C008076	9	Beta tubulin	XP_002788652	3E-99	*Perkinsus marinus*	Dinophyta
	At1g20010	0	320C009187	21	Beta tubulin	AFX83586	0	*Gymnodinium aureolum*	Dinophyta
	At1g20010	7E-132	320C010828	18	Beta tubulin	AFX83584	5E-173	*Alexandrium minutum*	Dinophyta
	At1g20010	9E-101	320C015009	7	Beta tubulin	ABR22560	1E-125	*Karenia brevis*	Dinophyta
	At1g20010	1E-105	320C018074	51	Alpha tubulin	ABV72532	0	*Heterocapsa triquetra*	Dinophyta
	At1g20010	4E-87	320C018199	70	Alpha tubulin	AFD34242	0	*Prorocentrum minimum*	Dinophyta
	At1g20010	0	320C019514	36	Beta tubulin	AFX83586	0	*Gymnodinium aureolum*	Dinophyta
	At1g20010	6E-71	320C019773	32	Beta tubulin	AAM02970	1E-92	*Crypthecodinium cohnii*	Dinophyta
	At1g20010	2E-53	320C019851	17	Alpha tubulin	ABJ80978	2E-168	*Peridinium limbatum*	Dinophyta
	At1g20010	1E-53	320C019853	22	Alpha tubulin	ABJ80978	9E-150	*Peridinium*	Dinophyta

								limbatum	
	At1g20010	1E-53	320C019854	48	Alpha tubulin	ABJ80978	9E-150	*Peridinium limbatum*	Dinophyta
	At1g20010	0	320C020372	19	Beta tubulin	AAM02970	0	*Crypthecodinium cohnii*	Dinophyta
	At1g20010	4E-118	320C020373	8	Beta tubulin	AAM02970	6E-166	*Crypthecodinium cohnii*	Dinophyta
	At1g20010	5E-92	320C021826	23	Beta tubulin	AFX83590	2E-110	*Scrippsiella trochoidea*	Dinophyta
	At1g20010	3E-124	320C021833	14	Beta- partial	AFX83582	7E-162	*Adenoides eludens*	Dinophyta
	At1g20010	1E-57	320C022013	20	Alpha tubulin	AAT09064	5E-129	*Bigelowiella natans*	Cercozoa
	At1g20010	2E-79	320C022460	11	Beta tubulin	P50261	7E-93	*oomycete-like MacKay2000*	-
	At1g20010	4E-83	320C022461	10	Beta tubulin	AEO99204	6E-91	*Perkinsus olseni*	Dinophyta
	At1g20010	4E-76	320C022580	11	Beta tubulin	ABR22560	2E-108	*Karenia brevis*	Dinophyta
	At1g20010	1E-105	320C023001	90	Alpha tubulin	ABV72532	0	*Heterocapsa triquetra*	Dinophyta
	At1g20010	2E-138	320C023598	30	Beta tubulin	AAM02970	0	*Crypthecodinium cohnii*	Dinophyta
	At1g20010	4E-111	320C026773	25	Beta tubulin	AAO49334	2E-139	*Amphidinium corpulentum*	Dinophyta
	At1g20010	9E-58	320C027029	30	Alpha tubulin	AAP46145	5E-63	*Pseudo-nitzschia multiseries*	Heterokontophyta
	At1g20010	1E-53	320C027960	18	Beta tubulin	ABR22560	2E-56	*Karenia brevis*	Dinophyta
	At1g20010	3E-120	320C028324	22	Beta-tubulin	AAP46146	1E-160	*Pseudo-nitzschia multiseries*	Heterokontophyta
	At1g20010	7E-76	320C028569	30	Alpha tubulin	ABO61393	0	*Heterocapsa illdefina*	Dinophyta
	At1g20010	5E-59	320C028595	16	Beta-tubulin	XP_001433506	2E-77	*Paramecium tetraurelia*	Ciliophora
	At1g20010	2E-110	320C028638	20	Beta-tubulin	AAO49334	7E-139	*Amphidinium corpulentum*	Dinophyta
	At1g20010	1E-53	320C028639	12	Beta tubulin	ABR22560	2E-56	*Karenia brevis*	Dinophyta
	At1g20010	1E-91	320C028761	13	Beta-tubulin	AFX83590	4E-108	*Scrippsiella*	Dinophyta

								trochoidea	
	At1g20010	8E-93	320C028988	18	Beta-tubulin	P50260	9E-114	*oomycete-like MacKay2000*	-
	At1g20010	3E-99	320C029738	59	Beta-tubulin	AAP46145	3E-127	*Pseudo-nitzschia multiseries*	Heterokontophyta
	At1g20010	1E-53	320C030086	39	Beta-tubulin	AAM02970	3E-67	*Crypthecodinium cohnii*	Dinophyta
	At1g20010	1E-91	320C030256	30	Beta-tubulin	AFX83590	4E-108	*Scrippsiella trochoidea*	Dinophyta
	At1g20010	4E-92	320C030351	51	Alpha tubulin	ABV72532	0	*Heterocapsa triquetra*	Dinophyta
Chloroplast ftsh protease	At1g50250	1E-62	320C002845	6	ATP-dependent metalloprotease	XP_674260	6E-145	*Plasmodium berghei*	Apicomplexa
	At1g50250	1E-56	320C005365	9	ATP-dependent metalloprotease	XP_002781856	4E-106	*Perkinsus marinus*	Dinophyta
	At1g50250	7E-52	320C005792	45	26s protease regulatory subunit	ABI14281	0	*Pfiesteria piscicida*	Dinophyta
	At1g50250	1E-56	320C008843	28	26s proteasome ATPase subunit	XP_627766	0	*Cryptosporidium parvum*	Apicomplexa
	At1g50250	1E-55	320C013906	8	26s protease regulatory subunit	XP_627766	0	*Cryptosporidium parvum*	Apicomplexa
	At1g50250	8E-65	320C017033	17	Cell division protein	XP_002768170	7E-83	*Perkinsus marinus*	Dinophyta
Clathrin heavy chain	At3g08530	0	320C006370	25	Clathrin heavy	XP_002767117	0	*Perkinsus marinus*	Dinophyta
	At3g08530	2E-107	320C007601	39	Clathrin heavy chain 1-like	AFN87702	1E-120	*Nicotiana tabacum*	Streptophyta
	At3g08530	2E-68	320C009228	37	Clathrin heavy chain 1-like	AFN87703	2E-69	*Nicotiana tabacum*	Streptophyta
	At3g08530	8E-69	320C012195	13	Clathrin heavy	XP_002767117	4E-88	*Perkinsus marinus*	Dinophyta

	At3g08530	1E-51	320C021194	4	Clathrin heavy	XP_002767117	1E-75	*Perkinsus marinus*	Dinophyta
Degp protease	At3g27925	5E-74	320C015487	18	2 peptidase	ABF73023	9E-133	*Karenia brevis*	Dinophyta
	At5g40200	3E-101	320C003524	12	Protease do-like 9-like	XP_002948745	6E-126	*Volvox carteri*	Chlorophyta
	At5g40200	2E-85	320C009274	10	Protease do-like 9-like	XP_002982828	7E-105	*Selaginella moellendorffii*	Streptophyta
Elongation factor 1-alpha	At1g07920	5E-72	320C003565	45	Translation elongation factor-like protein	ABV72555	0	*Heterocapsa rotundata*	Dinophyta
	At1g07920	1E-75	320C009855	22	Translation elongation factor-like protein	AAV34145	0	*Heterocapsa triquetra*	Dinophyta
	At1g07920	1E-55	320C011267	25	Translation elongation factor ef-subunit	XP_001745655	9E-128	*Monosiga brevicollis*	Choanozoa
	At1g07920	2E-56	320C017950	113	Translation elongation factor ef-subunit	ACO50119	7E-128	*Diplonema papillatum*	Euglenozoa
	At1g07920	1E-68	320C018781	41	Eukaryotic peptide chain release factor gtp-binding	XP_002775705	6E-164	*Perkinsus marinus*	Dinophyta
	At1g07920	0	320C019648	59	Translation elongation factor 1-alpha	XP_002904717	0	*Phytophthora infestans*	Oomycota
	At1g07920	2E-100	320C021503	6	Translation elongation factor 1-alpha	ABC85724	2E-149	*Schizochytrium aggregatum*	Labyrinthulomycota
	At1g07920	3E-92	320C022330	27	Translation elongation factor-like	AAV34145	0	*Heterocapsa triquetra*	Dinophyta

					protein				
Elongation factor Tu	At1g62750	1E-76	320C003745	11	Elongation factor	CCE34584	8E-116	*Claviceps purpurea*	Ascomycota
	At4g20360	2E-78	320C000209	11	Translation elongation factor tu	ZP_23326199	2E-127	*Desulfovibrio africanus*	Proteobacteria
	At4g20360	2E-58	320C001809	8	Translation elongation factor tu	ZP_01035088	2E-104	*Roseovarius* sp.	Proteobacteria
	At4g20360	5E-60	320C021917	11	Translation elongation factor tu	ZP_00998154	7E-62	*Oceanicola batsensis*	Proteobacteria
	At4g20360	5E-60	320C021917	11	Translation elongation factor tu	ZP_00998154	7E-62	*Oceanicola batsensis*	Proteobacteria
	At4g20360	1E-62	320C024244	31	Translation elongation factor tu	YP_001229832	3E-79	*Geobacter uraniireducens*	Proteobacteria
Expressed protein similar	At1g69840	3E-53	320C006366	11	Protein	EIE18919	6E-42	*Coccomyxa subellipsoidea*	Chlorophyta
to hypothetical protein GB	At1g69840	3E-53	320C006366	11	Protein	EIE18919	6E-42	*Coccomyxa subellipsoidea*	Chlorophyta
Ftsh protease	At2g30950	1E-66	320C002845	6	ATP-dependent metalloproteas e	XP_674260	6E-145	*Plasmodium berghei*	Apicomplexa
	At2g30950	2E-54	320C005792	45	26s protease regulatory subunit	ABI14281	0	*Pfiesteria piscicida*	Dinophyta
	At2g30950	8E-58	320C008843	28	26s proteasome ATPase subunit	XP_627766	0	*Cryptosporidium parvum*	Apicomplexa
	At2g30950	1E-58	320C013906	8	26s protease regulatory subunit	XP_627766	0	*Cryptosporidium parvum*	Apicomplexa
	At2g30950	4E-63	320C017033	17	Cell division protein	XP_002768170	7E-83	*Perkinsus marinus*	Dinophyta
	At5g42270	2E-61	320C002845	6	ATP-	XP_674260	6E-145	*Plasmodium*	Apicomplexa

					dependent metalloproteas e			*berghei*	
	At5g42270	4E-55	320C005365	9	ATP-dependent metalloproteas e	XP_002781856	4E-106	*Perkinsus marinus*	Dinophyta
	At5g42270	1E-52	320C005792	45	26s protease regulatory subunit	ABI14281	0	*Pfiesteria piscicida*	Dinophyta
	At5g42270	8E-58	320C008843	28	26s proteasome ATPase subunit	XP_627766	0	*Cryptosporidium parvum*	Apicomplexa
	At5g42270	1E-56	320C013906	8	26s protease regulatory subunit	XP_627766	0	*Cryptosporidium parvum*	Apicomplexa
	At5g42270	2E-65	320C017033	17	Cell division protein	XP_002768170	7E-83	*Perkinsus marinus*	Dinophyta
Intron-binding protein aquarius	At2g38770	5E-139	320C000524	27	Intron-binding protein aquarius	XP_002786398	1E-174	*Perkinsus marinus*	Dinophyta
	At2g38770	1E-59	320C002818	8	Intron-binding protein aquarius	EIE21771	5E-68	*Coccomyxa subellipsoidea*	Chlorophyta
Metalloproteas e	At3g19170	1E-57	320C005736	16	Peptidase m16c associated domain-containing protein	EFN52070	3E-72	*Chlorella variabilis*	Chlorophyta
	At3g19170	4E-58	320C017808	7	Presequence protease	XP_002977652	6E-63	*Selaginella moellendorffii*	Streptophyta
2-oxoglutarate / malate translocator	At5g19760	1E-56	320C002910	17	Oxoglutarate malate translocator	ADV91208	2E-75	*Karlodinium veneficum*	Dinophyta

					protein				
	At5g19760	3E-61	320C004116	20	2-oxoglutarate malate carrier	ADV91208	9E-84	*Karlodinium veneficum*	Dinophyta
	At5g19760	5E-69	320C010382	15	Dicarboxylate tricarboxylate carrier	CAC84545	1E-83	*Nicotiana tabacum*	Streptophyta
	At5g19760	8E-61	320C029111	27	Oxoglutarate malate translocator protein	ADV91208	4E-91	*Karlodinium veneficum*	Dinophyta
ADP,ATP carrier protein	At3g08580	1E-52	320C018733	28	ADP, ATP translocase	ABV25601	7E-147	*Pyrocystis lunula*	Dinophyta
	At3g08580	3E-64	320C027790	19	ADP, ATP translocase	XP_001747021	3E-73	*Monosiga brevicollis*	Choanozoa
	At3g08580	2E-53	320C029750	167	ADP, ATP translocase	ABV25601	5E-147	*Pyrocystis lunula*	Dinophyta
	At5g13490	1E-52	320C018733	28	ADP, ATP translocase	ABV25601	7E-147	*Pyrocystis lunula*	Dinophyta
	At5g13490	8E-51	320C020243	16	ADP, ATP translocase	NP_001083023	3E-94	*Danio rerio*	Chordata
	At5g13490	2E-65	320C027790	19	ADP, ATP translocase	XP_001747021	3E-73	*Monosiga brevicollis*	Choanozoa
	At5g13490	3E-53	320C029750	167	ADP, ATP translocase	ABV25601	5E-147	*Pyrocystis lunula*	Dinophyta
Processing peptidase	At3g02090	4E-57	320C029458	31	Organelle processing	ADV91174	2E-167	*Karlodinium veneficum*	Dinophyta
Peptidyl-prolyl isomerase protein	At5g13120	1E-52	320C000560	8	Cyclophilin	XP_001683327	9E-81	*Leishmania major*	Euglenozoa
	At5g13120	2E-52	320C000758	11	Peptidyl-prolyl cis-trans isomerase	XP_004294264	2E-64	*Fragaria vesca*	Streptophyta
	At5g13120	4E-52	320C001725	17	Peptidyl-prolyl cis-trans	EGZ28785	3E-117	*Phytophthora sojae*	Oomycota
	At5g13120	2E-54	320C011997	8	Cyclophilin	BAM13284	1E-73	*Oryza officinalis*	Streptophyta
	At5g13120	2E-60	320C016005	7	Cyclophilin	ABI14285	4E-86	*Pfiesteria piscicida*	Dinophyta
	At5g13120	4E-58	320C017360	15	2-peptidylprolyl	ADC80505	4E-68	*Conus novaehollandiae*	Mollusca

					isomerase a				
	At5g13120	4E-65	320C021326	22	Peptidyl-prolyl cis-trans isomerase b precursor	ABI14285	3E-121	*Pfiesteria piscicida*	Dinophyta
	At5g13120	1E-54	320C026148	43	Peptidyl-prolyl cis-trans isomerase	BAE92296	3E-81	*Cryptomeria japonica*	Streptophyta
	At5g13120	9E-54	320C026374	11	Cyclophilin-like protein	ABI14282	1E-75	*Pfiesteria piscicida*	Dinophyta
Peptidylprolyl isomerase ROC4	At3g62030	6E-52	320C000560	8	Cyclophilin 11	XP_001683327	9E-81	*Leishmania major*	Euglenozoa
	At3g62030	3E-51	320C000758	11	Peptidyl-prolyl cis-trans isomerase	XP_004294264	2E-64	*Fragaria vesca*	Streptophyta
	At3g62030	1E-55	320C001725	17	Peptidyl-prolyl cis-trans	EGZ28785	3E-117	*Phytophthora sojae*	Oomycota
	At3g62030	1E-60	320C016005	7	Cyclophilin b	ABI14285	4E-86	*Pfiesteria piscicida*	Dinophyta
	At3g62030	2E-60	320C017360	15	2-peptidylprolyl isomerase a	ADC80505	4E-68	*Conus novaehollandiae*	Mollusca
	At3g62030	4E-53	320C018648	11	Peptidyl-prolyl cis-trans isomerase h	ABV22252	5E-123	*Karlodinium veneficum*	Dinophyta
	At3g62030	1E-63	320C021326	22	Peptidyl-prolyl cis-trans isomerase b precursor	ABI14285	3E-121	*Pfiesteria piscicida*	Dinophyta
	At3g62030	3E-55	320C026148	43	Peptidyl-prolyl cis-	BAE92296	3E-81	*Cryptomeria japonica*	Streptophyta

					trans isomerase				
	At3g62030	6E-52	320C026374	11	Cyclophilin-like protein	ABI14282	1E-75	*Pfiesteria piscicida*	Dinophyta
	At3g62030	6E-52	320C026374	11	Cyclophilin-like protein	ABI14282	1E-75	*Pfiesteria piscicida*	Dinophyta
Prohibitin	At3g27280	1E-66	320C006439	16	Prohibitin	CCF73247	1E-104	*Babesia microti*	Apicomplexa
	At1g03860	5E-74	320C006439	16	Prohibitin	CCF73247	1E-104	*Babesia microti*	Apicomplexa
Protease hhoa	At4g18370	1E-51	320C015487	18	2 peptidase	ABF73023	9E-133	*Karenia brevis*	Dinophyta
Pullulanase	At5g04360	8E-58	320C006748	8	Pullulanase	CBI31395	3E-59	*Vitis vinifera*	Streptophyta
	At5g04360	8E-58	320C006748	8	Pullulanase	CBI31395	3E-59	*Vitis vinifera*	Streptophyta
	At5g04360	8E-58	320C006748	8	Pullulanase	CBI31395	3E-59	*Vitis vinifera*	Streptophyta
Ruv DNA-helicase-related protein	At5g22330	0	320C006487	21	Dna tbp-interacting protein	XP_002772912	0	*Perkinsus marinus*	Dinophyta
	At5g22330	1E-102	320C010956	45	Ruvb-like 2-like	XP_002774119	0	*Perkinsus marinus*	Dinophyta
Ruvb DNA helicase	At3g49830	6E-102	320C006487	21	Dna tbp-interacting protein	XP_002772912	0	*Perkinsus marinus*	Dinophyta
	At3g49830	0	320C010956	45	Ruvb-like 2-like	XP_002774119	0	*Perkinsus marinus*	Dinophyta
	At5g67630	1E-103	320C006487	21	Dna tbp-interacting protein	XP_002772912	0	*Perkinsus marinus*	Dinophyta
	At5g67630	0	320C010956	45	Ruvb-like 2-like	XP_002774119	0	*Perkinsus marinus*	Dinophyta
SEX1 protein	At1g10760	7E-75	320C002166	48	Alpha-glucan water chloroplastic-like	XP_003884753	8E-87	*Neospora caninum*	Apicomplexa
	At1g10760	7E-75	320C002166	48	Alpha-glucan water chloroplastic-like	XP_003884753	8E-87	*Neospora caninum*	Apicomplexa
Structural maintenance of chromosomes	At3g54670	3E-51	320C009596	28	Condensin complex component	XP_626475	7E-40	*Cryptosporidium parvum*	Apicomplexa

	(SMC) - like protein 14S cohesin SMC1 subunit									
	Thylakoid bound ascorbate peroxidase	At1g77490	6E-62	320C018229	25	Ascorbate peroxidase	XP_001742299	6E-74	*Monosiga brevicollis*	Choanozoa
Genetic information processing	20S proteasome alpha subunit D	At3g51260	8E-75	320C000392	22	Proteasome subunit alpha type	XP_002764909	5E-107	*Perkinsus marinus*	Dinophyta
		At5g66140	8E-75	320C000392	22	Proteasome subunit alpha type	XP_002764909	5E-107	*Perkinsus marinus*	Dinophyta
	20S proteasome beta subunit F1	At3g60820	1E-55	320C011831	14	Proteasome subunit beta	XP_002782634	8E-76	*Perkinsus marinus*	Dinophyta
		At3g60820	6E-51	320C028759	17	Proteasome subunit beta	XP_002782634	2E-68	*Perkinsus marinus*	Dinophyta
	40S ribosomal protein S13	At3g60770	1E-53	320C024331	6	40s ribosomal protein	ABV72537	3E-77	*Heterocapsa triquetra*	Dinophyta
		At3g60770	3E-52	320C029266	15	40s ribosomal protein	ABV72537	9E-70	*Heterocapsa triquetra*	Dinophyta
	40S ribosomal protein S14	At2g36160	8E-62	320C003449	11	40s ribosomal protein s14	EMF11197	2E-65	*Mycosphaerella populorum*	Ascomycota
		At2g36160	2E-69	320C008629	15	40s ribosomal protein s14	EMF11197	3E-68	*Mycosphaerella populorum*	Ascomycota
		At2g36160	5E-69	320C017564	10	40s ribosomal protein s14	EMF11197	3E-68	*Mycosphaerella populorum*	Ascomycota
		At2g36160	6E-65	320C023294	8	40s ribosomal protein s14	EGB02316	8E-78	*Aureococcus anophagefferens*	Heterokontophyta
		At2g36160	6E-62	320C028626	13	40s ribosomal protein s14	XP_004365495	3E-63	*Capsaspora owczarzaki*	Choanozoa
	40S ribosomal protein S16	At2g09990	8E-56	320C002256	25	40s ribosomal protein s16-like	XP_002527855	5E-70	*Ricinus communis*	Streptophyta
		At2g09990	2E-55	320C006964	8	40s ribosomal	ADR71283	4E-69	*Hevea brasiliensis*	Streptophyta

					protein s16-like				
	At2g09990	5E-56	320C008864	19	40s ribosomal protein s16-like	XP_003518703	2E-70	*Glycine max*	Streptophyta
	At2g09990	1E-53	320C015209	6	40s ribosomal protein s16	EGZ14092	3E-70	*Phytophthora sojae*	Oomycota
	At3g04230	1E-52	320C002256	25	40s ribosomal protein s16-like	XP_002527855	5E-70	*Ricinus communis*	Streptophyta
	At3g04230	8E-53	320C006964	8	40s ribosomal protein s16-like	ADR71283	4E-69	*Hevea brasiliensis*	Streptophyta
	At3g04230	3E-53	320C008864	19	40s ribosomal protein s16-like	XP_003518703	2E-70	*Glycine max*	Streptophyta
	At3g04230	7E-53	320C015209	6	40s ribosomal protein	EGZ14092	3E-70	*Phytophthora sojae*	Oomycota
	At3g04770	7E-59	320C000468	22	40s ribosomal protein	XP_002769858	4E-85	*Perkinsus marinus*	Dinophyta
	At3g04770	2E-63	320C000925	11	40s ribosomal protein	XP_002368705	4E-99	*Toxoplasma gondii*	Apicomplexa
	At3g04770	2E-51	320C005729	23	40s ribosomal protein	XP_002368705	1E-78	*Toxoplasma gondii*	Apicomplexa
	At3g04770	6E-61	320C018800	30	40s ribosomal protein	XP_726642	2E-88	*Plasmodium yoelii*	Apicomplexa
	At3g04770	5E-66	320C019631	17	40s ribosomal protein	XP_002782805	9E-98	*Perkinsus marinus*	Dinophyta
	At3g04770	4E-60	320C020195	11	40s ribosomal protein	XP_002368705	3E-93	*Toxoplasma gondii*	Apicomplexa
40S ribosomal protein S2	At1g58380	1E-91	320C008068	11	Ribosomal protein s2	AER57857	4E-89	*Acytostelium subglobosum*	Amoebozoa
	At1g58380	1E-91	320C008068	11	Ribosomal protein s2	AER57857	4E-89	*Acytostelium subglobosum*	Amoebozoa
	At1g58380	1E-91	320C008068	11	Ribosomal protein s2	AER57857	4E-89	*Acytostelium subglobosum*	Amoebozoa
	At1g58380	6E-85	320C017714	26	40s ribosomal protein s2	CCA16359	7E-109	*Albugo laibachii*	Oomycota
	At1g58380	1E-56	320C020381	135	40s ribosomal	XP_002785321	2E-61	*Perkinsus*	Dinophyta

					protein			*marinus*	
40S ribosomal protein S3	At2g31610	2E-87	320C003466	161	40s ribosomal protein s3	XP_002780125	1E-101	*Perkinsus marinus*	Dinophyta
	At2g31610	8E-53	320C005222	34	40s ribosomal protein s3	XP_002780125	3E-66	*Perkinsus marinus*	Dinophyta
	At2g31610	3E-88	320C009769	9	40s ribosomal protein	AET50521	1E-101	*Eimeria tenella*	Apicomplexa
	At2g31610	2E-72	320C017588	14	40s ribosomal protein s3	XP_002767474	2E-82	*Perkinsus marinus*	Dinophyta
	At2g31610	2E-88	320C017662	56	40s ribosomal protein	AET50521	4E-103	*Eimeria tenella*	Apicomplexa
	At2g31610	4E-57	320C018847	14	40s ribosomal protein	EGB06804	1E-74	*Aureococcus anophagefferens*	Heterokontophyta
	At2g31610	2E-88	320C019938	29	40s ribosomal protein	XP_002780125	1E-103	*Perkinsus marinus*	Dinophyta
	At2g31610	8E-85	320C021926	16	40s ribosomal protein s3-3-like	EGB06804	3E-109	*Aureococcus anophagefferens*	Heterokontophyta
	At2g31610	1E-89	320C023799	28	40s ribosomal protein s3	AET50521	2E-104	*Eimeria tenella*	Apicomplexa
	At2g31610	2E-76	320C027717	46	40s ribosomal protein	AET50521	4E-86	*Eimeria tenella*	Apicomplexa
	At2g31610	8E-79	320C029755	12	40s ribosomal protein s3	XP_002780125	4E-89	*Perkinsus marinus*	Dinophyta
	At2g31610	3E-65	320C030241	22	40s ribosomal protein	XP_002767926	9E-71	*Perkinsus marinus*	Dinophyta
40S ribosomal protein S3A	At3g04840	3E-85	320C001772	43	40s ribosomal protein	ADV03017	9E-113	*Karlodinium veneficum*	Dinophyta
	At3g04840	4E-53	320C010257	14	S-phase-specific ribosomal protein	EGO01811	1E-66	*Serpula lacrymans*	Basidiomycota
	At3g04840	1E-64	320C015943	17	40s ribosomal protein	ADV03017	1E-98	*Karlodinium veneficum*	Dinophyta
	At3g04840	7E-85	320C022305	25	40s ribosomal protein s3a-	XP_002901276	4E-105	*Phytophthora infestans*	Oomycota

					like				
	At3g04840	4E-55	320C028827	10	40s ribosomal protein	ADV03017	2E-84	*Karlodinium veneficum*	Dinophyta
	At3g05590	8E-65	320C000048	28	60s ribosomal protein l18	ADV03016	4E-105	*Karlodinium veneficum*	Dinophyta
	At3g05590	7E-54	320C003601	10	60s ribosomal protein l18	ADV03016	3E-82	*Karlodinium veneficum*	Dinophyta
	At3g05590	2E-55	320C007234	16	40s ribosomal protein	XP_001419602	5E-66	*Ostreococcus lucimarinus*	Chlorophyta
	At3g05590	6E-65	320C010122	21	60s ribosomal protein l18	ADV03016	2E-105	*Karlodinium veneficum*	Dinophyta
	At3g05590	2E-64	320C011075	12	60s ribosomal protein l18	ADV03016	3E-104	*Karlodinium veneficum*	Dinophyta
	At3g05590	1E-64	320C014355	37	60s ribosomal protein l18	ADV03016	7E-104	*Karlodinium veneficum*	Dinophyta
	At3g05590	1E-52	320C016252	5	40s ribosomal protein	XP_001419602	2E-71	*Ostreococcus lucimarinus*	Chlorophyta
	At3g05590	3E-65	320C024806	21	60s ribosomal protein l18	ADV03016	2E-105	*Karlodinium veneficum*	Dinophyta
	At3g07110	7E-51	320C001424	11	60s ribosomal protein	ABZ04228	5E-69	*Lineus viridis*	Nemertea
	At3g07110	2E-59	320C008783	10	60s ribosomal protein l16	EGD77624	2E-73	*Salpingoeca* sp.	Choanozoa
	At3g07110	1E-55	320C008960	13	60s ribosomal protein l16	P93099	7E-73	*Cyanophora paradoxa*	Glaucophyta
	At3g07110	2E-51	320C018413	38	60s ribosomal protein l13a	ABZ04228	8E-71	*Lineus viridis*	Nemertea
	At3g07110	8E-57	320C020003	17	60s ribosomal protein	ABZ04228	8E-80	*Lineus viridis*	Nemertea
40S ribosomal protein S4	At2g17360	1E-68	320C001516	26	40s ribosomal protein s4	XP_002767227	3E-90	*Perkinsus marinus*	Dinophyta
	At2g17360	1E-102	320C002191	42	40s ribosomal protein s4	XP_002767227	5E-142	*Perkinsus marinus*	Dinophyta
	At2g17360	2E-81	320C002968	19	40s ribosomal protein	XP_002767227	3E-113	*Perkinsus marinus*	Dinophyta
	At2g17360	5E-59	320C003898	6	Ribosomal protein s4	XP_002767227	3E-75	*Perkinsus marinus*	Dinophyta
	At2g17360	2E-71	320C018340	43	40s ribosomal protein x	XP_002772984	1E-81	*Perkinsus marinus*	Dinophyta

	At2g17360	2E-67	320C019522	7	40s ribosomal protein s4-like	XP_002268192	2E-81	*Vitis vinifera*	Streptophyta
	At2g17360	7E-93	320C022413	10	40s ribosomal protein s4-like	EMJ13066	2E-102	*Prunus persica*	Streptophyta
	At2g17360	4E-100	320C023105	23	40s ribosomal protein x isoform	XP_002767227	4E-140	*Perkinsus marinus*	Dinophyta
	At2g17360	1E-89	320C029372	24	40s ribosomal protein s4	XP_002767227	2E-125	*Perkinsus marinus*	Dinophyta
40S ribosomal protein S6	At4g31700	2E-71	320C000192	48	40s ribosomal protein	ABI14420	1E-116	*Karlodinium veneficum*	Dinophyta
	At4g31700	9E-61	320C001331	25	40s ribosomal protein	ABI14420	3E-111	*Karlodinium veneficum*	Dinophyta
	At4g31700	9E-73	320C001737	32	40s ribosomal protein	ABI14420	2E-121	*Karlodinium veneficum*	Dinophyta
	At4g31700	5E-69	320C002873	33	40s ribosomal protein	ABI14420	8E-124	*Karlodinium veneficum*	Dinophyta
	At4g31700	2E-70	320C003077	14	40s ribosomal protein s6	EGZ15844	2E-89	*Phytophthora sojae*	Oomycota
	At4g31700	1E-73	320C003276	27	40s ribosomal protein	ABI14420	5E-125	*Karlodinium veneficum*	Dinophyta
	At4g31700	6E-70	320C003353	29	40s ribosomal protein	ABI14420	4E-118	*Karlodinium veneficum*	Dinophyta
	At4g31700	9E-70	320C018308	71	40s ribosomal protein	ABI14420	2E-123	*Karlodinium veneficum*	Dinophyta
	At4g31700	2E-59	320C022528	14	40s ribosomal protein	ACU45262	8E-105	*Karlodinium veneficum*	Dinophyta
	At4g31700	4E-67	320C024523	16	40s ribosomal protein	ABI14420	9E-109	*Karlodinium veneficum*	Dinophyta
	At4g31700	2E-58	320C025513	24	40s ribosomal protein s6	EME29222	2E-77	*Galdieria sulphuraria*	Rhodophyta
	At4g31700	5E-68	320C028850	31	40s ribosomal protein	ABI14420	2E-108	*Karlodinium veneficum*	Dinophyta
40S ribosomal protein S8	At5g20290	2E-51	320C003472	3	Ribosomal protein s8	XP_002293544	7E-68	*Thalassiosira pseudonana*	Heterokontophyta
	At5g20290	5E-65	320C003617	12	40s ribosomal	AGH70192	8E-73	*Placozoa* sp.	Placozoa

					protein s8				
	At5g20290	8E-55	320C021666	16	40s ribosomal protein	XP_002329256	1E-53	*Populus trichocarpa*	Streptophyta
	At5g20290	4E-55	320C025357	7	40s ribosomal protein s8	XP_001893693	4E-68	*Brugia malayi*	Nematoda
	At5g20290	3E-67	320C026245	38	40s ribosomal protein s8	XP_002771622	1E-77	*Perkinsus marinus*	Dinophyta
	At5g20290	5E-68	320C029071	19	40s ribosomal protein	XP_002787865	7E-77	*Perkinsus marinus*	Dinophyta
40S ribosomal protein S9	At5g15200	2E-76	320C002450	12	40s ribosomal protein	XP_001351758	4E-82	*Plasmodium falciparum*	Apicomplexa
	At5g15200	2E-58	320C003993	7	40s ribosomal protein	XP_002259613	1E-71	*Plasmodium knowlesi*	Apicomplexa
	At5g15200	2E-60	320C005303	16	Ribosomal protein component of cytosolic 80s ribosome and 40s small subunit	EKU23223	3E-59	*Nannochloropsis gaditana*	Heterokontophyta
	At5g15200	2E-76	320C006114	16	40s ribosomal protein	XP_002259613	8E-82	*Plasmodium knowlesi*	Apicomplexa
	At5g15200	2E-76	320C016621	14	40s ribosomal protein	XP_002259613	7E-82	*Plasmodium knowlesi*	Apicomplexa
	At5g15200	9E-60	320C017802	28	40s ribosomal protein	CCF75909	3E-60	*Babesia microti*	Apicomplexa
	At5g15200	4E-77	320C020342	36	40s ribosomal protein	XP_002259613	6E-82	*Plasmodium knowlesi*	Apicomplexa
	At5g15200	1E-59	320C024834	19	40s ribosomal protein	CCF75909	9E-61	*Babesia microti*	Apicomplexa
40S ribosomal protein SA	At1g72370	4E-60	320C000468	22	40s ribosomal protein	XP_002769858	4E-85	*Perkinsus marinus*	Dinophyta
	At1g72370	6E-75	320C000925	11	40s ribosomal protein	XP_002368705	4E-99	*Toxoplasma gondii*	Apicomplexa
	At1g72370	2E-62	320C005729	23	40s ribosomal protein	XP_002368705	1E-78	*Toxoplasma gondii*	Apicomplexa
	At1g72370	1E-73	320C018800	30	40s ribosomal protein	XP_726642	2E-88	*Plasmodium yoelii*	Apicomplexa
	At1g72370	2E-79	320C019631	17	40s ribosomal	XP_002782805	9E-98	*Perkinsus*	Dinophyta

					protein			*marinus*	
	At1g72370	5E-67	320C020195	11	40s ribosomal protein	XP_002368705	3E-93	*Toxoplasma gondii*	Apicomplexa
60S acidic ribosomal protein P0	At3g09200	5E-57	320C003161	39	60s acidic ribosomal protein p0	XP_002784721	1E-76	*Perkinsus marinus*	Dinophyta
	At3g09200	9E-59	320C005620	43	60s acidic ribosomal protein p0	XP_002765467	3E-90	*Perkinsus marinus*	Dinophyta
	At3g09200	9E-59	320C005620	43	60s acidic ribosomal protein p0	XP_002765467	3E-90	*Perkinsus marinus*	Dinophyta
	At3g09200	6E-76	320C009018	13	60s acidic ribosomal protein p0	EGZ26646	1E-94	*Phytophthora sojae*	Oomycota
	At3g09200	3E-63	320C018700	20	60s acidic ribosomal protein p0	XP_002765467	5E-95	*Perkinsus marinus*	Dinophyta
	At3g09200	3E-64	320C020573	28	60s acidic ribosomal protein p0	XP_002784721	7E-92	*Perkinsus marinus*	Dinophyta
	At3g09820	6E-108	320C018620	84	Adenosine kinase	EIE21409	2E-137	*Coccomyxa subellipsoidea*	Chlorophyta
	At3g11940	3E-81	320C002670	13	40s ribosomal protein	ABI14354	2E-135	*Pfiesteria piscicida*	Dinophyta
	At3g11940	6E-67	320C003344	9	40s ribosomal protein	EGB11317	1E-92	*Aureococcus anophagefferens*	Heterokontophyta
	At3g11940	2E-80	320C004446	20	40s ribosomal protein	ABI14354	1E-136	*Pfiesteria piscicida*	Dinophyta
	At3g11940	2E-80	320C006432	8	40s ribosomal protein	ABI14354	3E-135	*Pfiesteria piscicida*	Dinophyta
	At3g11940	3E-56	320C006475	6	40s ribosomal protein s5	XP_003062276	5E-59	*Micromonas pusilla*	Chlorophyta
	At3g11940	3E-56	320C006475	6	40s ribosomal protein s5	XP_003062276	5E-59	*Micromonas pusilla*	Chlorophyta
	At3g11940	2E-80	320C008992	20	40s ribosomal	ABI14354	2E-139	*Pfiesteria*	Dinophyta

					protein			*piscicida*	
	At3g11940	3E-72	320C014417	12	40s ribosomal protein s5	ABK23207	6E-96	*Picea sitchensis*	Streptophyta
	At3g11940	5E-70	320C014432	8	40s ribosomal protein	ABI14354	5E-114	*Pfiesteria piscicida*	Dinophyta
	At3g11940	5E-53	320C017154	22	40s ribosomal protein s5	ABI14354	5E-94	*Pfiesteria piscicida*	Dinophyta
	At3g11940	7E-81	320C017750	26	40s ribosomal protein	ABI14354	3E-128	*Pfiesteria piscicida*	Dinophyta
	At3g11940	2E-70	320C023936	40	40s ribosomal protein	ABI14354	5E-114	*Pfiesteria piscicida*	Dinophyta
	At3g11940	1E-66	320C028406	12	40s ribosomal protein	ABI14354	5E-115	*Pfiesteria piscicida*	Dinophyta
	At3g11940	5E-70	320C028709	16	40s ribosomal protein	ABI14354	2E-115	*Pfiesteria piscicida*	Dinophyta
60S ribosomal protein L10	At1g14320	2E-75	320C005115	13	60s ribosomal protein l10-like	CBN75612	1E-106	*Ectocarpus siliculosus*	Heterokontophyta
	At1g14320	5E-53	320C008061	19	Ribosomal protein l10	ACI68991	7E-65	*Salmo salar*	Chordata
	At1g14320	4E-80	320C015973	8	60s ribosomal protein l10-like	NP_001054759	7E-79	*Oryza sativa*	Streptophyta
	At1g14320	4E-80	320C015973	8	60s ribosomal protein l10-like	NP_001054759	7E-79	*Oryza sativa*	Streptophyta
	At1g14320	2E-59	320C022211	28	60s ribosomal protein	XP_002777357	2E-84	*Perkinsus marinus*	Dinophyta
	At1g14320	1E-70	320C023424	29	60s ribosomal protein	XP_002777357	2E-99	*Perkinsus marinus*	Dinophyta
	At1g14320	2E-73	320C027798	51	60s ribosomal protein	XP_002777357	6E-104	*Perkinsus marinus*	Dinophyta
60S ribosomal protein L11	At2g42740	2E-67	320C001273	29	60s ribosomal protein l11	ADV03013	7E-110	*Karlodinium veneficum*	Dinophyta
	At2g42740	1E-67	320C002305	17	Ribosomal protein l11	ADV03013	4E-108	*Karlodinium veneficum*	Dinophyta
	At2g42740	1E-67	320C002584	16	60s ribosomal protein l11	ADV03013	3E-110	*Karlodinium veneficum*	Dinophyta
	At2g42740	1E-67	320C003697	17	60s ribosomal	ADV03013	1E-109	*Karlodinium*	Dinophyta

					protein l11-like			*veneficum*	
	At2g42740	1E-67	320C023170	24	60s ribosomal protein l11	ADV03013	3E-110	*Karlodinium veneficum*	Dinophyta
	At2g42740	9E-59	320C023171	14	Ribosomal protein l11	ADV03047	6E-92	*Amphidinium carterae*	Dinophyta
	At2g42740	1E-67	320C023878	11	Ribosomal protein l11	ADV03013	1E-105	*Karlodinium veneficum*	Dinophyta
60S ribosomal protein L17	At1g27400	6E-51	320C001589	17	60s ribosomal protein	ADV03056	8E-71	*Amphidinium carterae*	Dinophyta
	At1g27400	4E-51	320C027101	24	Ribosomal protein l17	ADV03056	5E-71	*Amphidinium carterae*	Dinophyta
60S ribosomal protein L4/L1	At3g09630	5E-61	320C005728	13	60s ribosomal subunit protein	BAA78600	3E-75	*Chlamydomonas* sp.	Chlorophyta
	At3g09630	9E-111	320C007801	75	60s ribosomal subunit protein	XP_002769741	1E-131	*Perkinsus marinus*	Dinophyta
	At3g09630	7E-75	320C014113	7	60s ribosomal	XP_001694804	3E-77	*Chlamydomonas reinhardtii*	Chlorophyta
	At3g09630	1E-108	320C017879	58	60s ribosomal subunit protein	XP_002769741	5E-129	*Perkinsus marinus*	Dinophyta
	At3g09630	5E-106	320C029566	231	60s ribosomal subunit protein	XP_002769741	9E-120	*Perkinsus marinus*	Dinophyta
	At5g02870	9E-60	320C005728	13	60s ribosomal subunit protein	BAA78600	3E-75	*Chlamydomonas* sp.	Chlorophyta
	At5g02870	3E-110	320C007801	75	60s ribosomal subunit protein	XP_002769741	1E-131	*Perkinsus marinus*	Dinophyta
	At5g02870	1E-75	320C014113	7	60s ribosomal subunit protein	XP_001694804	3E-77	*Chlamydomonas reinhardtii*	Chlorophyta
	At5g02870	4E-108	320C017879	58	60s ribosomal	XP_002769741	5E-129	*Perkinsus*	Dinophyta

					subunit protein			*marinus*	
	At5g02870	3E-105	320C029566	231	60s ribosomal subunit protein	XP_002769741	9E-120	*Perkinsus marinus*	Dinophyta
60S ribosomal protein L5	At3g25520	7E-60	320C000042	49	60s ribosomal protein	XP_666127	3E-84	*Cryptosporidium hominis*	Apicomplexa
	At3g25520	4E-62	320C000088	40	60s ribosomal protein	XP_002784462	1E-87	*Perkinsus marinus*	Dinophyta
	At3g25520	2E-53	320C000234	19	60s ribosomal protein	XP_666127	9E-75	*Cryptosporidium hominis*	Apicomplexa
	At3g25520	1E-58	320C000404	26	60s ribosomal protein l5	XP_002176750	8E-81	*Phaeodactylum tricornutum*	Heterokontophyta
	At3g25520	9E-80	320C003552	67	Ribosomal protein l5	XP_625505	1E-113	*Cryptosporidium parvum*	Apicomplexa
	At3g25520	3E-58	320C007736	7	60s ribosomal protein l5	XP_004337717	5E-67	*Acanthamoeba castellanii*	Amoebozoa
	At3g25520	3E-58	320C007736	7	60s ribosomal protein l5	XP_004337717	5E-67	*Acanthamoeba castellanii*	Amoebozoa
	At3g25520	3E-58	320C007736	7	60s ribosomal protein l5	XP_004337717	5E-67	*Acanthamoeba castellanii*	Amoebozoa
	At3g25520	1E-66	320C019045	33	60s ribosomal protein	XP_002784462	4E-92	*Perkinsus marinus*	Dinophyta
	At3g25520	1E-53	320C019923	38	60s ribosomal protein	XP_002784462	2E-75	*Perkinsus marinus*	Dinophyta
	At3g25520	4E-60	320C024910	36	60s ribosomal protein	XP_666127	8E-86	*Cryptosporidium hominis*	Apicomplexa
	At3g25520	4E-59	320C025199	53	60s ribosomal protein	XP_666127	1E-82	*Cryptosporidium hominis*	Apicomplexa
60S ribosomal protein L7	At2g01250	3E-77	320C000799	15	Ribosomal protein l7	XP_001750829	4E-93	*Monosiga brevicollis*	Choanozoa
	At2g01250	3E-69	320C016960	16	60s ribosomal protein l7	CBN78186	2E-82	*Ectocarpus siliculosus*	Heterokontophyta
	At2g01250	2E-61	320C017972	23	60s ribosomal protein	XP_002775844	8E-67	*Perkinsus marinus*	Dinophyta
	At2g01250	6E-56	320C018280	151	60s ribosomal protein	XP_002775844	2E-60	*Perkinsus marinus*	Dinophyta
	At2g01250	3E-55	320C018332	13	60s ribosomal protein l7	XP_002775844	2E-64	*Perkinsus marinus*	Dinophyta

At2g01250	9E-73	320C026414	47	60s ribosomal protein	XP_002775844	1E-78	*Perkinsus marinus*	Dinophyta
At2g01250	9E-73	320C026414	47	60s ribosomal protein	XP_002775844	1E-78	*Perkinsus marinus*	Dinophyta
At2g47610	4E-58	320C008137	21	60s ribosomal protein l7a	XP_625504	3E-76	*Cryptosporidium parvum*	Apicomplexa
At2g47610	8E-51	320C014999	11	60s ribosomal protein l7a	CCQ18597	1E-54	*Sycon ciliatum*	Porifera
At2g47610	3E-82	320C015863	30	60s ribosomal protein l7a	XP_003882835	5E-105	*Neospora caninum*	Apicomplexa
At2g47610	2E-81	320C018107	25	60s ribosomal protein l7a	XP_003882835	1E-100	*Neospora caninum*	Apicomplexa
At2g47610	2E-70	320C018722	41	60s ribosomal protein	XP_003882835	3E-82	*Neospora caninum*	Apicomplexa
At2g47610	1E-77	320C020640	35	60s ribosomal protein	XP_666128	8E-95	*Cryptosporidium hominis*	Apicomplexa
At2g47610	4E-73	320C021070	28	60s ribosomal protein	XP_003882835	5E-88	*Neospora caninum*	Apicomplexa
At3g13580	7E-78	320C000799	15	Ribosomal protein l7	XP_001750829	4E-93	*Monosiga brevicollis*	Choanozoa
At3g13580	6E-71	320C016960	16	60s ribosomal protein l7	CBN78186	2E-82	*Ectocarpus siliculosus*	Heterokontophyta
At3g13580	2E-59	320C017972	23	60s ribosomal protein	XP_002775844	8E-67	*Perkinsus marinus*	Dinophyta
At3g13580	1E-54	320C018280	151	60s ribosomal protein	XP_002775844	2E-60	*Perkinsus marinus*	Dinophyta
At3g13580	2E-53	320C018332	13	60s ribosomal protein l7	XP_002775844	2E-64	*Perkinsus marinus*	Dinophyta
At3g13580	2E-71	320C026414	47	60s ribosomal protein	XP_002775844	1E-78	*Perkinsus marinus*	Dinophyta
At3g13580	2E-71	320C026414	47	60s ribosomal protein	XP_002775844	1E-78	*Perkinsus marinus*	Dinophyta
At2g44120	6E-77	320C000799	15	Ribosomal protein l7	XP_001750829	4E-93	*Monosiga brevicollis*	Choanozoa
At2g44120	2E-69	320C016960	16	60s ribosomal protein l7	CBN78186	2E-82	*Ectocarpus siliculosus*	Heterokontophyta

	At2g44120	1E-59	320C017972	23	60s ribosomal protein	XP_002775844	8E-67	*Perkinsus marinus*	Dinophyta
	At2g44120	4E-56	320C018280	151	60s ribosomal protein	XP_002775844	2E-60	*Perkinsus marinus*	Dinophyta
	At2g44120	2E-55	320C018332	13	60s ribosomal protein l7	XP_002775844	2E-64	*Perkinsus marinus*	Dinophyta
	At2g44120	6E-72	320C026414	47	60s ribosomal protein	XP_002775844	1E-78	*Perkinsus marinus*	Dinophyta
	At2g44120	6E-72	320C026414	47	60s ribosomal protein	XP_002775844	1E-78	*Perkinsus marinus*	Dinophyta
60S ribosomal protein L8	At2g18020	5E-88	320C000277	93	60s ribosomal protein l8	XP_003882352	5E-125	*Neospora caninum*	Apicomplexa
	At2g18020	1E-94	320C000522	19	Ribosomal protein l8	XP_003882352	6E-133	*Neospora caninum*	Apicomplexa
	At2g18020	9E-90	320C000570	32	60s ribosomal protein l2	XP_003882352	3E-127	*Neospora caninum*	Apicomplexa
	At2g18020	1E-86	320C000652	15	Ribosomal protein l8	XP_003882352	3E-123	*Neospora caninum*	Apicomplexa
	At2g18020	7E-92	320C000759	26	60s ribosomal protein l8	XP_003882352	5E-128	*Neospora caninum*	Apicomplexa
	At2g18020	8E-86	320C000764	128	Ribosomal protein l8	EFN56373	4E-94	*Chlorella variabilis*	Chlorophyta
	At2g18020	2E-77	320C002323	21	60s ribosomal protein l8	ABV22452	3E-108	*Oxyrrhis marina*	Dinophyta
	At2g18020	2E-94	320C003240	32	60s ribosomal protein l8	XP_003882352	7E-131	*Neospora caninum*	Apicomplexa
	At2g18020	7E-58	320C016402	8	60s ribosomal protein l8-like	ACD65160	1E-71	*Phoronis muelleri*	Phoronida
	At2g18020	7E-58	320C016402	8	60s ribosomal protein l8-like	ACD65160	1E-71	*Phoronis muelleri*	Phoronida
	At2g18020	1E-94	320C020837	35	Ribosomal protein l8	XP_003882352	3E-133	*Neospora caninum*	Apicomplexa
	At2g18020	2E-77	320C021506	46	60s ribosomal protein l8	ABV22452	1E-108	*Oxyrrhis marina*	Dinophyta
60S ribosomal protein L9	At1g33120	4E-51	320C018869	20	60s ribosomal protein	XP_003881755	7E-61	*Neospora caninum*	Apicomplexa
DNA-directed RNA polymerase II	At4g35800	5E-51	320C003441	5	Dna-directed rna polymerase ii	XP_002767562	6E-68	*Perkinsus marinus*	Dinophyta

					largest				
	At4g35800	5E-51	320C003441	5	Dna-directed rna polymerase ii largest	XP_002767562	6E-68	*Perkinsus marinus*	Dinophyta
Importin beta	At5g53480	6E-95	320C012707	24	Importin subunit beta-1-like	XP_002778966	3E-150	*Perkinsus marinus*	Dinophyta
Mago Nashi-related protein	At1g02140	4E-67	320C002016	7	Mago nashi	XP_003880834	3E-90	*Neospora caninum*	Apicomplexa
	At1g02140	1E-58	320C002981	4	Protein mago nashi homolog 2	XP_002368810	1E-78	*Toxoplasma gondii*	Apicomplexa
	At1g02140	4E-67	320C005247	14	Mago nashi	XP_003880834	1E-90	*Neospora caninum*	Apicomplexa
Pre-mrna-splicing factor	At1g09770	5E-131	320C013276	13	Cdc5-like protein	EEE27401	7E-110	*Toxoplasma gondii*	Apicomplexa
	At2g33340	1E-103	320C015101	59	U-box domain-containing protein 72-like	XP_003880565	2E-139	*Neospora caninum*	Apicomplexa
Protein disulfide isomerase	At5g60640	3E-51	320C006766	15	Protein disulfide isomerase	XP_002788655	5E-122	*Perkinsus marinus*	Dinophyta
	At5g60640	6E-52	320C021003	21	Protein disulfide isomerase	XP_002788655	1E-103	*Perkinsus marinus*	Dinophyta
Ribosomal protein L2	68258.m00061	5E-52	320C014760	3	50s ribosomal protein l2	ZP_01910646	3E-100	*Plesiocystis pacifica*	Proteobacteria
Rubisco subunit binding-protein	At1g55490	9E-67	320C002228	6	Chaperonin	YP_006408688	7E-125	*Belliella baltica*	Bacteroidetes
	At1g55490	2E-52	320C012376	7	Chaperonin	YP_007099300	3E-54	*Chamaesiphon minutus*	Cyanobacteria
	At1g55490	2E-52	320C012376	7	Chaperonin	YP_007099300	3E-54	*Chamaesiphon minutus*	Cyanobacteria
	At1g55490	2E-54	320C013802	11	Heat shock protein 60	XP_002778119	5E-98	*Perkinsus marinus*	Dinophyta

	At1g55490	7E-134	320C017440	42	Chaperonin cpn60	EGZ08749	0	*Phytophthora sojae*	Oomycota
	At1g55490	7E-134	320C017440	42	Chaperonin cpn60	EGZ08749	0	*Phytophthora sojae*	Oomycota
	At1g55490	7E-79	320C018734	21	Heat shock protein	XP_002785716	4E-139	*Perkinsus marinus*	Dinophyta
	At1g55490	1E-152	320C019442	162	Chaperonin	ZP_01905725	0	*Plesiocystis pacifica*	Proteobacteria
	At1g55490	4E-78	320C019688	21	Heat shock protein	XP_002778119	4E-147	*Perkinsus marinus*	Dinophyta
	At1g55490	6E-140	320C023666	35	Chaperonin	EIE22221	0	*Coccomyxa subellipsoidea*	Chlorophyta
	At1g55490	6E-140	320C023666	35	Chaperonin	EIE22221	0	*Coccomyxa subellipsoidea*	Chlorophyta
	At1g55490	1E-86	320C024434	21	Chaperonin	ZP_01443429	0	*Pelagibaca bermudensis*	Proteobacteria
Splicing factor 3B subunit 1	At5g64270	7E-153	320C003248	15	Splicing factor 3b subunit 1-like	NP_001045876	1E-175	*Oryza sativa*	Streptophyta
	At5g64270	1E-80	320C014221	20	Splicing factor 3b subunit	XP_001199634	4E-96	*Strongylocentrotu s purpuratus*	Echinodermata
	At5g64270	2E-66	320C015562	7	Splicing factor 3b subunit	XP_002787169	5E-80	*Perkinsus marinus*	Dinophyta
Splicing factor 3B subunit 3	At3g55200	1E-76	320C002122	12	Splicing factor 3b subunit 3	XP_003883379	3E-94	*Neospora caninum*	Apicomplexa
	At3g55200	8E-83	320C014527	24	Splicing factor 3b subunit 4	XP_003883379	9E-97	*Neospora caninum*	Apicomplexa
	At3g55200	8E-54	320C021366	3	Splicing factor 3b subunit 3-like	XP_002767276	1E-63	*Perkinsus marinus*	Dinophyta
Splicing factor Prp8	At1g80070	5E-54	320C004543	3	Pre-mrna splicing factor	ABV81470	7E-65	*Tanystylum orbiculare*	Arthropoda
	At1g80070	2E-155	320C008393	51	Pre-mrna-processing-splicing factor 8	AAH34648	0	*Mus musculus*	Chordata
	At1g80070	0	320C021061	70	Pre-mrna-processing-splicing factor	XP_002368068	0	*Toxoplasma gondii*	Apicomplexa

						8				
Heat shock	Heat shock protein 90	At2g04030	2E-87	320C001927	16	Heat shock protein 90	ZP_01910552	0	*Plesiocystis pacifica*	Proteobacteria
		At2g04030	8E-52	320C002161	35	Heat shock protein 90	AAM02974	2E-108	*Crypthecodinium cohnii*	Dinophyta
		At2g04030	6E-87	320C003928	63	Heat shock protein	XP_002786497	0	*Perkinsus marinus*	Dinophyta
		At2g04030	8E-171	320C004975	95	Heat shock protein 90	AAM90674	0	*Achlya ambisexualis*	Oomycota
		At2g04030	1E-162	320C014520	46	Heat shock protein 90	EGB09597	0	*Aureococcus anophagefferens*	Heterokontophyta
		At2g04030	1E-56	320C015456	6	Endoplasmin homolog	BAJ89816	5E-80	*Hordeum vulgare*	Streptophyta
		At2g04030	2E-175	320C017797	154	Heat shock protein 90	XP_002998541	0	*Phytophthora infestans*	Oomycota
		At2g04030	2E-159	320C019708	348	Heat shock protein 90	XP_002784227	0	*Perkinsus marinus*	Dinophyta
		At2g04030	7E-91	320C023255	26	Heat shock protein 90	AAM02974	0	*Crypthecodinium cohnii*	Dinophyta
		At2g04030	1E-51	320C023676	8	Heat shock protein 90	ABA28985	1E-135	*Symbiodinium* sp.	Dinophyta
		At2g04030	1E-51	320C023676	8	Heat shock protein 90	ABA28985	1E-135	*Symbiodinium* sp.	Dinophyta
	Chaperonin (HSP60-2)	At2g33210	5E-65	320C000871	7	Chaperonin	ZP_02187726	8E-108	*Alpha proteobacterium*	Proteobacteria
		At2g33210	2E-72	320C002228	6	Chaperonin	YP_006408688	7E-125	*Belliella baltica*	Bacteroidetes
		At2g33210	6E-67	320C013802	11	Heat shock protein 60	XP_002778119	5E-98	*Perkinsus marinus*	Dinophyta
		At2g33210	3E-62	320C014665	11	Chaperonin	ZP_01880282	4E-93	*Roseovarius* sp.	Proteobacteria
		At2g33210	2E-64	320C014980	8	Chaperonin	ZP_05742950	7E-111	*Silicibacter* sp.	Proteobacteria
		At2g33210	2E-68	320C017440	42	Chaperonin cpn60 precursor	EGZ08749	0	*Phytophthora sojae*	Oomycota
		At2g33210	3E-119	320C017440	42	Chaperonin cpn60 precursor	EGZ08749	0	*Phytophthora sojae*	Oomycota

	At2g33210	3E-110	320C018734	21	Heat shock protein	XP_002785716	4E-139	*Perkinsus marinus*	Dinophyta
	At2g33210	4E-174	320C019442	162	Chaperonin	ZP_01905725	0	*Plesiocystis pacifica*	Proteobacteria
	At2g33210	4E-114	320C019688	21	Heat shock protein	XP_002778119	4E-147	*Perkinsus marinus*	Dinophyta
	At2g33210	5E-61	320C020498	10	Heat shock protein	XP_002765682	5E-86	*Perkinsus marinus*	Dinophyta
	At2g33210	0	320C023666	35	Chaperonin	EIE22221	0	*Coccomyxa subellipsoidea*	Chlorophyta
	At2g33210	0	320C023666	35	Chaperonin	EIE22221	0	*Coccomyxa subellipsoidea*	Chlorophyta
	At2g33210	3E-98	320C024434	21	Chaperonin	ZP_01443429	0	*Pelagibaca bermudensis*	Proteobacteria
	At2g33210	6E-53	320C029020	27	Heat shock protein	XP_002778476	1E-62	*Perkinsus marinus*	Dinophyta
Chaperonin, cpn60	At5g56500	6E-66	320C002228	6	Chaperonin	YP_006408688	7E-125	*Belliella baltica*	Bacteroidetes
	At5g56500	1E-54	320C013802	11	Heat shock protein 60	XP_002778119	5E-98	*Perkinsus marinus*	Dinophyta
	At5g56500	3E-135	320C017440	42	Chaperonin cpn60	EGZ08749	0	*Phytophthora sojae*	Oomycota
	At5g56500	3E-135	320C017440	42	Chaperonin cpn60	EGZ08749	0	*Phytophthora sojae*	Oomycota
	At5g56500	5E-80	320C018734	21	Heat shock protein	XP_002785716	4E-139	*Perkinsus marinus*	Dinophyta
	At5g56500	2E-153	320C019442	162	Chaperonin	ZP_01905725	0	*Plesiocystis pacifica*	Proteobacteria
	At5g56500	1E-79	320C019688	21	Heat shock protein	XP_002778119	4E-147	*Perkinsus marinus*	Dinophyta
	At5g56500	6E-140	320C023666	35	Chaperonin	EIE22221	0	*Coccomyxa subellipsoidea*	Chlorophyta
	At5g56500	6E-140	320C023666	35	Chaperonin	EIE22221	0	*Coccomyxa subellipsoidea*	Chlorophyta
	At5g56500	2E-86	320C024434	21	Chaperonin	ZP_01443429	0	*Pelagibaca bermudensis*	Proteobacteria
Dnaj protein	At4g39960	2E-51	320C013958	13	Dnaj protein	AFZ78979	2E-76	*Babesia equi*	Apicomplexa
Heat shock protein cphsc70-1	At4g24280	0	320C000044	85	Chaperone protein	ZP_01906286	0	*Plesiocystis pacifica*	Proteobacteria
	At4g24280	6E-54	320C000266	3	Chaperone	ZP_05785977	1E-110	*Silicibacter*	Proteobacteria

				protein			*lacuscaerulensis*	
At4g24280	1E-150	320C000309	382	Heat shock protein	XP_002780415	0	*Perkinsus marinus*	Dinophyta
At4g24280	2E-107	320C000520	42	Heat shock protein 70	P41753	0	*Achlya klebsiana*	Oomycota
At4g24280	1E-138	320C000629	38	Heat shock protein	AAM02971	0	*Crypthecodinium cohnii*	Dinophyta
At4g24280	2E-99	320C000813	48	Heat shock protein 70kd	XP_001713581	1E-149	*Guillardia theta*	Cryptophyta
At4g24280	4E-145	320C000905	43	Luminal-binding protein 5-like	CBJ48460	0	*Ectocarpus siliculosus*	Heterokontophyta
At4g24280	0	320C001247	81	Chaperone protein	XP_002765356	0	*Perkinsus marinus*	Dinophyta
At4g24280	2E-111	320C001316	36	Heat shock protein chaperone	CBJ48460	0	*Ectocarpus siliculosus*	Heterokontophyta
At4g24280	8E-156	320C002021	81	Heat shock protein 70	AAR21576	0	*Phytophthora nicotianae*	Oomycota
At4g24280	6E-148	320C002105	41	Heat shock protein	AAM02973	0	*Crypthecodinium cohnii*	Dinophyta
At4g24280	3E-109	320C007085	36	Heat shock protein	AAM02973	0	*Crypthecodinium cohnii*	Dinophyta
At4g24280	2E-79	320C007130	24	Heat shock protein	ACU45196	0	*Prorocentrum minimum*	Dinophyta
At4g24280	1E-147	320C013994	38	Heat shock protein	AAM02973	0	*Crypthecodinium cohnii*	Dinophyta
At4g24280	3E-139	320C016915	45	Heat shock protein	AAM02973	0	*Crypthecodinium cohnii*	Dinophyta
At4g24280	6E-52	320C018823	23	Heat shock protein	ABA28988	3E-92	*Symbiodinium* sp.	Dinophyta
At4g24280	5E-55	320C019201	23	Heat shock protein	AAM02971	2E-102	*Crypthecodinium cohnii*	Dinophyta
At4g24280	5E-55	320C019201	23	Heat shock protein	AAM02971	2E-102	*Crypthecodinium cohnii*	Dinophyta
At4g24280	0	320C019758	59	Chaperone	XP_002765356	0	*Perkinsus*	Dinophyta

					protein			*marinus*	
	At4g24280	0	320C019758	59	Chaperone protein	XP_002765356	0	*Perkinsus marinus*	Dinophyta
	At4g24280	2E-53	320C019869	20	Heat shock protein 70	AAM02971	9E-122	*Crypthecodinium cohnii*	Dinophyta
	At4g24280	4E-55	320C020021	3	Chaperone protein	YP_007706640	7E-94	*Octadecabacter antarcticus*	Proteobacteria
	At4g24280	4E-55	320C020021	3	Chaperone protein	YP_007706640	7E-94	*Octadecabacter antarcticus*	Proteobacteria
	At4g24280	4E-55	320C020021	3	Chaperone protein	YP_007706640	7E-94	*Octadecabacter antarcticus*	Proteobacteria
	At4g24280	7E-157	320C020757	82	Heat shock protein 70	AAM02971	0	*Crypthecodinium cohnii*	Dinophyta
	At4g24280	2E-142	320C022171	36	Heat shock protein	AAM02973	0	*Crypthecodinium cohnii*	Dinophyta
	At4g24280	3E-84	320C022502	17	Heat shock protein	XP_002780414	2E-149	*Perkinsus marinus*	Dinophyta
	At4g24280	1E-130	320C022780	21	Heat shock protein	AAM02971	0	*Crypthecodinium cohnii*	Dinophyta
	At4g24280	5E-151	320C023326	134	Heat shock protein	AAM02973	0	*Crypthecodinium cohnii*	Dinophyta
	At4g24280	5E-79	320C024364	8	Heat shock 70 kDa	XP_002898043	3E-121	*Phytophthora infestans*	Oomycota
	At4g24280	5E-79	320C024364	8	Heat shock 70 kDa	XP_002898043	3E-121	*Phytophthora infestans*	Oomycota
	At4g24280	5E-79	320C024364	8	Heat shock 70 kDa	XP_002898043	3E-121	*Phytophthora infestans*	Oomycota
	At4g24280	2E-56	320C024888	10	Heat shock 70 kDa protein 6	AAM02971	4E-68	*Crypthecodinium cohnii*	Dinophyta
	At4g24280	4E-64	320C026486	8	Stress-70	BAE73040	1E-80	*Macaca fascicularis*	Chordata
	At4g24280	4E-64	320C026486	8	Stress-70	BAE73040	1E-80	*Macaca fascicularis*	Chordata
	At4g24280	1E-62	320C027239	5	Chaperone protein	ZP_01901476	7E-98	*Roseobacter* sp.	Proteobacteria
	At4g24280	1E-62	320C027239	5	Chaperone protein	ZP_01901476	7E-98	*Roseobacter* sp.	Proteobacteria
	At4g24280	1E-52	320C027313	22	Heat shock protein	AAM02971	7E-94	*Crypthecodinium cohnii*	Dinophyta

	At4g24280	1E-66	320C030359	23	Heat shock protein	AAM02971	2E-150	*Crypthecodinium cohnii*	Dinophyta
Heat shock protein cphsc70-2	At5g49910	0	320C000044	85	Chaperone protein	ZP_01906286	0	*Plesiocystis pacifica*	Proteobacteria
	At5g49910	2E-54	320C000266	3	Chaperone protein	ZP_05785977	1E-110	*Silicibacter lacuscaerulensis*	Proteobacteria
	At5g49910	5E-147	320C000309	382	Heat shock protein	XP_002780415	0	*Perkinsus marinus*	Dinophyta
	At5g49910	3E-105	320C000520	42	Heat shock protein 70	P41753	0	*Achlya klebsiana*	Oomycota
	At5g49910	8E-139	320C000629	38	Heat shock protein	AAM02971	0	*Crypthecodinium cohnii*	Dinophyta
	At5g49910	3E-97	320C000813	48	Heat shock protein 70kd	XP_001713581	1E-149	*Guillardia theta*	Cryptophyta
	At5g49910	2E-142	320C000905	43	Luminal-binding protein 5-like	CBJ48460	0	*Ectocarpus siliculosus*	Heterokontophyta
	At5g49910	0	320C001247	81	Chaperone protein	XP_002765356	0	*Perkinsus marinus*	Dinophyta
	At5g49910	3E-110	320C001316	36	Heat shock protein chaperone	CBJ48460	0	*Ectocarpus siliculosus*	Heterokontophyta
	At5g49910	2E-151	320C002021	81	Heat shock protein 70	AAR21576	0	*Phytophthora nicotianae*	Oomycota
	At5g49910	3E-147	320C002105	41	Heat shock protein	AAM02973	0	*Crypthecodinium cohnii*	Dinophyta
	At5g49910	1E-105	320C007085	36	Heat shock protein	AAM02973	0	*Crypthecodinium cohnii*	Dinophyta
	At5g49910	2E-79	320C007130	24	Heat shock protein	ACU45196	0	*Prorocentrum minimum*	Dinophyta
	At5g49910	1E-146	320C013994	38	Heat shock protein	AAM02973	0	*Crypthecodinium cohnii*	Dinophyta
	At5g49910	6E-139	320C016915	45	Heat shock protein	AAM02973	0	*Crypthecodinium cohnii*	Dinophyta
	At5g49910	3E-52	320C018823	23	Heat shock protein	ABA28988	3E-92	*Symbiodinium* sp.	Dinophyta

At5g49910	5E-55	320C019201	23	Heat shock protein	AAM02971	2E-102	*Crypthecodinium cohnii*	Dinophyta
At5g49910	5E-55	320C019201	23	Heat shock protein	AAM02971	2E-102	*Crypthecodinium cohnii*	Dinophyta
At5g49910	0	320C019758	59	Chaperone protein	XP_002765356	0	*Perkinsus marinus*	Dinophyta
At5g49910	1E-52	320C019869	20	Heat shock protein 70	AAM02971	9E-122	*Crypthecodinium cohnii*	Dinophyta
At5g49910	4E-55	320C020021	3	Chaperone protein	YP_007706640	7E-94	*Octadecabacter antarcticus*	Proteobacteria
At5g49910	4E-55	320C020021	3	Chaperone protein	YP_007706640	7E-94	*Octadecabacter antarcticus*	Proteobacteria
At5g49910	4E-55	320C020021	3	Chaperone protein	YP_007706640	7E-94	*Octadecabacter antarcticus*	Proteobacteria
At5g49910	8E-155	320C020757	82	Heat shock protein 70	AAM02971	0	*Crypthecodinium cohnii*	Dinophyta
At5g49910	4E-141	320C022171	36	Heat shock protein	AAM02973	0	*Crypthecodinium cohnii*	Dinophyta
At5g49910	1E-83	320C022502	17	Heat shock protein	XP_002780414	2E-149	*Perkinsus marinus*	Dinophyta
At5g49910	8E-130	320C022780	21	Heat shock protein	AAM02971	0	*Crypthecodinium cohnii*	Dinophyta
At5g49910	3E-149	320C023326	134	Heat shock protein	AAM02973	0	*Crypthecodinium cohnii*	Dinophyta
At5g49910	6E-78	320C024364	8	Heat shock 70 kDa	XP_002898043	3E-121	*Phytophthora infestans*	Oomycota
At5g49910	6E-78	320C024364	8	Heat shock 70 kDa	XP_002898043	3E-121	*Phytophthora infestans*	Oomycota
At5g49910	6E-78	320C024364	8	Heat shock 70 kDa	XP_002898043	3E-121	*Phytophthora infestans*	Oomycota
At5g49910	1E-56	320C024888	10	Heat shock 70 kDa protein 6	AAM02971	4E-68	*Crypthecodinium cohnii*	Dinophyta
At5g49910	8E-63	320C026486	8	Stress-70	BAE73040	1E-80	*Macaca fascicularis*	Chordata
At5g49910	8E-63	320C026486	8	Stress-70	BAE73040	1E-80	*Macaca fascicularis*	Chordata
At5g49910	3E-62	320C027239	5	Chaperone protein	ZP_01901476	7E-98	*Roseobacter* sp.	Proteobacteria
At5g49910	3E-62	320C027239	5	Chaperone	ZP_01901476	7E-98	*Roseobacter* sp.	Proteobacteria

					protein				
	At5g49910	3E-53	320C027313	22	Heat shock protein	AAM02971	7E-94	*Crypthecodinium cohnii*	Dinophyta
	At5g49910	2E-67	320C030359	23	Heat shock protein	AAM02971	2E-150	*Crypthecodinium cohnii*	Dinophyta
Heat shock protein hsc70-1	At5g02500	1E-151	320C000044	85	Chaperone protein	ZP_01906286	0	*Plesiocystis pacifica*	Proteobacteria
	At5g02500	1E-53	320C000266	3	Chaperone protein	ZP_05785977	1E-110	*Silicibacter lacuscaerulensis*	Proteobacteria
	At5g02500	0	320C000309	382	Heat shock protein	XP_002780415	0	*Perkinsus marinus*	Dinophyta
	At5g02500	0	320C000520	42	Heat shock protein 70	P41753	0	*Achlya klebsiana*	Oomycota
	At5g02500	0	320C000629	38	Heat shock protein	AAM02971	0	*Crypthecodinium cohnii*	Dinophyta
	At5g02500	2E-126	320C000813	48	Heat shock protein 70kd	XP_001713581	1E-149	*Guillardia theta*	Cryptophyta
	At5g02500	0	320C000905	43	Luminal-binding protein 5-like	CBJ48460	0	*Ectocarpus siliculosus*	Heterokontophyta
	At5g02500	8E-156	320C001247	81	Chaperone protein	XP_002765356	0	*Perkinsus marinus*	Dinophyta
	At5g02500	9E-161	320C001316	36	Heat shock protein chaperone	CBJ48460	0	*Ectocarpus siliculosus*	Heterokontophyta
	At5g02500	6E-75	320C001896	17	Heat shock protein	AAM02973	3E-106	*Crypthecodinium cohnii*	Dinophyta
	At5g02500	6E-75	320C001896	17	Heat shock protein	AAM02973	3E-106	*Crypthecodinium cohnii*	Dinophyta
	At5g02500	3E-80	320C001992	14	Heat shock protein	AAM02973	9E-124	*Crypthecodinium cohnii*	Dinophyta
	At5g02500	0	320C002021	81	Heat shock protein 70	AAR21576	0	*Phytophthora nicotianae*	Oomycota
	At5g02500	0	320C002105	41	Heat shock protein	AAM02973	0	*Crypthecodinium cohnii*	Dinophyta
	At5g02500	6E-70	320C002126	4	Heat shock	EJK51576	1E-80	*Thalassiosira*	Heterokontophyta

					protein 70			*oceanica*	
	At5g02500	6E-70	320C002126	4	Heat shock protein 70	EJK51576	1E-80	*Thalassiosira oceanica*	Heterokontophyta
	At5g02500	2E-56	320C003591	3	Heat shock protein partial	AAW58103	2E-71	*Heterocapsa triquetra*	Dinophyta
	At5g02500	0	320C007085	36	Heat shock protein	AAM02973	0	*Crypthecodinium cohnii*	Dinophyta
	At5g02500	1E-131	320C007130	24	Heat shock protein	ACU45196	0	*Prorocentrum minimum*	Dinophyta
	At5g02500	9E-54	320C009212	13	Heat shock protein	AET50612	7E-60	*Eimeria tenella*	Apicomplexa
	At5g02500	2E-58	320C011902	23	Hsp70-like protein	CBJ30106	9E-79	*Ectocarpus siliculosus*	Heterokontophyta
	At5g02500	5E-57	320C012240	12	Heat shock protein 70	ABA28988	2E-79	*Symbiodinium* sp.	Dinophyta
	At5g02500	0	320C013994	38	Heat shock protein	AAM02973	0	*Crypthecodinium cohnii*	Dinophyta
	At5g02500	4E-59	320C016014	23	Heat shock protein	AAM02971	3E-101	*Crypthecodinium cohnii*	Dinophyta
	At5g02500	0	320C016915	45	Heat shock protein	AAM02973	0	*Crypthecodinium cohnii*	Dinophyta
	At5g02500	4E-57	320C018558	16	Heat shock protein	ABA28988	2E-79	*Symbiodinium* sp.	Dinophyta
	At5g02500	1E-56	320C018640	36	Heat shock protein	AAM02973	1E-87	*Crypthecodinium cohnii*	Dinophyta
	At5g02500	2E-72	320C018823	23	Heat shock protein	ABA28988	3E-92	*Symbiodinium* sp.	Dinophyta
	At5g02500	1E-61	320C018859	18	Heat shock protein 70	AAR21576	1E-82	*Phytophthora nicotianae*	Oomycota
	At5g02500	7E-73	320C019201	23	Heat shock protein	AAM02971	2E-102	*Crypthecodinium cohnii*	Dinophyta
	At5g02500	7E-73	320C019201	23	Heat shock protein	AAM02971	2E-102	*Crypthecodinium cohnii*	Dinophyta
	At5g02500	4E-149	320C019758	59	Chaperone protein	XP_002765356	0	*Perkinsus marinus*	Dinophyta
	At5g02500	5E-69	320C019869	20	Heat shock protein 70	AAM02971	9E-122	*Crypthecodinium cohnii*	Dinophyta
	At5g02500	0	320C020757	82	Heat shock protein 70	AAM02971	0	*Crypthecodinium cohnii*	Dinophyta

At5g02500	1E-55	320C021369	2	Heat shock protein 70	EJK51576	6E-70	*Thalassiosira oceanica*	Heterokontophyta
At5g02500	0	320C022171	36	Heat shock protein	AAM02973	0	*Crypthecodinium cohnii*	Dinophyta
At5g02500	7E-117	320C022502	17	Heat shock protein	XP_002780414	2E-149	*Perkinsus marinus*	Dinophyta
At5g02500	1E-74	320C022503	5	Heat shock protein	XP_002780414	8E-101	*Perkinsus marinus*	Dinophyta
At5g02500	1E-77	320C022707	17	Heat shock protein hsp70 family protein	AER57864	5E-102	*Acytostelium subglobosum*	Amoebozoa
At5g02500	1E-170	320C022780	21	Heat shock protein	AAM02971	0	*Crypthecodinium cohnii*	Dinophyta
At5g02500	0	320C023326	134	Heat shock protein	AAM02973	0	*Crypthecodinium cohnii*	Dinophyta
At5g02500	1E-65	320C024364	8	Heat shock 70 kDa	XP_002898043	3E-121	*Phytophthora infestans*	Oomycota
At5g02500	1E-65	320C024364	8	Heat shock 70 kDa	XP_002898043	3E-121	*Phytophthora infestans*	Oomycota
At5g02500	1E-65	320C024364	8	Heat shock 70 kDa	XP_002898043	3E-121	*Phytophthora infestans*	Oomycota
At5g02500	4E-65	320C024888	10	Heat shock 70 kDa protein 6	AAM02971	4E-68	*Crypthecodinium cohnii*	Dinophyta
At5g02500	4E-64	320C025398	6	Heat shock protein	CCD13252	2E-87	*Trypanosoma congolense*	Euglenozoa
At5g02500	6E-85	320C025399	18	Heat shock protein 70 kDa	ACU17965	1E-75	*Glycine max*	Streptophyta
At5g02500	1E-51	320C026486	8	Stress-70	BAE73040	1E-80	*Macaca fascicularis*	Chordata
At5g02500	1E-51	320C026486	8	Stress-70	BAE73040	1E-80	*Macaca fascicularis*	Chordata
At5g02500	8E-66	320C027313	22	Heat shock protein	AAM02971	7E-94	*Crypthecodinium cohnii*	Dinophyta
At5g02500	3E-60	320C027836	13	Heat shock protein 70	BAF62730	7E-76	*Paracoccidioides brasiliensis*	Ascomycota
At5g02500	4E-76	320C029055	17	Heat shock	ACU17965	2E-67	*Glycine max*	Streptophyta

					protein 70 kDa				
	At5g02500	4E-88	320C030359	23	Heat shock protein	AAM02971	2E-150	*Crypthecodinium cohnii*	Dinophyta
Heat shock protein hsp70	At4g16660	2E-60	320C000309	382	Heat shock protein	XP_002780415	0	*Perkinsus marinus*	Dinophyta
	At4g16660	6E-53	320C000520	42	Heat shock protein 70	P41753	0	*Achlya klebsiana*	Oomycota
	At4g16660	9E-58	320C000629	38	Heat shock protein	AAM02971	0	*Crypthecodinium cohnii*	Dinophyta
	At4g16660	2E-52	320C000905	43	Luminal-binding protein 5-like	CBJ48460	0	*Ectocarpus siliculosus*	Heterokontophyta
	At4g16660	3E-58	320C001537	55	Heat shock protein	EOA18958	3E-49	*Capsella rubella*	Streptophyta
	At4g16660	6E-55	320C002021	81	Heat shock protein 70	AAR21576	0	*Phytophthora nicotianae*	Oomycota
	At4g16660	2E-55	320C002105	41	Heat shock protein	AAM02973	0	*Crypthecodinium cohnii*	Dinophyta
	At4g16660	4E-55	320C013994	38	Heat shock protein	AAM02973	0	*Crypthecodinium cohnii*	Dinophyta
	At4g16660	2E-55	320C016915	45	Heat shock protein	AAM02973	0	*Crypthecodinium cohnii*	Dinophyta
	At4g16660	4E-59	320C020757	82	Heat shock protein 70	AAM02971	0	*Crypthecodinium cohnii*	Dinophyta
	At4g16660	1E-54	320C022171	36	Heat shock protein	AAM02973	0	*Crypthecodinium cohnii*	Dinophyta
	At4g16660	1E-55	320C023326	134	Heat shock protein	AAM02973	0	*Crypthecodinium cohnii*	Dinophyta
Heat shock protein hsp70b	At1g16030	4E-54	320C000266	3	Chaperone protein	ZP_05785977	1E-110	*Silicibacter lacuscaerulensis*	Proteobacteria
	At1g16030	5E-146	320C000044	85	Chaperone protein	ZP_01906286	0	*Plesiocystis pacifica*	Proteobacteria
	At1g16030	0	320C000309	382	Heat shock protein	XP_002780415	0	*Perkinsus marinus*	Dinophyta
	At1g16030	0	320C000520	42	Heat shock protein 70	P41753	0	*Achlya klebsiana*	Oomycota
	At1g16030	0	320C000629	38	Heat shock protein	AAM02971	0	*Crypthecodinium cohnii*	Dinophyta
	At1g16030	9E-127	320C000813	48	Heat shock	XP_001713581	1E-149	*Guillardia theta*	Cryptophyta

					protein 70kd				
	At1g16030	0	320C000905	43	Luminal-binding protein 5-like	CBJ48460	0	*Ectocarpus siliculosus*	Heterokontophyta
	At1g16030	1E-153	320C001247	81	Chaperone protein	XP_002765356	0	*Perkinsus marinus*	Dinophyta
	At1g16030	2E-154	320C001316	36	Heat shock protein chaperone	CBJ48460	0	*Ectocarpus siliculosus*	Heterokontophyta
	At1g16030	8E-78	320C001896	17	Heat shock protein	AAM02973	3E-106	*Crypthecodinium cohnii*	Dinophyta
	At1g16030	8E-78	320C001896	17	Heat shock protein	AAM02973	3E-106	*Crypthecodinium cohnii*	Dinophyta
	At1g16030	5E-83	320C001992	14	Heat shock protein	AAM02973	9E-124	*Crypthecodinium cohnii*	Dinophyta
	At1g16030	0	320C002021	81	Heat shock protein 70	AAR21576	0	*Phytophthora nicotianae*	Oomycota
	At1g16030	0	320C002105	41	Heat shock protein	AAM02973	0	*Crypthecodinium cohnii*	Dinophyta
	At1g16030	3E-70	320C002126	4	Heat shock protein 70	EJK51576	1E-80	*Thalassiosira oceanica*	Heterokontophyta
	At1g16030	3E-70	320C002126	4	Heat shock protein 70	EJK51576	1E-80	*Thalassiosira oceanica*	Heterokontophyta
	At1g16030	1E-56	320C003591	3	Heat shock protein partial	AAW58103	2E-71	*Heterocapsa triquetra*	Dinophyta
	At1g16030	0	320C007085	36	Heat shock protein	AAM02973	0	*Crypthecodinium cohnii*	Dinophyta
	At1g16030	1E-127	320C007130	24	Heat shock protein	ACU45196	0	*Prorocentrum minimum*	Dinophyta
	At1g16030	5E-51	320C009212	13	Heat shock protein	AET50612	7E-60	*Eimeria tenella*	Apicomplexa
	At1g16030	5E-59	320C011902	23	Hsp70-like protein	CBJ30106	9E-79	*Ectocarpus siliculosus*	Heterokontophyta
	At1g16030	2E-54	320C012240	12	Heat shock protein 70	ABA28988	2E-79	*Symbiodinium* sp.	Dinophyta
	At1g16030	0	320C013994	38	Heat shock	AAM02973	0	*Crypthecodinium*	Dinophyta

				protein			*cohnii*	
At1g16030	1E-58	320C016014	23	Heat shock protein	AAM02971	3E-101	*Crypthecodinium cohnii*	Dinophyta
At1g16030	0	320C016915	45	Heat shock protein	AAM02973	0	*Crypthecodinium cohnii*	Dinophyta
At1g16030	2E-54	320C018558	16	Heat shock protein	ABA28988	2E-79	*Symbiodinium* sp.	Dinophyta
At1g16030	8E-60	320C018640	36	Heat shock protein	AAM02973	1E-87	*Crypthecodinium cohnii*	Dinophyta
At1g16030	2E-71	320C018823	23	Heat shock protein	ABA28988	3E-92	*Symbiodinium* sp.	Dinophyta
At1g16030	5E-59	320C018859	18	Heat shock protein 70	AAR21576	1E-82	*Phytophthora nicotianae*	Oomycota
At1g16030	5E-59	320C018859	18	Heat shock protein 70	AAR21576	1E-82	*Phytophthora nicotianae*	Oomycota
At1g16030	1E-70	320C019201	23	Heat shock protein 70	AAM02971	2E-102	*Crypthecodinium cohnii*	Dinophyta
At1g16030	1E-70	320C019201	23	Heat shock protein 70	AAM02971	2E-102	*Crypthecodinium cohnii*	Dinophyta
At1g16030	8E-146	320C019758	59	Chaperone protein	XP_002765356	0	*Perkinsus marinus*	Dinophyta
At1g16030	8E-146	320C019758	59	Chaperone protein	XP_002765356	0	*Perkinsus marinus*	Dinophyta
At1g16030	1E-67	320C019869	20	Heat shock protein 70	AAM02971	9E-122	*Crypthecodinium cohnii*	Dinophyta
At1g16030	0	320C020757	82	Heat shock protein 70	AAM02971	0	*Crypthecodinium cohnii*	Dinophyta
At1g16030	4E-58	320C021369	2	Heat shock protein 70	EJK51576	6E-70	*Thalassiosira oceanica*	Heterokontophyta
At1g16030	4E-58	320C021369	2	Heat shock protein 70	EJK51576	6E-70	*Thalassiosira oceanica*	Heterokontophyta
At1g16030	0	320C022171	36	Heat shock protein	AAM02973	0	*Crypthecodinium cohnii*	Dinophyta
At1g16030	3E-115	320C022502	17	Heat shock protein	XP_002780414	2E-149	*Perkinsus marinus*	Dinophyta
At1g16030	7E-80	320C022503	5	Heat shock protein	XP_002780414	8E-101	*Perkinsus marinus*	Dinophyta
At1g16030	7E-76	320C022707	17	Heat shock protein hsp70	AER57864	5E-102	*Acytostelium subglobosum*	Amoebozoa

					family protein				
	At1g16030	1E-168	320C022780	21	Heat shock protein	AAM02971	0	*Crypthecodinium cohnii*	Dinophyta
	At1g16030	0	320C023326	134	Heat shock protein	AAM02973	0	*Crypthecodinium cohnii*	Dinophyta
	At1g16030	1E-67	320C024888	10	Heat shock 70 kDa protein 6	AAM02971	4E-68	*Crypthecodinium cohnii*	Dinophyta
	At1g16030	3E-63	320C025398	6	Heat shock protein	CCD13252	2E-87	*Trypanosoma congolense*	Euglenozoa
	At1g16030	2E-77	320C025399	18	Heat shock protein 70 kDa	ACU17965	1E-75	*Glycine max*	Streptophyta
	At1g16030	2E-63	320C027313	22	Heat shock protein	AAM02971	7E-94	*Crypthecodinium cohnii*	Dinophyta
	At1g16030	2E-58	320C027836	13	Heat shock protein 70	BAF62730	7E-76	*Paracoccidioides brasiliensis*	Ascomycota
	At1g16030	3E-71	320C029055	17	Heat shock protein 70 kDa	ACU17965	2E-67	*Glycine max*	Streptophyta
	At1g16030	4E-85	320C030359	23	Heat shock protein	AAM02971	2E-150	*Crypthecodinium cohnii*	Dinophyta
Heat shock protein hsp70t-1	At1g56410	9E-146	320C000044	85	Chaperone protein	ZP_01906286	0	*Plesiocystis pacifica*	Proteobacteria
	At1g56410	3E-53	320C000266	3	Chaperone protein	ZP_05785977	1E-110	*Silicibacter lacuscaerulensis*	Proteobacteria
	At1g56410	0	320C000309	382	Heat shock protein	XP_002780415	0	*Perkinsus marinus*	Dinophyta
	At1g56410	0	320C000520	42	Heat shock protein 70	P41753	0	*Achlya klebsiana*	Oomycota
	At1g56410	0	320C000629	38	Heat shock protein	AAM02971	0	*Crypthecodinium cohnii*	Dinophyta
	At1g56410	5E-123	320C000813	48	Heat shock protein 70kd	XP_001713581	1E-149	*Guillardia theta*	Cryptophyta
	At1g56410	0	320C000905	43	Luminal-binding protein 5-like	CBJ48460	0	*Ectocarpus siliculosus*	Heterokontophyta
	At1g56410	1E-155	320C001247	81	Chaperone protein	XP_002765356	0	*Perkinsus marinus*	Dinophyta

	At1g56410	1E-150	320C001316	36	Heat shock protein chaperone	CBJ48460	0	*Ectocarpus siliculosus*	Heterokontophyta
	At1g56410	1E-75	320C001896	17	Heat shock protein	AAM02973	3E-106	*Crypthecodinium cohnii*	Dinophyta
	At1g56410	1E-75	320C001896	17	Heat shock protein	AAM02973	3E-106	*Crypthecodinium cohnii*	Dinophyta
	At1g56410	1E-80	320C001992	14	Heat shock protein	AAM02973	9E-124	*Crypthecodinium cohnii*	Dinophyta
	At1g56410	0	320C002021	81	Heat shock protein 70	AAR21576	0	*Phytophthora nicotianae*	Oomycota
	At1g56410	0	320C002105	41	Heat shock protein	AAM02973	0	*Crypthecodinium cohnii*	Dinophyta
	At1g56410	1E-68	320C002126	4	Heat shock protein 70	EJK51576	1E-80	*Thalassiosira oceanica*	Heterokontophyta
	At1g56410	1E-68	320C002126	4	Heat shock protein 70	EJK51576	1E-80	*Thalassiosira oceanica*	Heterokontophyta
	At1g56410	6E-57	320C003591	3	Heat shock protein partial	AAW58103	2E-71	*Heterocapsa triquetra*	Dinophyta
	At1g56410	3E-173	320C007085	36	Heat shock protein	AAM02973	0	*Crypthecodinium cohnii*	Dinophyta
	At1g56410	7E-126	320C007130	24	Heat shock protein	ACU45196	0	*Prorocentrum minimum*	Dinophyta
	At1g56410	1E-56	320C011902	23	Hsp70-like protein	CBJ30106	9E-79	*Ectocarpus siliculosus*	Heterokontophyta
	At1g56410	7E-52	320C012240	12	Heat shock protein 70	ABA28988	2E-79	*Symbiodinium* sp.	Dinophyta
	At1g56410	0	320C013994	38	Heat shock protein	AAM02973	0	*Crypthecodinium cohnii*	Dinophyta
	At1g56410	2E-60	320C016014	23	Heat shock protein	AAM02971	3E-101	*Crypthecodinium cohnii*	Dinophyta
	At1g56410	0	320C016915	45	Heat shock protein	AAM02973	0	*Crypthecodinium cohnii*	Dinophyta
	At1g56410	5E-52	320C018558	16	Heat shock protein	ABA28988	2E-79	*Symbiodinium* sp.	Dinophyta
	At1g56410	4E-57	320C018640	36	Heat shock protein	AAM02973	1E-87	*Crypthecodinium cohnii*	Dinophyta
	At1g56410	2E-67	320C018823	23	Heat shock protein	ABA28988	3E-92	*Symbiodinium* sp.	Dinophyta

	At1g56410	1E-52	320C018859	18	Heat shock protein 70	AAR21576	1E-82	*Phytophthora nicotianae*	Oomycota
	At1g56410	2E-67	320C019201	23	Heat shock protein	AAM02971	2E-102	*Crypthecodinium cohnii*	Dinophyta
	At1g56410	2E-67	320C019201	23	Heat shock protein	AAM02971	2E-102	*Crypthecodinium cohnii*	Dinophyta
	At1g56410	1E-150	320C019758	59	Chaperone protein	XP_002765356	0	*Perkinsus marinus*	Dinophyta
	At1g56410	4E-69	320C019869	20	Heat shock protein 70	AAM02971	9E-122	*Crypthecodinium cohnii*	Dinophyta
	At1g56410	0	320C020757	82	Heat shock protein 70	AAM02971	0	*Crypthecodinium cohnii*	Dinophyta
	At1g56410	2E-51	320C021369	2	Heat shock protein 70	EJK51576	6E-70	*Thalassiosira oceanica*	Heterokontophyta
	At1g56410	0	320C022171	36	Heat shock protein	AAM02973	0	*Crypthecodinium cohnii*	Dinophyta
	At1g56410	7E-109	320C022502	17	Heat shock protein	XP_002780414	2E-149	*Perkinsus marinus*	Dinophyta
	At1g56410	3E-76	320C022503	5	Heat shock protein	XP_002780414	8E-101	*Perkinsus marinus*	Dinophyta
	At1g56410	2E-69	320C022707	17	Heat shock protein hsp70 family protein	AER57864	5E-102	*Acytostelium subglobosum*	Amoebozoa
	At1g56410	2E-165	320C022780	21	Heat shock protein	AAM02971	0	*Crypthecodinium cohnii*	Dinophyta
	At1g56410	0	320C023326	134	Heat shock protein	AAM02973	0	*Crypthecodinium cohnii*	Dinophyta
	At1g56410	8E-67	320C024364	8	Heat shock 70 kDa	XP_002898043	3E-121	*Phytophthora infestans*	Oomycota
	At1g56410	8E-67	320C024364	8	Heat shock 70 kDa	XP_002898043	3E-121	*Phytophthora infestans*	Oomycota
	At1g56410	8E-67	320C024364	8	Heat shock 70 kDa	XP_002898043	3E-121	*Phytophthora infestans*	Oomycota
	At1g56410	5E-64	320C024888	10	Heat shock 70 kDa protein 6	AAM02971	4E-68	*Crypthecodinium cohnii*	Dinophyta
	At1g56410	8E-64	320C025398	6	Heat shock	CCD13252	2E-87	*Trypanosoma*	Euglenozoa

					protein			*congolense*	
	At1g56410	2E-68	320C025399	18	Heat shock protein 70 kDa	ACU17965	1E-75	*Glycine max*	Streptophyta
	At1g56410	1E-61	320C027313	22	Heat shock protein	AAM02971	7E-94	*Crypthecodinium cohnii*	Dinophyta
	At1g56410	6E-59	320C027836	13	Heat shock protein 70	BAF62730	7E-76	*Paracoccidioides brasiliensis*	Ascomycota
	At1g56410	2E-66	320C029055	17	Heat shock protein 70 kDa	ACU17965	2E-67	*Glycine max*	Streptophyta
	At1g56410	5E-84	320C030359	23	Heat shock protein	AAM02971	2E-150	*Crypthecodinium cohnii*	Dinophyta
Heat shock protein mthsc70-2	At5g09590	0	320C000044	85	Chaperone protein	ZP_01906286	0	*Plesiocystis pacifica*	Proteobacteria
	At5g09590	8E-69	320C000266	3	Chaperone protein	ZP_05785977	1E-110	*Silicibacter lacuscaerulensis*	Proteobacteria
	At5g09590	3E-154	320C000309	382	Heat shock protein	XP_002780415	0	*Perkinsus marinus*	Dinophyta
	At5g09590	1E-111	320C000520	42	Heat shock protein 70	P41753	0	*Achlya klebsiana*	Oomycota
	At5g09590	2E-146	320C000629	38	Heat shock protein	AAM02971	0	*Crypthecodinium cohnii*	Dinophyta
	At5g09590	5E-102	320C000813	48	Heat shock protein 70kd	XP_001713581	1E-149	*Guillardia theta*	Cryptophyta
	At5g09590	3E-149	320C000905	43	Luminal-binding protein 5-like	CBJ48460	0	*Ectocarpus siliculosus*	Heterokontophyta
	At5g09590	0	320C001247	81	Chaperone protein	XP_002765356	0	*Perkinsus marinus*	Dinophyta
	At5g09590	8E-114	320C001316	36	Heat shock protein chaperone	CBJ48460	0	*Ectocarpus siliculosus*	Heterokontophyta
	At5g09590	2E-162	320C002021	81	Heat shock protein 70	AAR21576	0	*Phytophthora nicotianae*	Oomycota
	At5g09590	1E-157	320C002105	41	Heat shock protein	AAM02973	0	*Crypthecodinium cohnii*	Dinophyta
	At5g09590	2E-107	320C007085	36	Heat shock protein	AAM02973	0	*Crypthecodinium cohnii*	Dinophyta
	At5g09590	3E-81	320C007130	24	Heat shock protein	ACU45196	0	*Prorocentrum minimum*	Dinophyta

	At5g09590	7E-51	320C011902	23	Hsp70-like protein	CBJ30106	9E-79	*Ectocarpus siliculosus*	Heterokontophyta
	At5g09590	3E-157	320C013994	38	Heat shock protein	AAM02973	0	*Crypthecodinium cohnii*	Dinophyta
	At5g09590	3E-150	320C016915	45	Heat shock protein	AAM02973	0	*Crypthecodinium cohnii*	Dinophyta
	At5g09590	1E-61	320C017405	9	Heat shock protein	EGZ18952	6E-79	*Phytophthora sojae*	Oomycota
	At5g09590	5E-54	320C018823	23	Heat shock protein	ABA28988	3E-92	*Symbiodinium* sp.	Dinophyta
	At5g09590	4E-59	320C019201	23	Heat shock protein	AAM02971	2E-102	*Crypthecodinium cohnii*	Dinophyta
	At5g09590	4E-59	320C019201	23	Heat shock protein	AAM02971	2E-102	*Crypthecodinium cohnii*	Dinophyta
	At5g09590	0	320C019758	59	Chaperone protein	XP_002765356	0	*Perkinsus marinus*	Dinophyta
	At5g09590	0	320C019758	59	Chaperone protein	XP_002765356	0	*Perkinsus marinus*	Dinophyta
	At5g09590	6E-52	320C019869	20	Heat shock protein 70	AAM02971	9E-122	*Crypthecodinium cohnii*	Dinophyta
	At5g09590	5E-62	320C020021	3	Chaperone protein	YP_007706640	7E-94	*Octadecabacter antarcticus*	Proteobacteria
	At5g09590	5E-62	320C020021	3	Chaperone protein	YP_007706640	7E-94	*Octadecabacter antarcticus*	Proteobacteria
	At5g09590	4E-168	320C020757	82	Heat shock protein 70	AAM02971	0	*Crypthecodinium cohnii*	Dinophyta
	At5g09590	1E-153	320C022171	36	Heat shock protein	AAM02973	0	*Crypthecodinium cohnii*	Dinophyta
	At5g09590	5E-83	320C022502	17	Heat shock protein	XP_002780414	2E-149	*Perkinsus marinus*	Dinophyta
	At5g09590	5E-54	320C022503	5	Heat shock protein	XP_002780414	8E-101	*Perkinsus marinus*	Dinophyta
	At5g09590	3E-135	320C022780	21	Heat shock protein	AAM02971	0	*Crypthecodinium cohnii*	Dinophyta
	At5g09590	5E-158	320C023326	134	Heat shock protein	AAM02973	0	*Crypthecodinium cohnii*	Dinophyta

	At5g09590	2E-108	320C024364	8	Heat shock 70 kDa	XP_002898043	3E-121	*Phytophthora infestans*	Oomycota
	At5g09590	2E-108	320C024364	8	Heat shock 70 kDa	XP_002898043	3E-121	*Phytophthora infestans*	Oomycota
	At5g09590	2E-108	320C024364	8	Heat shock 70 kDa	XP_002898043	3E-121	*Phytophthora infestans*	Oomycota
	At5g09590	6E-52	320C024888	10	Heat shock 70 kDa protein 6	AAM02971	4E-68	*Crypthecodinium cohnii*	Dinophyta
	At5g09590	6E-52	320C024888	10	Heat shock 70 kDa protein 6	AAM02971	4E-68	*Crypthecodinium cohnii*	Dinophyta
	At5g09590	3E-71	320C026486	8	Stress-70	BAE73040	1E-80	*Macaca fascicularis*	Chordata
	At5g09590	3E-71	320C026486	8	Stress-70	BAE73040	1E-80	*Macaca fascicularis*	Chordata
	At5g09590	3E-56	320C027313	22	Heat shock protein	AAM02971	7E-94	*Crypthecodinium cohnii*	Dinophyta
	At5g09590	2E-71	320C030359	23	Heat shock protein	AAM02971	2E-150	*Crypthecodinium cohnii*	Dinophyta
HSP100/clpb	At2g25140	0	320C001286	248	Heat shock protein 101	XP_002328643	0	*Populus trichocarpa*	Streptophyta
	At2g25140	0	320C005350	42	Clp protease ATP-binding subunit	CCA24481	0	*Albugo laibachii*	Oomycota
	At2g25140	1E-60	320C016224	8	Clp protease ATP binding subunit	XP_003063658	8E-91	*Micromonas pusilla*	Chlorophyta
	At2g25140	2E-168	320C018784	37	Heat shock protein 101	CCA14066	0	*Albugo laibachii*	Oomycota
	At2g25140	3E-71	320C024021	4	Heat shock protein 101	EGD82525	2E-104	*Salpingoeca* sp.	Choanozoa
	At2g25140	2E-53	320C024988	4	ATP-dependent clp protease ATP-binding subunit clpa homolog	YP_003002123	2E-61	*Aureococcus anophagefferens*	Heterokontophyta
	At2g25140	2E-53	320C024988	4	ATP-dependent clp protease ATP-	YP_003002123	2E-61	*Aureococcus anophagefferens*	Heterokontophyta

					binding subunit clpa homolog				
HSP90-like domain protein	At4g24190	3E-88	320C001927	16	Heat shock protein 90	ZP_01910552	0	*Plesiocystis pacifica*	Proteobacteria
	At4g24190	9E-51	320C002161	35	Heat shock protein 90	AAM02974	2E-108	*Crypthecodinium cohnii*	Dinophyta
	At4g24190	0	320C004975	95	Heat shock protein 90	AAM90674	0	*Achlya ambisexualis*	Oomycota
	At4g24190	0	320C014520	46	Heat shock protein 90	EGB09597	0	*Aureococcus anophagefferens*	Heterokontophyta
	At4g24190	9E-69	320C015456	6	Endoplasmin homolog	BAJ89816	5E-80	*Hordeum vulgare*	Streptophyta
	At4g24190	1E-51	320C015856	7	Heat shock protein 90	ABA28985	7E-107	*Symbiodinium* sp.	Dinophyta
	At4g24190	0	320C017797	154	Heat shock protein 90	XP_002998541	0	*Phytophthora infestans*	Oomycota
	At4g24190	5E-69	320C018372	18	Endoplasmin homolog	EGB09597	8E-71	*Aureococcus anophagefferens*	Heterokontophyta
	At4g24190	1E-52	320C019319	13	Heat shock protein 90	ABA28985	8E-91	*Symbiodinium* sp.	Dinophyta
	At4g24190	0	320C019708	348	Heat shock protein 90	XP_002784227	0	*Perkinsus marinus*	Dinophyta
	At4g24190	1E-93	320C023255	26	Heat shock protein 90	AAM02974	0	*Crypthecodinium cohnii*	Dinophyta
	At4g24190	2E-62	320C023676	8	Heat shock protein 90	ABA28985	1E-135	*Symbiodinium* sp.	Dinophyta
	At4g24190	2E-62	320C023676	8	Heat shock protein 90	ABA28985	1E-135	*Symbiodinium* sp.	Dinophyta
Rubisco subunit binding-protein alpha subunit/60 kDa chaperonin	At2g28000	2E-66	320C002228	6	Chaperonin	YP_006408688	7E-125	*Belliella baltica*	Bacteroidetes
	At2g28000	1E-56	320C012376	7	Chaperonin	YP_007099300	3E-54	*Chamaesiphon minutus*	Cyanobacteria
	At2g28000	1E-56	320C012376	7	Chaperonin	YP_007099300	3E-54	*Chamaesiphon minutus*	Cyanobacteria
	At2g28000	2E-53	320C013802	11	Heat shock protein 60	XP_002778119	5E-98	*Perkinsus marinus*	Dinophyta

	alpha subunit	At2g28000	1E-51	320C014980	8	Chaperonin	ZP_05742950	7E-111	*Silicibacter* sp.	Proteobacteria
		At2g28000	6E-84	320C017440	42	Chaperonin cpn60-	EGZ08749	0	*Phytophthora sojae*	Oomycota
		At2g28000	1E-78	320C018734	21	Heat shock protein	XP_002785716	4E-139	*Perkinsus marinus*	Dinophyta
		At2g28000	6E-147	320C019442	162	Chaperonin	ZP_01905725	0	*Plesiocystis pacifica*	Proteobacteria
		At2g28000	5E-78	320C019688	21	Heat shock protein	XP_002778119	4E-147	*Perkinsus marinus*	Dinophyta
		At2g28000	6E-140	320C023666	35	Chaperonin	EIE22221	0	*Coccomyxa subellipsoidea*	Chlorophyta
		At2g28000	6E-140	320C023666	35	Chaperonin	EIE22221	0	*Coccomyxa subellipsoidea*	Chlorophyta
		At2g28000	1E-88	320C024434	21	Chaperonin	ZP_01443429	0	*Pelagibaca bermudensis*	Proteobacteria
Total:		162 *A. thaliana* chloroplast proteins		544 contigs	15,689 reads					
